
JOURNAL INFORMATION
==============================
NLM Title Abbreviation: Innov Aging
Journal ID: Innov Aging
NLM Journal ID: 101703706
Journal ID: innovateage

Innovation in Aging

EISSN: 2399-5300
Publisher: Oxford University Press

ARTICLE INFORMATION
==============================
PMCID: PMC6845017
DOI: 10.1093/geroni/igz046
Article ID: igz046
Article version: 1
Subjects: Index

Index Participant

Publication date: 2019 Nov
Electronic publication date: 2019 Nov 8
Volume: 3
Issue: Suppl 1
Issue title: Program Abstracts from the GSA 2019 Annual Scientific Meeting “Strength in Age—Harnessing the Power of Networks”
First page: S982
Last page: S1021
Copyright: © The Author(s) 2019. Published by Oxford University Press on behalf of The Gerontological Society of America.
Copyright year: 2019
License: This is an Open Access article distributed under the terms of the Creative Commons Attribution License (http://creativecommons.org/licenses/by/4.0/), which permits unrestricted reuse, distribution, and reproduction in any medium, provided the original work is properly cited.
License URL: https://creativecommons.org/licenses/by/4.0/

Page count: 40

==============================
### Index Participant

**A**

Aaltonen, M.: 2430
Aarts, S.: LB1545-414
Abadir, P.: LB3620-364
Abbate, L. M: 630
Abbott, K. M: 690, LB1545-397
Abdel-Mageed, A.: LB1545-377
Abdelmohsen, K.: LB935-347
Abdul-Malak, Y.: 3245
Abe, T.: 3340-204
Abedsaeedi, Z.: LB1545-435
Abell, J. G: 730, 1045
Abrahamson, K.: 1400-265, 3355-254
Abramenaka-Lachheb, V.: 3375-306
Abrams, L. R: 915-286
Abramson, T. A: 900-249, 2430-302
Abujarad, F.: 3305-109
Abushakrah, J.: 1285
Accius, J. C: 3450
Acevedo, A.: LB3620-379
Achterberg, W.: 4135
Acierno, R.: 1055, 2225, 2365-107
Acker, L.: 2405-231
Acton, G.: 1395-247, 2395-208
Adamek, M. E: 880-200, 905-251, 910-273
Adams, C. M: 815-15
Adams, I.: 845-117
Adams, J.: 2325-26
Adamson, E. M: 3460
Adamson, J.: 2395-213
Adams-Price, C. E: 915-291, 2200
Addison, O.: 630, 3020
Adelnia, F.: 820-24
Adeniji, D. O: 2405-240, 2430-299
Adusei-Asante, K.: 2430-295
Afari, N.: 4080
Affuso, O.: 2410-260
Afolabi, M.: 640, 1355-149
Afunugo, W. E: LB2570-332
Ågård, P.: 1210
Agarwal, M.: 2395-217
Aggarwal, A.: LB1545-366
Agmon, M.: 2315-7
Agmon, M.: LB1545-375
Agniel, D.: 2250
Agree, E. M: 880-202
Aguila, E.: 750
Aguilar, I.: 3070, LB3620-444
Aguilar, S.: 3535
Aguinaga, S.: 3360-266
Aguirre, A.: 850-134
Aguirre, A.: 895-230
Ahamed, S. I: LB3620-327
Ahmadi, S.: LB2570-387
Ahmed, B.: 1325-74
Ahmed, F.: LB2570-423
Ahn, H.: 1402-313
Ahn, S.: LB1545-411
Ahuja, R.: 2325-23
Aiken Morgan, A. T: 1485
Ailshire, J. A: 1315-53, 1345-106, 1401-289, 3275-17, 4000, 4080, LB2570-436
Aizenstein, H.: 825-45
Aizenstein, H.: 2360-94
Ajrouch, K.: 1210, 2455
Akhtar, B.: LB935-381
Akintomide, A.: 3300-100
Akinyemi, A.: 1355-149
Akishita, M.: 820-27, 3325-157
Akosionu, O. C: 2390-200, 3345-217, 3585
Akushevich, I.: 825-60
Akushevich, I.: 2240, 2370-132
Al- heizan, M. O: 2385-187, 2400-226
Al Snih, S.: 660, 850-135, 2385-171, 4020, LB1545-349
Aladeokin, T.: 1315-56
Alahmari, S.: 1310-39
Alain, G.: LB1545-336
Alarcon Garavito, G.: 3355-254
Alarie, M.: 3025
Albert, S.: LB1545-432
Albrecht, J.: 2370-130
Albright, A.: LB1545-332, LB2570-418
Albright, T.: 500
Alconada-Romero, A.: 3345-216
Aldwin, C. M: 3340-209, 3370-301
Alemi, Q.: LB935-363
Alessi, C.: 2395-215, 2420-273
Alexander, E.: 2390-196
Alexander, K.: LB3620-351
Alexander, K.: 4110
Alexander, N.: 530
Alfred, D. L: 2270
AlHasan, D. M: LB1545-364
Ali, I.: 2360-83
Ali, S.: 910-273
Ali, T.: 925-337
Aliyu, K.: 1355-149
Allaire, J. C: 3290-53
Allen, J. R: 920-310
Allen, J.: 1310-36
Allen, N.: 1315-48
Allen, N.: 3280-34
Allen, P. D: 815-12, 855-146
Allen, R. S: 535, 870-183, 1400-264, 2130, 2405-231, 3290-60, LB935-405, LB2570-418
Allen, S.: 3435
Allgood, K.: 3365-280
Allison, D.: LB2570-394
Allison, T. A: 3470
Allore, H.: 1395-248, 3365-278, 3455, LB1545-356
Allred, C. D: 820-32
Allred, K. F: 820-32
Allsopp, R.: 830-72
Almack, K.: 3225
Almardawi, R.: LB1545-424
Almeida, B.: 695
Almeida, D. M: 825-53
Almeida, D. M: 2375-148
Almeida, D. M: 3275-21
Almeida, D. M: 3295-69
Almeida-Meza, P.: 3210
Almen, R.: LB935-404
Aloiau, A.: 840-105
Alpaugh, W.: 840-112
Alston, R.: 2360-100
Alvarez, C.: 2325-28
Alves, S.: 2365-113
Alwerdt, J.: LB3620-347
Amano, H.: 3340-204
Amano, T.: 845-118, 1300-8
Ambale-Venkatesh, B.: 820-34
Ameer, F.: LB3620-377
Ames, A.: 1345-110, 2355-73
Amireault, S.: 2410-255, 3350-238
Amirehsani, K.: LB3620-421
Amjad, H.: 1170, 3420
Amorim, C. F: 2360-87
Amparan, G.: LB935-437
Amspoker, A.: LB2570-390
An, P.: 2370-125, 3170
An, Q.: 500
An, R.: 2360-100
An, S.: 2375-138
An, Y.: 2370-129
Anand, C.: LB935-366, LB3620-326
Anand, P.: 1515
Andel, R.: 590, 1365-177, 1380-196, 3290-59
Andersen, A.: LB2570-339
Andersen, O.: LB2570-339
Andersen, S. L: 2060, 2345-55, LB1545-428, LB2570-343, LB3620-337
Andersen, T.: LB2570-416
Anderson, J. G: 895-238

Anderson, J. A: 2340-47
Anderson, K. K: 2385-190
Anderson, K. A: 885-207, 1385-214
Anderson, L.: 860-161
Anderson, M.: LB2570-368
Anderson, R.: 765
Anderson, R. A: LB1545-425
Andersson, M. A: 885-209
Andreoletti, C.: 2435-307, 2560
Andrew, M.: 565
Andrew, N.: 2390-195
Andrews, E.: 3355-247
Andrews, H.: 3325-171
Andrews, M.: LB1545-441
Andrews, R. M: 3350-245
Anetzberger, G.: 1055, 1265, 2365-107, 3025
Ang, N.: 1365-166
Ang, S.: 2310
Angel, J. L: 750, 1025
Angel, N.: 920-307
Angel, R. J: 750
Angell, B.: 910-274
Anglem, J.: 640
Annoni, G.: 820-31
Ansello, E. F: 3465, 4075
Anthony, D.: 880-197
Antolini, L.: 820-31
Antoniades, J.: 2325-26
Antonucci, T. C: 800, 925-337, 1130, 1195, 1350-121, 2455
Anunsen, R. A: 1285
Anzuoni, K.: 3355-256
Apchemengich, I.: 2410-258
Apenteng, B.: LB1545-391
Apostolides, C.: 3355-248
Applebaum, R. A: 1220, 3555
Apracio-Ugarriza, R.: LB1545-361
Apriceno, M. A: 3370-295
Aranda, M. P: 1355-138, 3070, 3180, 3320-138, 4000, LB3620-444
Arandia, G.: LB935-431
Aransiola, J. O: 890-220, 1355-149, 3300-100
Araujo, L.: 880-201
Arbeev, K.: 825-60, LB1545-440
Arbisi, P.: 1390-236
Arbogast, A.: 3305-110
Archibald, P.: 3360-260
Archuleta, W. P: 2425-290
Ardelt, M.: 2200, 2565
Arendacs, R.: 2315-3
Arevalo-Flechas, L. C: LB1545-382
Argueta, I.: 1402-295
Argueta, J.: 1402-295
Arias Merino, E.: 3290-67
Arias Merino, E.: 3375-302
Arieli, R.: 925-322
Arif, A.: 1345-117
Arling, G.: 3435
Arms, T.: 3430
Armstrong, J.: 565
Armstrong, N.: LB1545-410
Arnold, A.: 3595
Arora, K.: 1100
Arora, N. K: 3140, LB935-416
Arora, R. C: LB2570-329
Arránz Becker, O.: 3200
Arshad, M.: LB3620-326
Arvan, T.: 820-28
Arvanitis, M.: 3595
Aryan Nagabooshanam, V.: 3295-74
Asamane, E. A: LB935-390
Asamane, E. A: LB935-398
Asante, S.: 1400-250
Asch, S.: 3535
Ascha, M.: 895-227
Asgari, M.: 1195
Ashcraft, A. S: 2395-207
Ashe, M. C: LB2570-438, LB3620-383
Ashida, S.: 3065
Ashihara, T.: 1305-23
Ashley, B. L: LB2570-403
Ashrafi, A.: LB3620-337
Asman, A. R: LB935-376
Aspinall, S.: 3390
Astell, A.: 2325-30
Astuti, R. D: 915-287
Atakere, D.: 2080
Atalla, S.: 3100
Atef, B.: 3280-29
Atkins, J.: 1180
Atkinson, E.: 830-68
Atkinson, T. J: 3290-50
Atlas, L. S: 915-280
Attoh-Mensah, E. K.: 825-49
Auerbach, D. I: 755
Auerbach-Burgoon, J.: LB3620-370
Aufill, J.: 2045
Augsburger, M.: 1330-88
August, K. J: 1315-58
Aunger, J. A: LB935-398
Austad, S.: 2230, LB2570-371
Austen, A.: 905-255
Austin, M.: 1345-107
Austin, M.: 3300-90
Austin, N.: 2360-101
Au-Yeung, W. M: LB2570-357
Avanzino, L.: 1402-304
Avent, E. S: 1355-150, 2375-150
Averett, P.: 1380-191
Aversa, Z.: 835-84
Avila, K.: 2360-101
Avis, N. E: 1455
Awosanya, A.: 2310
Awuviry-Newton, K.: 2410-251
Axonovitz, J.: 3300-101
Aye, M.: 3360-265
Ayernor, K.: 3445
Ayyaswami, V.: 1402-309
Azhar, G.: LB3620-369
Azzis, L.: 3390

### B

B. Ferdows, N.: 2545
Baba, A.: LB1545-429
Babulal, G.: LB1545-399
Baccarelli, A.: 3405
Bacharz, K.: 2375-143, 3320-149
Bachelar-Murphy, M.: 2355-74
Bachner, Y.: 1160
Backhaus, R.: 2020, LB1545-414
Backhouse, D.: 1510
Backman, A.: 635, 3345-220
Backus, D.: 900-245
Bacsu, J. R: 2355-62
Badana, A.: 2270, 2355-69, 2375-141
Bae, J.: 3370-297
Baek, J.: LB3620-335
Baeuerle, E.: 820-39, 825-56
Bagaajav, A.: 3445
Baghban Ferdows, S.: 3395
Bahr, P.: 3305-110
Baidwan, N.: 3295-74
Baier, R.: 705
Baik, D.: 4010
Baik, S.: LB3620-353
Bailey, D. E: LB2570-403
Bailey, J.: 3490
Bailey, P. E: 2365-109
Bain, J. R: LB2570-437
Bair, B.: 3165
Bajracharya, R.: 3355-253
Bakas, T.: 1350-120
Baker, H.: 3275-19
Baker, L. D: 850-132
Baker, M. F: LB2570-379
Baker, S. J: 1385-206
Baker, T. A: 1130, 1390-231, 2080, 2425-283
Bakerjian, D.: 3390
Balaswamy, S.: 1070, 1500
Balbim, G. M: 2155, 3360-266
Baldivieso, V. C: LB3620-338
Baldwin, P.: 890-222
Baldwin, P.: 1075
Bales, C. W: 2070, 2380-167
Balik, D.: LB3620-425
Ball, K.: 1360-156
Ballenger, J. F: 3375-314
Ballesteros, S.: 3370-290
Balmuth, A.: 660, 1375-184
Bamonti, P. M: 1390-221, 2255, 3300-97
Bamonti, P.: LB2570-409
Banaszak-Holl, J.: 3345-213
Bancks, M.: 3280-34
Bandeen-Roche, K.: 820-34, 830-75, 3360-268
Banerjee, K.: 1390-231

Banerjee, S.: 3275-20
Bang, E.: 1400-250
Bangerter, L. R: 880-193
Bangerter, L. R: 2325-25, 3180
Banh, A.: 825-44
Banham, V.: 2430-295
Banihashemi, L.: LB2570-324
Baniya, G.: 4050
Bansal, A.: 2360-83
Barani, E.: 3365-277
Baranowski, T.: LB1545-419
Barber, S.: 3380, LB2570-412
Barbieri, V.: 815-9
Barca, M.: 1330-86
Bardach, S. H: 2270
Bardo, A. R: 1325-73, 1385-215, 1400-259, 2010
Bardutz, H. A: 855-153
Barger, K.: 3280-33
Barha, C.: 715, 920-313, 3075, 3280-36
Barker, M.: LB3620-337
Barker, R. O: 1290, 2395-213
Barker, W.: 2355-66
Barmon, C.: 2435-307
Barnard, R.: LB1545-390
Barnes, L. L: 3480
Barnes, S. L: LB3620-404
Barnes, T.: 1015, LB935-355
Barnett, A. E: 2435-310
Barnett, M. D: 815-15, 2340-44, 2425-288
Baron, A.: 3390
Barooah, A.: 1405-323
Barr, E.: 705
Barr, R. A: 2135, 2290
Barragan, C.: 1420, 4170, LB2570-342
Barre, L. K: LB3620-414
Barrett, A.: 1380-193, 2340-48, 3345-229
Barrett, E.: 920-299
Barrientos, M. E: 900-246
Barry, L. C: 915-295, 3010, 3360-263
Barry, R. A: 870-186, 1365-168
Barsness, S.: 505
Bartholomew, A.: LB1545-336
Bartke, A.: 835-83, 3130
Bashian, H. M: 815-10, 2340-41
Bashore, A.: 835-87
Basic, A.: 2340-33, LB935-367
Baskaran, D.: LB3620-338
Basnet, P.: 2395-208
Basque, S.: 3560
Bass, D.: LB3620-352
Bastarache, E. D: 3340-206
Basu, T.: 2010
Batchelor, F.: 1385-206
Bates, E.: 1402-300
Batey, S.: 1400-267
Batsis, J. A: 2070
Battalio, S.: 855-148
Batty, G.: 730
Batzogs, U.: LB3620-374
Baudouin, C.: 1320-64
Bauers, C.: 2385-173
Baugh, M. E: LB3620-348
Baugh, M.: LB2570-437
Bauman, A.: 855-147
Baumbusch, J.: 3345-212, 3435
Baumel, Z. J: 915-284
Bauschpies, W.: LB3620-343
Bay, A.: LB3620-336
Bayliss, E.: 1401-291
Bayly, M.: 3415
Bazzano, L.: 1330-87
Beach, S. R: 620, 1055
Beadle, J.: 3270-1
Beam, C.: 925-317
Beamer, B.: LB1545-424
Bean, J.: 2255, LB1545-363, LB2570-409
Beasley, J.: 1205
Beattie, E.: 505, 850-130
Beattie, Z.: 3535, LB2570-357
Beatty Moody, D. L: 545
Beaulieu, C.: 915-288, 1380-201, LB2570-413
Beauregard, L.: 910-261, 3555
Beavers, D. P: 3325-175
Beavers, K.: 3325-175
Bechshoeft, R.: 820-30
Beck, M. S: 2380-162
Beck, S.: 1115
Becker, C. B: LB2570-399
Becker, H.: 1395-247
Becker, M.: LB3620-344
Becker, T. D: LB935-385
Beckert, T.: 3340-201
Beckett, M.: 520, 2250
Beckham, J.: 1330-83
Beckmann, A.: LB935-346
Beckmann, G.: LB935-381
Bédard, M.: 1410-331, 2550, 3595
Bednarcyzk, M.: 635
Beeber, A.: 3345-224
Beeler, D. M: 3275-14
Beer, J. M: 1401-287, LB3620-329
Beer, J.: 1350-132
Behar, R.: 2355-66
Behrens, E. A: 3365-279
Behrens, L.: LB1545-397
Bekelman, D.: 1401-291
Belanger, E.: 880-194
Beld, M.: LB3620-419
Beleno, R.: 2325-31
Belfer, G.: 885-204
Belfort, T.: 850-141
Bell, D.: 2430-303, LB3620-320
Bell, G.: 870-183
Bell, J.: 2295
Bell, R. K: LB3620-380
Bell, T.: 1175
Bell, T. R: 1360-156
Bell, V.: 1401-273
Bellander, T.: 850-137
Bellingtier, J. A: 3380, LB935-370
Bellon, F.: 3345-216
Belsky, D.: 820-41
Belsky, D. W: 1430
Belsky, D.: 2240, 2370-117
Beltran, S. J: LB2570-400
Belza, B.: 1405-320
Benavidez, G.: 2410-248
Benayoun, B. A: 3540, LB935-351
Bendayan, R.: 1395-244, 3350-244
Bender, A. A: 885-211
Bender, A. A: 2305, 3270-9, LB3620-321
Bender, F.: 910-269, 1320-62
Bene, M. R: 830-77
Bengtson, V.: 1475
Bennett, C.: 920-307, 3495
Bennett, D. A: 3480
Bennett, H. Q: LB2570-319
Bennett, H.: LB3620-416
Bennett, J.: 930-343
Bennett, K.: 3295-72
Bennett, R.: 915-298
Benson, J.: 3410
Benson, W. F: 1505
Benton, D.: 1355-150, 1405-319, 2375-150
Benton, M. J: 840-105, 3325-162
Berg, C.: 1315-48, 3190
Bergeman, C.: 3030, 3560
Bergen, A. W: 3325-152
Bergen, G.: 2555
Bergeron, C. D: 1300-5, 1380-190
Bergh, S.: 1330-86
Bergquist, S. H: 2385-176
Bergren, S. M: LB2570-428
Berg-Weger, M.: 1020, 1385-214, 1530
Berish, D.: 850-133, 3010
Berkman, C.: 3225
Berkman, L. F: 4110
Berkowsky, R. W: 1401-275
Berlin, M.: 3270-8
Bernacki, R. E: 870-181
Bernard, M. A: 2135, 2290
Bern-Klug, M.: 1480, LB2570-430
Berrett-Abebe, J.: 755
Berridge, C.: 1525
Berta, W.: LB3620-391
Bertolotti, M.: 515
Bertoni, A. M: 3590
Bertrand, K.: 2345-55
Bertrand, R. M: 2395-212
Best, C.: 2355-63
Best, J. R: 565, 715, 1510, 2360-97
Best, R. D: LB935-386
Best, R.: 3350-237
Bettega, K.: 3390
Betz, J.: 2495
Bewernitz, M. R: 890-217
Beyth, R. J: 3330-184

Bhalla, N. S: 3325-176
Bhargava, S.: 1175
Bhatta, J.: 1385-212, 2415-266, 3370-292
Bhatta, T.: 895-239, 1400-258
Bhattarai, R.: 3325-176
Bhaumik, A.: 500
Bherer, L.: 3280-29
Bhutkar, A.: 835-92
Bibbo, J.: 1090
Bickhart, S.: 3290-63
Biderman, A.: 1160
Bielak, A. A: 3350-245, 3560
Bienvenu, B.: 610
Biery, N.: 2380-156
Bigonnesse, C.: LB1545-441
Bihary, J. G: 2340-33, LB935-367
Bildtgard, T.: 3200, 3315-124
Binder, E.: 2210
Binette, J. V: 1465
Birchfield, T.: 3320-136
Birditt, K. S: 3425
Birdsall, J.: LB935-405
Birdsong, D.: 2565
Birg, J.: 815-9
Biritwum, R.: LB1545-342
Birkeland, R.: 1385-210, 2375-140
Birtley, N.: LB2570-334
Bischoff-Ferrari, H. A: LB2570-367
Bisconti, T. L: 1380-200, LB3620-406
Bishop, A. J: 645, 1385-212, 1401-285, 1475, 2415-266, 3320-145, 3370-292
Bishop, C. E: 2480
Bishop, N. J: 3295-83
Bishop, T.: LB3620-351
Biss, R.: 2360-98
Bisset, E.: 820-36
Bisson, A. N: 2410-257, 3580
Biswal, S.: 2315-4
Bittinger, K.: 3305-103
Bitto, A.: 2090
Bjarnadottir, R.: 1402-294, 2370-120
Black, J.: 3420
Black, K.: 595
Black, M.: 1305-24
Black, S.: 2405-231
Blackmar, A.: 3275-15
Blackwell, T.: 820-22
Blackwood, J.: 1360-161
Blackwood, K. L: 3330-178
Blair, E.: 2385-183
Blair, J.: 2430-298
Blancato, R.: 760, 1230, 3515
Blanchard, J.: 890-213
Blanco, E.: 2375-153
Blanco-Blanco, J.: 3345-216
Blaskewicz Boron, J.: 3270-1, 3290-47, 3295-80, 3350-234
Blaxton, J.: 3030
Bleijenberg, N.: 675
Bleijlevens, M.: 2380-164
Blieszner, R.: 1025, 1315-51
Blinka, M. D: 4165
Bliwise, D.: 1510
Block, C.: 845-117, 3350-239
Block, L.: 3350-240
Block, P.: LB3620-351
Blocker, K. A: LB1545-373
Blodgett, J. M: 3170, 3325-161
Bloemen, E.: 2370-118
Blomberg, B. A: LB935-384
Blomberg, R.: 3305-102
Blondet, R.: 2495
Bloom-Charette, L.: 3125
Blozis, S.: 920-314
Blue, B.: 830-81
Bluethmann, S. M: 2050
Boateng, A.: 915-289
Bobitt, J.: 1100
Boccanfuso, L.: LB3620-329
Bock, J.: 1390-223
Boddu, P.: 1402-310
Bodenheimer, L.: 885-204
Boerner, K.: 2235, 2340-37, 3315-123
Bogosian, C.: 695
Bohn, L.: 1360-162
Bohnen, N.: 820-38
Boker, S.: 2005
Boks, M. P: LB1545-422
Boles, A.: LB935-431
Bolkan, C.: 1305-26, 2035, 2355-80, 2385-189
Bollinger, S.: 3390
Bolllag, W.: 830-73
Bolognesi, M.: 820-31
Bolstad, C. J: 670, 915-291
Boltz, M.: 1255, 2215, 2315-3, LB2570-358
Bombelli, S.: 820-31
Bond, M.: 3510
Bond, M.: LB1545-395
Bond, M.: LB3620-322
Bonds, K.: LB2570-359
Bonilla, H. J: 820-25
Bonkowski, M. S: LB2570-323
Bonner, A.: 795
Bonner, T.: 1490
Bonner, T.: 2405-234
Bonner Cozad, A.: 1465
Boockvar, K.: 4045
Booker, S.: 775, 2080
Bookheimer, S.: LB2570-335
Bookwala, J.: 1140
Boolani, A.: 1380-190
Boone, A.: 2315-8
Boot, W. R: 620, 1060, 1270, 1525, 2110, LB1545-385
Boot, W.: 2060, 2265, 2475
Booth, K.: 2315-4
Booth, S. L: 715, 3280-33
Booth-Lord, C.: 875-187, 3510
Boparai, M.: 635
Borders, J.: LB3620-421
Borges-Machado, F.: LB2570-341
Borromeo, G. L: 2380-159
Borza, T.: 1330-86
Boscardin, J.: 2315-1
Bosco, E.: 4130
Bose-Brill, S.: LB2570-345
Boss, L. L: 3270-2
Boston, N. C: 915-298
Bostrom, A.: 1405-320
Bostrom, M.: 1365-179
Bosworth, H.: 1330-83
Botor, N.: 730
Botoseneanu, A.: 1395-248, 2370-124
Bott, N.: 1402-300
Boubekri, M.: 3295-84
Bouchard, L. M: 1315-44, 3370-285
Boucher, N.: 520
Boucher, N. A: 3320-151
Boudreau, R. M: 565, 1425, 2370-125, 3275-24, LB935-368, LB1545-428, LB2570-343
Bouldin, E. D: 920-304, 1030, 2555, 4110
Boutin, R. D: LB1545-390
Bouweraerts, A. G: 2360-91
Bowblis, J. R: 2400-220, 2480, 3555
Bowblis, J. R: 3555
Bowen, P.: 2410-260
Bower, E. S: 910-275, 4140
Bower, K. L: LB935-424
Bowers, B.: 1155, 2110, 2525
Bowland, S.: 1265, 4060
Bowles, K. H: 1170
Bowling, B.: 4145
Bownes, E.: 640
Boxer, R. S: 1401-291, 2385-180, 3280-35
Boyd, K.: 4160
Boyd, M.: LB2570-425
Boyken, L.: 880-189
Bozzo, D.: LB935-427
Brach, J.: LB1545-363
Bradley, E. S: 820-29
Brady, S.: 660, 1375-184
Bragg, R. T: 835-100
Braithwaite, S.: 825-43
Braley, M.: 820-28, 3325-172
Branco, K. J: 1300-9
Brandhorst, S.: 1310-43
Brandt, C.: 2370-119
Brangman, S. A: 4110
Branson, S.: 1090
Bratzke, L.: 2315-2
Braun, K.: LB3620-374
Brauner, D.: 910-258
Bravo, V.: 1365-171
Brayne, C.: LB1545-333
Breakwell, S.: LB3620-404
Breckman, R.: 2225
Breda, K.: 3420
Brem, R.: 765

Brennan-Ing, M.: 2485, 3525
Brennan-Taylor, M.: 3330-187
Brettschneider, C.: 3320-139
Breunig, I.: 3330-179
Brewster, G.: 1355-142
Brewster, G. S: 1510
Brewster, G.: 2375-149, 3100
Brewster, K. K: 2495
Breytspraak, L.: 2340-36
Brick, T.: 2005
Brickman, A.: 2495
Brickman, S.: LB935-399
Bridges, K.: 680, 1465
Briganti, J.: 3290-62
Bright, B.: LB935-437
Bright, C. L: 3330-188
Brijnath, B.: 2325-26
Briley, M.: 2360-86, LB935-441
Brill, S.: 710
Bristol, A. A: 870-184, LB935-401, LB1545-335
Bristowe, K.: 3225
Brittain, K.: 1290, 4055
Broccoli, J.: 1400-251, 3600
Brodaty, H.: 655
Brody, A. A: 870-184, 1495, 2395-215, LB935-401, LB1545-335
Broese van Groenou, M.: 2430-292
Bromet, E.: 825-52
Bronskill, S. E: 555
Brooks, D.: LB3620-405
Brooks, M.: 3300-94, 3340-205
Brooks-Wilson, A.: LB2570-354
Broome, A.: LB1545-352
Brorsson, A.: 3565
Brossoie, N.: 3345-223
Brothers, A. F: 2040
Brotherson, S.: LB3620-441
Brown, C.: 1485, 3095
Brown, C. H: 2360-92
Brown, C. H: LB1545-371
Brown, C. J: 1400-255
Brown, C. J: 1425, 3365-274
Brown, D.: LB3620-423
Brown, E.: LB2570-384
Brown, J.: 805, 4150
Brown, J.: LB2570-394
Brown, J.: 865-168
Brown, J. A: 1270
Brown, L.: 1050
Brown, L.: 2390-202
Brown, L. M: 2065, 2450, 3045
Brown, M. T: 4110
Brown, M. J: 2485
Brown, P. P: 1485
Brown, R.: 1305-33
Brown, R. P: 1390-223
Brown, S.: 1365-166
Brown, S. L: 4065
Brown-Hughes, T.: 3355-247
Browning, C.: 2325-26
Broyles, I. H: 2385-190
Brozgol, M.: 4020
Bruce, M. A: 1145, 3360-260
Brummett, C.: 1425
Brunet, A.: 1245
Bruns, D.: LB1545-402
Bruns, D.: LB2570-346
Brunson, K.: 1380-192
Brush, D.: LB1545-353
Brush, S.: LB2570-368
Brustio, P. R: 3325-159
Bryan, C.: 1355-147
Bryant, J.: LB3620-405
Brydges, C. R: 3350-245
Brydges, C.: 3560, LB935-397
Bryson, W.: LB935-419
Buchman, A.: 1402-303
Buck, H. G: 1330-93
Buckwalter, K. C: 3550
Bueno, T.: 1390-222
Buford, T. W: 4175, LB935-371
Bugos, J.: LB3620-327
Bui, C. N: 915-288, 1380-201, 2385-179, LB935-365
Bull, S.: 1401-291
Bullock, L.: LB1545-438
Bulow, J.: 820-30
Bültmann, U.: 660
Bunger, A.: 3520
Burack, O. R: LB3620-370
Burack, O. R: LB3620-393
Burant, C. J: 890-216, 915-293, 1320-63
Burchett, C. O: LB935-444
Burgdorf, J.: 1110, 1170, 2045, 2270, 3420
Burgess, E. O: 920-312, LB3620-321
Burgunder, A.: 2155
Burhenn, P.: 855-146
Burholt, V.: 2105
Burke, R.: 3390
Burke, S. L: 1390-237, 2355-66, 3275-23
Burkhardt, J.: LB1545-332
Burnaevskiy, N.: 830-78
Burnes, D.: 2225, 2365-108, LB935-444
Burnett, J.: 2365-114
Burnette, D.: 885-212, 2450, 3070, 3180
Burnight, C.: LB3620-425
Burnight, K.: LB3620-425
Burning, M.: 830-68
Burns, E. R: 2555, LB935-425
Burns, J.: 3175
Burns, V. F: 2540, LB1545-439
Burnside, L.: LB3620-344
Burr, J. A: 3270-10
Burr, J.: 915-288, 1065, 1095, 1360-163, 3315-129
Burrows, D.: LB3620-385
Burston, B.: 3330-191
Burzynska, A. Z: 590
Busby-Whitehead, J.: LB935-392
Butler, A.: LB935-377
Butler, J.: LB2570-416
Butler, S. S: 910-257, 3345-233, LB935-434
Buttelmann, F.: LB935-370
Buurman, B.: 1365-176
Buxton, O. M: 3275-21, 3580
Buys, D. R: 1425, 3365-274
Buys, K.: 3365-274
Bybee, S.: 500
Byers, A.: 1390-235
Bygrave, D.: 655
Byles, J.: 2410-251
Byrd, D. R: 2455, 3290-53
Byrne, K.: 3330-177

### C

Cabin, W. D: 910-256, 1375-189, 1400-269
Cabrera, L. Y: 2455
Cabrero Castro, E.: 2360-104
Cacchione, P. Z: LB3620-340
Cacciottolo, M.: 825-50
Caceres, N. A: LB935-363
Cadar, D.: 1045, 3210
Cadet, T. J: 1390-237, 2385-174, 3275-22
Cadogan, M.: 2395-215
Caffeé, L.: 2420-276
Caffrey, C.: 910-268
Caffrey, C.: 2390-203, 2395-214
Cagle, J.: LB2570-434, LB3620-355
Cahill, L.: 3325-165
Cai, B.: LB1545-364
Cai, S.: LB3620-386
Cai, X.: LB1545-381
Cai, Y.: 3220
Cajita, M.: 2355-79
Calamia, M. R: 1345-116
Caldera, M.: LB2570-335
Caldera, S.: 910-259
Calderon-Larranaga, A.: 3170, 3325-173, 3420
Caldwell, S.: LB1545-357
Callahan, B. L: 3295-87
Callahan, K. E: 4045
Calvillo, M.: 825-61
Calvo, R.: 2280
Camacho, D.: 3070, 3180
Camell, C.: 835-96
Cameron, K. A: 3415, 4030
Camp, L.: 1401-271
Campbel, T.: 1400-265
Campbell, L.: LB2570-335
Campbell, M.: 1315-48
Campbell, m.: 825-43
Campbell, R. J: 860-157, 2365-112
Campione, J. R: 2045
Campitelli, M. A: 555
Canamucio, A.: 3390
Canell, A. E: 815-10, LB3620-346
Canham, S.: LB935-419
Canham, S. L: LB1545-439

Cannell, M. B: 930-341
Canning, D.: 1400-261
Cannizzaro, K. A: 2385-173
Cannon, J.: LB3620-404
Cannon, M. L: 810-2, 2450
Cantave Burton, C.: 1465
Cantu, P. A: 3360-259
Cao, G.: 1330-77, 2370-137, 4005
Cao, J.: 1305-21
Cao, W.: 825-47, LB935-364
Cao, X.: LB1545-365
Caouette, S.: 3270-2
Capistrant, B. D: 2380-155
Caprio, T. V: 1530, LB1545-380
Carberry, K.: 850-134
Cardenas, V.: LB1545-443
Carder, P.: 1305-27, 2305, 3345-224, LB3620-390, 2470
Carlson, B. W: LB3620-358
Carlson, M. C: 830-75, 2095, 2170, 3350-245, 4025
Carlson, M.: 890-213
Carmel, S.: 1160
Carmichael, A. G: LB2570-435, LB3620-378
Carpenter, B.: 535, 3270-6, 3310-119, LB935-382
Carpenter, J. G: 605
Carr, D. C: 1160, 1400-252, 2550
Carr, D.: 1065, 1190, 1300-3, 1440, 2205, 3150, 3315-125, LB2570-374
Carr, E.: 3350-244
Carr, K. A: LB935-389
Carramusa, C.: 1360-153
Carrasquillo, O.: 1402-301
Carreno, J.: 1402-312
Carrero-Tagle, M.: 2430-300
Carrico, C.: 870-186, 1365-168, 1530
Carrion, I. V: 3070
Carroll, D.: LB2570-418
Carstensen, L.: 865-165, 925-323
Carter, C. S: 4175
Carter, C.: LB935-380
Carter, M. W: 2370-121, 2390-198
Carvalho, J.: LB2570-341
Casaletto, K.: 3205, LB1545-410
Casanova, G.: 2425-281
Casanova, R.: 1402-296
Case, B.: 4005
Casey, A. S: 655
Casey, D.: 920-299
Cashdan, E.: 2325-24
Casillas, A.: 1401-291
Caskie, G.: 815-10, 1380-199, 2340-41, LB3620-346
Caspi, A.: LB1545-347
Cassel, S.: 3375-308
Castellon-Lopez, Y. M: LB3620-408
Castle, N.: 3345-222, 3435, 4050
Castle, S.: 530, 920-303
Cater, D.: LB2570-404
Catic, A.: 850-145, 3560
Catlin, C.: 1390-221
Cattoretti, G.: 820-31
Caudle, B.: 850-132
Cauley, J. A: 820-22, LB935-368
CAVALCANTI, F.: 2360-85
Cavaleiro, A.: 2325-28
Cavanaugh, J.: LB3620-399
Caves, K.: 530, 1402-308
Cawthon, P. M: 820-22
Cena, C.: 3325-158
Cenko, E.: LB3620-403
Cereatti, A.: 1402-304
Cerino, E. S: 1195
Cerino, E. S: LB3620-382
Cermakova, P.: LB1545-333
Chacko, S.: 1310-37
Chacko, T.: LB3620-339
Chae, J.: LB935-351
Chahal, J. K: 505, 3375-303
Chai, H.: 1360-159, 3340-208
Chai, H.: LB2570-362
Chai, Y.: 3075
Chamberlain, S.: 2430-293
Champion, J. D: 2395-208
Chan, A. T: 3260
Chan, A.: 715, 865-167, 2360-96
Chan, A.: 3245
Chan, B.: 3245
Chan, J. L: 715
Chan, L.: 1515
Chan, P.: 1510, 2360-97
Chan, R.: 915-294
Chan, S.: 895-233, 1330-85
Chan, T.: 1402-295
Chang, C.: LB3620-343
Chang, E.: 815-17, 2225
Chang, E.: 1170
Chang, M.: 2370-131
Chang, S.: 1375-186
Chang, S.: 1365-173
Chang, Y.: 2375-146
Chang, Y.: 920-312
CHANG, Y.: 895-234
Chang, Y.: 1330-90
Chanti-Ketterl, M.: LB2570-322
Chao, N. J: 2345-56
Chao, Y.: 920-312
Chao, Y.: 1330-90, 3505, 4105
Chaparro, M.: 895-228
Chapman, A.: 1055
Chapman, S. A: 3555
Chappell, A.: 1385-203
Chard, S.: 3330-188
Charlton, J.: 1410-331
Charness, N. H: 620, 1130
Charness, N.: 1270
Charness, N. H: LB1545-385
Chase, J. D: 1170, LB1545-338
Chase, J.: 920-300
Chasteen, A. L: 815-5
Chau, A.: 2425-286
Chaudhari, N.: 825-61
Chaudhury, H.: 1401-292
Chaudhury, H.: 3565
Chaves, P. H: 3325-153
Chavoix, C.: 825-49
Che, Y.: LB3620-377
Chee, K.: LB1545-352
Cheesman, K. L: 2415-265
Chen, A.: LB3620-427
Chen, A. T: LB935-445, LB1545-358
Chen, C.: 1297, LB3620-319
Chen, C.: 1405-319
Chen, C.: LB935-354
Chen, D.: 1300-7, LB2570-381
Chen, E. K: 2235, 3310-113
Chen, F.: 3455
Chen, G.: 2375-152
Chen, H.: 3285-37
chen, h.: 805
Chen, H.: 3145
Chen, H.: LB2570-344
Chen, J.: 615
Chen, J.: LB2570-441
Chen, L.: 2400-224
Chen, L.: 1340-102, 3345-231, 4160
Chen, L.: LB935-415
Chen, M.: 920-312, 4005
Chen, M.: 825-42
Chen, N.: 2360-94
Chen, P.: 900-246
Chen, P.: 1320-63
Chen, P.: 3220
Chen, R.: 830-72
Chen, S.: 1405-322
Chen, X.: 1400-262
Chen, X.: 885-210
Chen, Y.: 910-267, 1300-7, 2385-182, LB2570-381
Chen, Y. T: 3335-196
Chen, Y.: 2030, 3395
Chen, Z.: 2030
Chen, Z.: 1330-77, 2370-137
Chen, Z.: 4005
Cheng, G.: 865-167
Cheng, J.: 855-151
Cheng, J.: 895-230
Cheng, K. G: 2375-145
Cheng, M.: 880-199
Cheng, S.: 4080
Cheng, S.: 845-120, 3320-147, LB3620-432
Cheng, Y.: 3230
Cheon, J.: LB2570-434
Cheon, J.: 3375-309
Chernoff, R.: LB935-420
Cherrier, M.: LB1545-412
Cherry, B. J: 3365-272
Cherry, D.: 1355-138

Cherry, K. E: 815-12, 1345-116, 2065, LB1545-417
Chess, E.: 845-116
Chesworth, B.: 3330-177
Cheung, E.: LB2570-407
Cheung, W.: 2360-90
Cheval, B.: 3030
Chhatre, V.: LB1545-402
Chi, C.: LB935-375
Chi, I.: 2475, LB2570-333
Chi, N.: 3365-277
Chia, B.: 885-210
Chia, C.: 835-99
Chiang, A.: 3420
Chiang, T.: 910-267, 1300-7
Chien, S.: 1401-288
Chiles Shaffer, N.: 3095
Chilton, R. L: 920-302
Chinchankar, M. N: 830-71
Chinda, B.: 820-28
Chinda, B.: 3325-172, LB2570-354
Ching, J.: 715
Ching, L.: LB2570-421
Chiodo, L.: 930-342
Chippendale, T.: 855-148
Chiriboga, D.: 1325-70, 2140, 2405-235
Chirinos, C. L: 2355-66
Chisholm, L.: 520
Chit, A.: 4130
Chiu, C.: 805
Chiu, R.: 1345-115
Chiu, T.: 1345-115
Cho, D.: LB935-412
Cho, E.: 840-111, 3295-77, LB1545-360, LB2570-317
Cho, J.: 645, 865-171, 1325-69
Cho, J.: 2300, 3320-136, 3370-293
Cho, J.: 925-332
Cho, S.: 915-282, 1390-227
Cho, S.: 2405-244, 3360-257
Cho, Y.: 1395-241
Choate, S.: 2385-192
Chocano-Bedoya, P. O: LB2570-367
Chocron, S.: 2090
Chodos, A.: 900-246, LB2570-336
Chodosh, J.: 1297, 2395-215
Choi, E.: 850-129, 1401-284
Choi, H.: 3315-121
Choi, J.: 855-151
Choi, J.: 3325-174
Choi, M.: 3520, 4055
Choi, M.: 1525, 2410-252, 2450
Choi, N. G: 1100, 2385-170
Choi, S.: 2355-79
Choi, S.: 865-162
Choi, S.: LB2570-337
Choi, W.: 1395-242
Choi, Y.: 910-279, 3275-17, LB2570-436
Choi, Y.: 2310
Choi-Allum, L.: LB3620-366
Chong, M.: 2360-95
Chopik, W. J: 550, 4100
Chopra, A.: 885-204
Chou, C.: 2395-216
Chou, L.: 850-135, 2385-171, 4020, LB1545-349
Choun, S.: 3340-209, 3370-301
Chow, E. O: LB3620-435
Chow, S. W: 895-231
Choy, J.: 2355-77
Christ, S. L: 1395-246
Christensen, J.: 3070
Christensen, K.: 2370-125, 3170, LB1545-428, LB2570-338
Christian, T. J: 520
Chu, C. H: 2360-98
Chu, F.: LB935-445, LB1545-358
Chu, L.: LB935-361, LB1545-367
Chu, N.: 830-75, 2380-168, 4020
Chui, C.: 1345-115
Chukwurah, Q. C: 895-228
Chukwurah, Q. C: 1330-87
Chun, A.: 895-231
Chun, H.: 2325-22
Chun, S.: LB3620-394
Chung, J.: 1525
Chung, K.: 2385-182
Chung, S.: 2405-235
Chung, T.: LB3620-364
Chunga, R. E: 3110
Chunga, R. E: 3315-125
Church, S.: 1335-97
Churchill, L. C: 895-230
Churchill, R. A: LB935-441
Chusyd, D. E: LB2570-394
Ciancibello, A.: LB3620-362
Cianelli, R.: LB1545-426
Ciarleglio, A.: 2495
Cicala, D.: 1365-166
Cichy, K. E: 4035
Cifuentes, H.: LB935-349
Cil, G.: 3325-152
Cimarolli, V. R: 2015, 2520, LB3620-370
Ciofi, J.: LB3620-321
Ciolino, J.: 4030
Cirillo, N.: 915-298
Civitella, F.: 1402-312
Clare, L.: 650
Clark, B.: 1410-330
Clark, C.: 3330-187
Clark, D.: 820-19
Clark, J.: 1320-62
Clark, K. C: LB1545-438
Clark, K. F: 3375-314
Clark, M. A: 740
Clark, P.: 2220
Clark, P. G: 4075
Clark, S.: 2370-118
Clark, V.: 3535
Clarke, A.: 895-230
Clarke, C.: 2360-100
Clarke, G. D: 835-94
Clarke, M.: 825-53
Clarke, P.: LB2570-384
Clarke, P. J: 600
Clark-Shirley, L. J: 680, 3410
Clauser, S. B: 3140, LB935-416
Clay, O. J: 2410-260
Clayton, Z. S: 835-89
Clegg, A.: 1360-165
Clemency, B. M: 2385-181
Clement, F.: 880-199
Clemson, L.: LB2570-353
Cleveland, M.: 4045
Clevenger, C.: 840-107, 850-125
Clish, C.: 3275-24
Clouston, S.: 825-52
Cobb, R. J: 1050, 1450
Cochran, K.: 2185
Cochrane, B. B: 1455
Coffey, T.: 1380-190
Coggan, A. R: 820-18
Cohen, A. L: LB1545-391
Cohen, A. A: 525, 4005
Cohen, C. C: 2370-135
Cohen, D.: 1360-160
Cohen, H.: 1300-13
Cohen, H. J: 2345-56, 3265, LB1545-347
Cohen, M. A: 865-163, 3015, 3140, 3570, 4050
Cohen, P.: 2510, LB935-359, LB2570-439
Cohen, R.: 710
Cohen, S. A: 1170, 1400-251, 3600
Cohen-Adad, J.: 3280-29
Cohen-Mansfield, J.: 710
Cohn, T.: LB1545-407
Coldiron, A. M: 815-15
Coldiron, A. M: 2340-44
Cole, L.: 850-138
Cole, T. R: 1225
Coleman, C.: LB2570-364
Coleman, M.: 3305-102
Colibaba, A.: 560, LB3620-373
Collaborative, O.: LB3620-368
Collette, B.: LB3620-329
Collins, S.: 910-265
Colman, R.: 4175
Colmenares, C.: 835-91
Colon-Emeric, C.: 3365-275, 4130
Colson, T.: LB3620-439
Cominato, M.: 1515
Commandeur, D. T: 2360-105
Condon, S. E: 1385-217
Cone, N.: 915-296, LB3620-410
Cong, Z.: 2450, 4115
Connell, C. M: 705, 3365-280
Connelly, C.: 915-297
Conner, K.: 3275-22
Conner, N. E: LB935-413
Conrad, K.: LB935-377
Conrad, K.: 3360-269, LB935-377
Conte, F.: 860-158
Contreras, B.: LB935-364

Convery, K.: 870-184
Conwell, Y.: 3360-263, 4140, LB1545-380
Cook, R.: LB1545-402
Cook, W.: 565
Cooke, E.: LB2570-351
Cooke, H. A: 3345-212, 3435
Cooke, V.: 3435
Coolidge, F. L: 915-283
Coon, D.: 2300
Cooney, R. V: LB935-428
Cooney, T. M: LB3620-332
Coons, J.: 3340-203, 4145
Cooper, G.: 1395-243
Cooper, P. S: LB1545-338
Cooper, R.: 3170
Copel, L.: LB1545-369
Coppard, L.: 1345-111
Corazzini, K.: 780, 2020
Corazzini, K.: LB2570-385, LB3620-349
Corbett, D.: 1402-296, 2410-264
Corcos, D.: 4030
Cordes, B.: LB2570-410
Cordova-Marks, F.: LB2570-445
Corey, K. L: 840-109
Corkum, A.: 3295-71
Corley, C.: 4060
Corley, C.: 855-153
Corley, R.: 1150
Corneau, E.: 3435
Cornejo Thumm, P.: 4020
Cornelison, L.: 2515
Cornell, C.: 1365-179
Cornell, P. Y: 1375-187, 2470
Corner, L.: LB2570-319
Cortes, T. A: 1530
Cosentino, S.: 3290-65
Cosentino, S.: LB2570-343, LB3620-337
Costa, E.: 1402-307, 3325-158
Costacou, T.: 565
Costas Muniz, R.: 635, 1365-174, 3275-20, 4110
Costlow, K.: 915-281
Côté, A.: 4005
Cothran, F.: 2295
Cotnam, V.: LB935-394
Cotten, S.: 1060, LB3620-343
Cotton, S. G: 1345-107, 1365-167, 2355-76, 3300-90
Coughlin, J.: 660, 1375-184
Coulourides Kogan, A. M: 520
Coupal, S.: LB3620-414
Coursaris, C.: 3415, 4020
Courser, R.: 3365-280
Cousins, K. A: 850-136
Couture, M.: 2045, 3025
Couve, A.: LB935-372
Covinsky, K.: 1170, 2010, 2315-1, 3420
Cowell, A.: LB935-443
Cowley, A.: 1315-47
Cox, B.: 1385-217, 2415-265
Cox, C.: 800
Cox, D. W: 3310-111
Cox, J.: 640
Coyle, C. E: 910-265, 1445, 2385-179, LB3620-359
Coyle, E.: 920-310
Coyle, M. H: 3270-9
Coyne, S.: 1401-277
Craft, M.: LB3620-358
Cragle, D.: LB3620-341
Crandall, J.: 1402-315
Crawford, B.: LB935-437
Cremer, S.: 2380-164
Crespi, E.: 2355-80
Crimmins, E.: 710, 1315-53, 1400-262, 1401-289, 2260, 2345-57, 3275-17, 4080, LB2570-436
Crist, J. D: 3120
Crittenden, J.: 910-257, 1305-32, LB935-434
Crockett, R. A: 715, 920-313
Crockett, R.: 2215
Crockett, R. A: 2360-97, 3295-72
Croff, R. L: 2405-241
Croker, J.: 1100
Cronkright, A.: LB3620-424
Cross, S. H: 870-182
Crouch, M.: LB1545-348
Crowe, C. L: 3325-171
Crowe, M.: 1360-156, 2410-260, 3595
Crowe-White, K.: 3295-74
Crowley, M.: 1350-129
Crowther, M. R: 640, 2405-231, 2500
Cruitt, P. J: LB935-386
Crumley-Branyon, J.: 815-5
Crutzen, R.: 565
Cruz-Martinez, J.: LB1545-382
Crystal, S.: 910-274
Crystal, S.: 2205
Csiszar, A.: LB3620-358
Cuff, G.: 3360-265
Cui, C.: 835-99
Cullati, S.: 3030
Culley, D. J: LB2570-329
Culminskaya, I.: 820-33
Cummings, G. E: 4130
Cummings, G. G: 2315-15, 2395-219, 4130
Cummings, S. R: 820-22, 830-80
Cummins, P. N: LB935-350
Cummins, P.: 510, 1285, 1325-71, 1400-259, 1401-290, 3300-99, 3305-110
Cunningham, J.: LB2570-445
Curl, A. L: 860-160, LB3620-332
Curran, S. P: 830-64
Curren-Vo, H.: 3345-219
Curtin, C.: 2415-268
Curtin, C.: 3305-109
Curtis, L.: 3595
Curtis, R. G: LB1545-445
Curyto, K.: 3255
Curyto, K.: 3255, LB3620-339
Cushman, M.: 3280-33
Cushman, M.: 4045
Cutchin, M.: 745, 2105, 3565
Cuthbert, K.: LB3620-367
Cvejkus, R. K: 2370-136, 3170
Czaja, S. J: 550, 620, 1055, 1130, 1295, 3315-130
Czarnecki, P.: LB2570-372
Czirr, E.: 825-43

D

Da Silva, J.: 1390-222
Da Silva, R.: 2360-87
Dabelko-Schoeny, H.: 885-207, 1305-28, 3520, 4055
Daddato, A. E: 2385-180, 3280-35
D'Agostino, C.: 825-51
Dahle, C.: LB935-366
Dai, F.: LB935-356
Dakin, E.: 3430
Dale, M.: LB935-392
Dallo, F.: 2355-64, 2455
Dalton, H. M: 830-64
Dalton, J. E: 835-86
Damberg, C.: 2250
D'Ambrosio, J.: 1345-107, 1365-167, 2355-76, 3300-90
D'Ambrosio, L.: 660, 1375-184, 3180
D'Amico, C. O: LB1545-335
D'Amico, D.: 820-20
Damman, M.: 865-170
Dang, S.: 840-108
Dang, S.: 1320-61
Dang, S.: 1330-94, 1402-301
Dang, S.: 2350-59
Dang, S.: 3320-137
Dang, W.: LB1545-365, LB2570-352, LB3620-384
Daniel, K. M: 3510
Daniela, M.: 3320-134
Danielson, M. E: LB1545-432
Danilovich, M.: 880-189
Danilovich, M.: 2385-185
Danilovich, M.: 4030
Dannefer, D.: 2205, 3085
Dannewitz, H.: 2390-202
Dannewitz, H. C: LB1545-385
D'Antonio, P. M: 1230, 2285
Darabidian, B.: 3430
D'Arcy, R.: 3330-189
Darin-Mattsson, A.: 515
Darling, N.: 1350-128

Darvishpoor Kakhki, A.: LB1545-435
Darwiche, J.: 3315-123
Darzins, P.: 1410-331
Das, S.: 1401-271
Daskalopoulou, C.: 4040
Dassel, K. B: 500, 735, LB2570-416
Daum, C. H: 2325-30
Dave, C.: 3050
Dave, P.: 1310-39
Davey, A.: LB3620-389
Davidson, E. J: 670
Davidson, P.: LB1545-435
Davies, L.: LB1545-418
Davila, H.: 910-258, 2390-200, 3345-217
Davila, V. S: 2385-185
Davis, D.: 3170
Davis, D. L: LB1545-424
Davis, E.: LB935-398
Davis, E. M: 2355-67
Davis, J. C: 565, 1510, 2360-90, 3295-72
Davis, J.: 1402-310, 2340-36
Davis, M.: 835-87
Davis, N.: LB2570-330
Davis, T.: 2435-306
Davis-Ajami, M.: 3555
Davitt, J.: 3330-188
Davlyatov, G. K: 910-262, 2395-218, 3345-213, 3435
D'Avolio, D. A: LB935-379
Davy, P.: 830-72
Dawson, N. T: 920-308
D'Cunha, N. M: 705
de Bruin, S.: 635
De Donder, L.: 1305-22
De Gomensoro, E.: 1135
de Jong, L. D: 730
De Lange, F.: 3230
de Man-van Ginkel, J. M: 2380-164
de Medeiros, K.: 2200, LB3620-413
de Paula Couto, C.: 3460
De Vilmorin, C.: 2310
de Vivo, I.: 830-72
de Vries, E.: LB1545-414
de Wit, N.: 675
De Witte, N.: 3270-5
Deal, J. A: 2380-166, 2425-291, 2495, 3075
DeAngelis, T.: LB3620-399
Deardorff, W.: 665
DeCaro, J. A: 3365-279
Deeg, D.: 2430-292
Deer, R. R: LB935-378, LB2570-328
Degenholtz, H.: 690, 1220, 2305, 2480, 3240, LB3620-386
DeGennaro, J.: 885-204
deGrauw, X.: 2385-186
DeGutis, J.: LB1545-363
Deichert, J.: 3345-225
Deimling, G.: 915-293
Deindl, C.: LB2570-363
Dekhtyar, S.: 515, 3420
Del Din, S.: 1402-304
del Rosario, K. E: 2340-45
Del Signore, S.: 1402-298, 3325-159
DeLaTorre, A.: 1305-16
Delbaere, K.: 565
Delbono, O.: 820-25, LB1545-387
Delgado, F.: 890-223, 1095
Deller, J.: 3520
Delmonico, M. J: LB2570-422
DeLouize, A.: LB1545-342
DeLuca, J. A: 820-32
DeMaio, F.: 1300-6
DeMaria, J.: 925-329
Dembo, R.: 2550
Dembosky, J.: 2250
Demiris, G.: 1080, 2310, LB935-400, LB1545-358
DeMott, A.: 3175, 3305-107
Denburg, N.: 3350-239
Denny, P.: 855-150
Denson, K.: LB1545-400
Denson, S.: LB1545-400
Dentino, A.: 1365-171
DePaul, V.: LB3620-368
Depenbrock, A.: 2360-92
DerAnanian, C.: 890-223, 1095
Derby, C.: 845-124, 2445
DeRosa, N.: 1310-38
Desai, L.: 2380-157
Descatoire, P.: 825-49
Despins, L. A: 820-37
DeStefano, C.: LB1545-352
Detwiler, B.: 1380-199
Deutsch, A.: 2385-190
Deutsch, M. B: LB3620-419
Devgan, A.: LB935-359
DeVito, A.: LB3620-318
Dewey, E.: 3325-175
Dhakal, U.: 1305-18
Dharshi, N.: LB1545-439
Dhokai, N.: 610
Di Gessa, G.: 1350-130
Di Stefano, M.: 1410-331
Diallo, H.: LB2570-396
Dian, L.: 565
Dias, C.: 1350-131
Diaz, L.: 880-189, 4030
Diaz Moore, K.: 1305-16
Diaz-Santos, M.: LB2570-335
Diaz-Valdes Iriarte, A. E: 865-174
DiBartolo, M. C: 900-242
DiBartolo, M.: 2360-102
DiBlasio, C.: 2315-14, 3595
Dick, A. W: 2395-217
Dickens, C. N: 4085
Dickens, J.: 3310-117
Dickerson, A. E: 1410-330, 3305-102
Dicocco, C.: 810-3
Dieckmann, N.: 840-104, 1360-160
Diederich, F.: 3320-139
Diehl, M.: 3380
Dieker, J.: 2375-143
DiGasbarro, D.: 845-119
DiGuiseppi, C.: 3325-171
Dijkstra, N. E: 675
Dillon, S.: 825-48, 4130
DiMaria-Ghalili, R.: 2070
Diminich, E. D: 825-52
Dindo, L.: 850-145, 3560
Ding, K.: 3305-108, LB2570-407
Ding, K.: 830-73
DiNitto, D. M: 2385-170
Disco, M.: 1305-24
Dixit, V.: 835-96
Dixon, M.: 2310
Dixon, R.: 1360-162
Djukaric, T.: LB1545-334
Do, H.: 1401-285
Doan, L.: 2405-237, 3370-300
Doan, W.: 3295-89
Dobbins, S.: 1330-91
Dobbs, D. J: 1260, 1405-325, 2065, 2435-311, 3345-224, LB3620-327
Dobiszewski, S.: LB2570-422
Dobson, R.: 1395-244, 3350-244
Docherty, S. L: LB1545-425
Documet, P. I: 3415
Dodge, H. H: 1195, 3280-30
Doh, D.: 2430-295
Doherty, K.: LB2570-409
Dolansky, M. A: 895-240
Dolbin-MacNab, M. L: 1350-129, 3025
Doll, G.: 2515
Doll, J.: 860-158
Domingue, B.: 1430
Donaldson, G.: LB935-400
Donaldson-Davis, K.: 3045
Donelan, K.: 755
Dong, C.: 1402-301
Dong, G.: LB2570-428, LB3620-430
Dong, J.: LB1545-387
Dong, T.: 1340-103
Dong, X.: 615, 1330-90, 2010, 3505, 4105, LB2570-405, LB3620-428
Donini, L. M: 1402-298, 3325-159
Donnellan, K. M: 1505
Donnelly, C.: LB3620-368
Donnelly, R.: 2280, 3150
Donnenfeld, E.: 1320-64
Donohue, J. M: 565
Donorfio, L. K: 900-248, 2560
Donovan, E.: 2420-272
Dore, E. C: 3340-207
Dorigatti, J.: 820-35, 830-77
Dorman, H.: 535, 1400-264
Doron, I.: 1055
Dorr, D. A: 1395-248
Dorsey, S.: 2385-186
Dosa, D.: 1405-325, 3345-215

Doshi, S.: 2410-261
Dotan, E.: 855-146
Douglas, E.: 2430-303, LB3620-320
Douglas, J. W: 2390-201, 2395-211, 3300-98, 3345-228
Douglas, R. A: 2340-48
Douglas, S.: 710
Dounis, G.: LB1545-391
Dourado, M.: 850-141
Dow, B.: 915-290
Dowling, M.: 850-144
Downer, B.: 850-135, 1360-158, 2545, 4135, LB1545-427
Downer, L. B: LB1545-345
Downey, L. A: 3295-81
Doyle, P.: LB2570-351, LB3620-321
Dozier, M. E: 670
Dragan, D.: 2130, LB2570-418
Drahota, A. K: 2380-161
Drake, C.: 2385-180
Drewelies, J.: 3190
Drickamer, M.: LB935-384
Driessnack, M.: LB2570-359
Driscoll, L.: 915-288
Driscoll, R.: 835-99
Drobot, A. C: 2390-202
Drummond, N.: 850-126
Drury, T.: 3275-21
D'Silva, J.: 880-194
D'Souza, N.: 1402-312
Du, P.: 4050
Du, P.: 545
Du, S.: LB935-373
Du, X.: 2385-192
Du, Y.: 2165
Du Bois, A.: 920-303
Duan, M.: 825-60, LB1545-440
Duan, Y.: 910-276, 1405-324, 4050
Duarte, M.: 1402-307, 3325-158
Dubbin, L.: LB2570-336, LB3620-419
Dubin, S.: 1345-110
Duchowny, K. A: 820-22
Dufour, S.: 1395-245
Duggan, A.: 2385-178
Duggleby, W.: 2430-293
Duke, B.: LB3620-376
Dulaney, S.: LB2570-336
Dull, G. E: 3355-249
Dummit, L.: LB935-443
Duncan, R.: LB3620-399
Dunkle, R. E: 890-222, 1075, 1345-111
Dunn, D.: 1350-120
Dunterman, R. A: LB3620-402
Dunterman, R. A: 815-14
Dupre, M.: LB2570-315
Dupuis-Blanchard, S.: LB1545-441
Durand, D.: LB935-417
Duru, O.: LB3620-408
Dury, S.: 575, 3035
Duthie, E. H: LB1545-400
Dutta, P.: 820-29
Dys, S.: 1305-27, 3345-214, LB3620-390
Dziedzic, P.: 2420-277
Dzierzewski, J.: 1390-239, 2420-272, 2425-282, 3370-288

E

Eagen, T. J: 855-148
Eaker, M.: 1410-330
Eapen, B.: 3295-76
Earhart, G.: LB3620-399
Earl, J.: 865-166
Eaton, J.: 1370-180, 3185
Ebert, A. R: 2355-78, 4140
Eby, D. W: 1410-326, 3325-171
Eckstrom, E.: 1360-160
Eddy, C.: LB3620-422
Edelman, L. S: 3355-249, LB935-400, LB2570-416
Edelstein, B.: 885-203, 3350-243, LB935-405
Edwards, A. B: 3315-122
Edwards, D. F: 2385-187, 2400-226
Edwards, E.: 610
Edwards, J.: 2555
Edwards, V. J: 1030
Edzards, S.: 815-15, 2340-44
Egan, M.: 3330-177
Egan, P.: LB3620-431
Egashira, R.: LB2570-320
Egbert, A. R: 1510, 2215
Ehrlich, J. R: 2495
Ehrlich, K.: LB2570-368
Ehsan, A.: 925-321
Eisa, N.: LB3620-330
Eisel, S. L: 1390-230
Eismann, M.: 660
Ejaz, F.: 1385-207, LB2570-383, LB3620-354
Ekerdt, D. J: 1375-188, 1490
Ekundayo, O.: 890-220, 3300-100
El Ibrahimi, S.: 3335-193
Eldadah, B.: 3260
Eldemire-Shearer, D.: 2065, 3045
Elfenbein, P.: 3005
Elhajjmoussa, L.: 4030
Eliazar-Macke, N.: 3320-137
Elkins, J.: 1360-155, 2045
Eller, M.: 1401-279
Ellerby, L.: 3215
Elliott, A. F: 3365-273
Elliott, E. M: 1345-116
Elliott, J. B: 3330-180
Elliott, K.: 900-248
Elliott, M. N: 520, 2250, 3430
Elliott, M.: 925-337
Elliott, S.: LB3620-390
Ellis, A.: 2390-201, 2395-211, 3295-74, 3300-98, 3345-228
Ellis, K.: 2325-26
Ellis, T.: LB3620-399
Ellison, C.: 2420-274
Ellor, J. W: 1475
Ellwardt, L.: 3245
Elman, A.: 2225, 2370-118
Elmansi, A.: 835-100
Elmansi, A. M: LB3620-330
Elmansi, A.: LB3620-360
Elswick, R. K: LB2570-356
Elton, J.: 1350-124
Ely, E.: LB2570-380
Emerman, J.: 1540
Emerson, K. G: 1300-12, 3320-135, 3360-271, LB935-424, LB3620-329
Emery-Thompson, M.: LB1545-431
Emery-Tiburcio, E.: 635
Emlet, C.: 1415, 2285
Enayati, M.: 820-37
Endo, Y.: 820-40
Engel, I. J: 2425-283
Engels, M.: LB2570-363
Engineer, C.: 2370-121, 2390-198
Englund, J.: 3305-106
Enguidanos, S.: 910-270, 1315-59, 1340-101, 3330-183, LB1545-443, LB2570-366, LB3620-433
Enroth, L.: LB1545-339
Ensrud, K. E: 820-22
Enzler, C. J: 2365-114
Eost-Telling, C. L: 3490
Eppler Hattab, R.: 1055
Epps, F.: 1510, 3100
Epps, F.: LB1545-350, LB3620-321
Erb, T.: 2270
Erbes, C.: 1390-236
Erickson, K. I: 715
Erickson, L.: 3290-45
Erik, B.: 1402-311
Eriksdotter, M.: 2215
Ermoshkina, P.: 3370-289
Ernst, J. S: 1000
Ernst, M. E: 870-180
Ernsth Bravell, M.: 2380-160
Ernsth-Bravell, M.: 3275-28
Erraguntla, M.: 1095
Errett, N.: 1405-320
Erving, C. L: 1390-233
Erwin, M. J: 815-12
Escarce, J.: 4050
Eskenazi, L.: LB3620-362
Espana, M.: 2405-239
Espeland, M. M: 850-132, 3590, LB2570-441
Espinosa Sempere, C.: LB935-430
Espinoza, S. E: 3325-160, LB2570-399
Esposito, M. H: 600
Estabrook, R.: 3340-206
Estabrooks, C.: 2430-293
Estapé, J.: 3070

Estapé, T.: 3070
Estepp, P.: LB3620-441
Estes, C.: 3015
Estrazulas, J.: 1402-312
Eto, M.: 820-27
Ettema, R.: 2380-158
Eugene, D.: 2385-192
Evans, D. S: 830-80
Evans, D. S: 1120
Evans, M. W: 3365-274
Evans, T. L: LB2570-390
Evans, W. J: 820-22
Everaars, B.: 675
Ewen, H. H: 680
Eymundsdottir, H.: 2370-133
Ezeah, P. C: 880-190

### F

Fabbre, V.: 3430
Faber, M. A: 1285
Fabius, C. D: 1110, 1375-187, 2270, 2405-234, 3585
Faghani Hamadani, T.: 2400-222
Fairchild, K.: 2535
Faizah, F.: 2400-230
Falck, R. S: 1510
Fales, K.: 2313
Fallahtafti, F.: 3295-80
Fall-Dickson, J. M: LB3620-443
Falzarano, F.: 655, 2520, 3290-49
Fane, M.: 3540
Fanelli, S.: 1315-55
Fang, B.: 1355-143, 2375-152
Fang, J.: 2385-175
Fang, M.: LB2570-336
Fang, Y.: 910-271, 3330-185, LB935-388
Fang, Y.: 2425-286
Fani, S.: 895-231
Farah, K.: 1420
Farina, A.: 1385-214
Farinatti, P.: LB2570-341
Farkas, C.: LB935-352
Farmer, H. R: 4120, LB2570-315
Farrell, M. T: 4110
Farrell, S.: 3325-167
Farrell-Savage, K. R: 2380-161
Farsijani, S.: LB935-368, LB2570-437, LB3620-348
Fashaw, S. A: 3585
Fast, J.: 2430-293
Fastenau, C.: LB935-349
Faul, A.: 1345-107, 1365-167, 2075, 2355-76, 3300-90
Faul, J.: 710, 1395-240, 2260, 2345-57
Fauth, E. B: 650, 3185, 3340-201, LB1545-394
Fawcett, J.: 3325-176
Fazeli, P.: 1360-156
Fazio, S.: 1160
Feaster, D. J: 1395-246
Fedarko, N.: 515
Federman, A. D: 1395-244, 3350-244
Fedus, D.: 2435-307
Feehan, L.: 1510
Feger, D. L: 2410-259
Feinberg, L. F: 1110, 3450
Feitosa, M.: 1310-42, LB1545-428
Feletti, L. C: 1402-298, 3325-159
Feliciano, L.: LB935-399
Felix, C.: 2265
Felix, L.: 2380-161
Felpeto, A.: 1315-45
Fendrich, M.: 2410-258
Feng, B.: LB1545-387
Feng, X.: LB1545-387
Fennell, G.: LB1545-341
Fennimore, T.: 3510
Fenstermacher, E. A: 815-9
Ferdous, F.: 2355-60
Ferdows, N.: 3395
Ferguson, C.: 2390-201
Ferguson, C.: 2395-211
Ferguson, C.: 3300-98, 3345-228
Fernandes, K.: 2360-87
Fernandez, J.: 1305-15
Fernández, S.: 3170
Fernández Ardèvol, M.: 580
Ferraro, K. F: 720, 730, 1420
Ferras, N.: 1402-301
Ferreira, E.: 1390-222
Ferrer, L.: LB1545-426
Ferrer, R.: 2340-45
Ferreri, S.: LB935-425
Ferri, C. V: LB935-433
Ferris, T.: 2185
Ferrucci, L.: 820-24, 835-99, 1180, 2290, 3040, 3075, 3405
Fetherstonhaugh, D. M: 505, 2395-210
Fick, D.: LB1545-397
Field, T.: 3355-256
Fielding, R.: 2190
Fields, N. L: 910-263, 1305-28, 2365-111, LB3620-405
Fierro, L. A: 815-15, 2425-288
Figiel, S.: LB935-417
Figueroa, A. C: 1380-202
Filho, B.: 2360-85
Fillenbaum, G.: 4145
Fillingim, R. B: 3330-184
Fillo, K. T: 2395-212
Finch, C. E: 825-50, 830-64
Finchum, T.: 1385-212, 2415-266
Fine-Smilovich, E.: 875-187
Fingerman, K.: 925-316, 1350-134, 2160, 3425
Fink, H.: 3455
Finkel, D. G: 2380-160, 3275-28
Finkelstein, E.: LB3620-396
Finkelstein, R.: 800, 3525
Finlay, J. M: 600, 1401-278, 2170, 2540
Finlayson, E.: 2115, 2315-1
Fiocco, A.: 820-20
Fiorentino, L.: 2420-273
Fiori, K. L: 4015
Firdauysa, N.: 1385-212, 2415-266, 3370-292
Fiscella, A. J: 3290-59
Fisher, A.: 830-71
Fisher, K.: LB1545-336
Fisher, N. M: 3175
Fisher, S.: LB2570-328
Fitzgerald, T.: 880-197
Flaherty, E.: 2075, 3545
Flanagan, J.: 1355-148
Flatt, J. D: 895-238, LB3620-419
Fleenor, J.: LB2570-322
Floegel, T.: 2410-249
Flood, K.: 2315-4
Flood, S.: 4065
Flores, B. E: LB1545-384
Flores, R. J: 1225, 3155
Flores Villavicencio, M.: 3290-67
Fogg, L.: LB2570-419
Fogle, S. J: LB935-393
Foley, K.: 915-289
Folz, R.: 545
Fong, T. G: 825-48
Fontes, F.: 2360-85
Forbes, D.: 3330-177
Ford, A.: LB935-401
Ford, K.: 1390-228
Fornalski, N.: 915-298
Forrester, S. N: 1400-260
Fortinsky, R. H: 2045, 2210, 2490
Fortner, J.: 900-245
Fossum, S.: 1410-329
Foster, M. J: 1360-151
Foti, M.: 3375-308
Fournier, C.: LB1545-419
Foust, J.: 3390
Fowler, M. E: 2315-14, 3595
Fox, A.: 1355-139
França, M.: 1390-222
Francke, A.: 4135
Franco, Y.: 850-129
Francois, E.: 2405-241
Frank, A. K: 1300-11
Frank, J. C: 3305-104
Franke, K.: 1365-168
Frankenstein, L.: 885-208
Franz, C. E: LB2570-348
Franzosa, E. C: 1155
Fraser, K.: 1395-245
Fratiglioni, L.: 515, 3170, 3325-173, 3420
Frazier, C.: 1390-233
Frazier, J.: 610
Freddolino, P.: 3415, 4020
Fredriksen Goldsen, K.: 1085, 1415, 2285
Freed, S. A: 1410-328
Freed, S.: 3290-61
Freedman, V. A: 2495
Freeman, I.: LB2570-376
Freeman, J.: 1350-124
Freeman, W. M: 820-25
Freiberger, C.: 890-217
Freijomil, F.: 2360-102
French, M.: 1505
Frenk, S.: 2355-70
Freshour, J.: LB2570-420

Freund, A. M: 3350-237
Frey, J.: LB3620-355
Freydefont, L.: 3350-237
Freytag, J.: 850-145, 3560
Freytes, I.M.: 840-108, 3320-137
Friberg Felsted, K.: 3495
Friedland, L.: 1135
Friedman, D. B: 3115
Friedman, E. M: 3275-19, 3350-238
Friedman, E. M: 4050
Friedmann, E.: 705
Frisoli, A.: LB1545-437
Fritz, H. A: 1360-164, LB1545-436
Fritz, M.: 3415, 4020
Froehle, A. W: 3170
Frolinger, T.: 825-57
Frook, G.: 1055
Frost, B. E: 3080, LB935-346
Fruhauf, C.: 1355-139, 1401-291, 1460, 3185, 3320-150
Frutos, B.: 2380-156
Fu, R.: 1400-265
Fu, Y.: 3365-277
Fuchs, J.: LB1545-334
Fugett, S.: 3275-26
Fuhrman, E.: LB2570-376
Fujita, K.: 830-63
Fujiwara, Y.: 855-154, 895-226, LB2570-411
Fullen, M. C: 3330-190, 4140
Fuller, H.: 1140, 3205, LB3620-441
Fuller, P.: 2405-241
Fuller-Thomson, E.: 2440
Fullwood, D.: 2410-264, LB2570-386
Fulmer, T.: 795, 3545
Fulzele, S.: LB3620-330
Fung, C.: 2420-273
Fung, H. H: 2425-286, 3460, LB935-361, LB1545-367, LB3620-427
Fung, N.: 3605
Funk, L. M: 2225
Funk-Lawler, R.: LB3620-358
Furber, J. D: LB3620-418
Furlano, J.: 3295-68
Fuselier, J.: LB1545-417
Futterman, A.: 2340-47, 2415-268, 3275-15

### G

Gaber, S. N: 3565
Gadzo, R.: 3330-187
Gaeta, L.: LB935-397
Gaffy, E.: 915-290
Gafni, A.: LB1545-336
Gagen, T. M: 1305-20
Gagne, H.: LB935-427
Gagnon, S.: 1410-329
Gahbauer, E.: 3315-127
Gaillot, S.: 2250
Gaines, B. E: 1260
Gaini, M.: 1360-154
Galambos, C. M: 1480, 4135
Galdamez, G.: 2320-19
Gale, S.: 3290-45
Galea, S.: 1345-116
Galenbeck, E.: 1402-314
Galik, E.: 920-301, 1255, 2390-194, 2525, 3270-11
Gallager, I.: 825-43
Gallagher, E. A: 915-288, 1380-201, 3290-66
Gallanis, T.: LB1545-442
Gallardo, E.: 820-18
Gallegos, C.: 1300-8
Gallo, H. B: 1405-319, 2540
Galvan, V.: 825-44, 835-98, 1310-38, 1520
Galvin, A.: LB2570-338
Galvin, J.: 3295-85
Gamaldo, A.: 2420-277
Gamaldo, A. A: 3290-53
Gamaldo, C.: 2420-277
Gambert, S.: 1402-309
Gammonley, D. L: 910-269
Ganai, S.: 1360-161
Ganann, R.: LB1545-336
Ganapathy, V.: 835-91, LB3620-372
Gangai, N.: 635, 1365-174, 3275-20, 3305-105, 4110
Ganz, N.: 1402-303
Gao, H.: LB2570-368
Gao, L.: 4030
Gao, M.: 3370-296
Gao, Q.: 1515
Garbarino, J. T: 900-244
Garbarino, V. R: 825-56
Garber, L.: 3355-256
Garcia, A.: 2380-163
Garcia, C.: 3070, 4000
Garcia, D.: 835-83
Garcia, M. A: 3070
Garcia, M.: 3355-255
Garcia, M.: 2280, 4035
Garcia, Y.: 1350-120
García, I.: 1305-16
García-Peña, C.: 3590, 4155
Garcia-ptacek, s.: 2215
Gardenhire, D. S: 920-312
Gardiner, P.: 570
Gardner, D. S: 3445
Garland Baird, L.: 1395-245
Garm, A.: 3325-172, 3330-189
Garner, K. K: 920-311, 2365-106
Garonzik Wang, J.: 2380-168
Garrett, S.: 545
Garrison, B.: 1340-99, 1475
Gartz, R.: 1410-330
Gassoumis, Z.: 910-270, 2320-19, 2370-118, 2375-150, 2385-193, 2465, 3025
Gately, M.: 1295
Gaudet Hefele, J. L: 740
Gaugler, J. E: 885-207, 1005, 1385-214, 1401-278, 1495, 2325-21, 2355-71, 2375-140, 2490, 4025
Gauthier, C.: 3280-29
Gauthier-Beaupré, A.: 2360-98
Gaveras, E.: 3430
Gavrilov, L.: LB3620-395
Gavrilova, N. S: LB3620-395
Gazit, E.: 1402-303
Gazzola, J.: 2360-85
Ge, M.: 2095
Ge, S.: 4115
Gea-Sánchez, M.: 3345-216
Gebelin, K. D: 1150
Gebremariam, M.: 905-251
Gee, N. R: 705
Geirsdottir, O.: 2370-131
Geirsdottir, O.: 2370-133
Gelb, B.: 885-212
Geldmacher, D.: LB2570-391
Gell, N. M: 920-304, 2360-89
Gellis, Z. D: LB2570-442
Gelman, C.: 3065, 3445
Gendron, T.: 2285, 3340-205, 4170
Gensburg, L.: 3325-163
Gentes, R.: 3180
Georgousopoulou, E. N: 705
Gepshtein, S.: 500
Gerald, L.: LB2570-445
Gerard, F.: 2385-178
Gerard, S. N: 3270-7
Gerhard, L.: 1235
Gerhart, O.: LB1545-357
Germack, H.: LB3620-386
Germain, C. M: 2380-167, 3155
Gershon, R.: 3595
Gerstorf, D.: 2275, 4015
Gertel, V.: 3295-69
Geshell, L.: 3105
Getz, M. A: 2435-305
Geva, A.: 1402-293
Ghag, C.: 715
Ghaul, M.: 3375-307
Ghazi, A.: 3540
Ghazi Saidi, L.: 3295-88
Ghiasi, A.: 910-262, 2395-218, 3345-213, 3435
Ghosh-Dastidar, B.: 4050
Gibb, Z.: 2385-191
Gibbs, L.: 3320-140
Gibson, A.: LB2570-415
Gibson, A.: 1305-14, 1340-99, 2060, 2450
Gibson, P.: LB3620-329
Gideon, C.: 1320-63
Gidmark, S.: 3435
Gifford, D.: 3435
Gil, A.: 2360-87
Gil, E.: LB1545-375
Gilbert, K. L: 3295-78
Gilbert, S. B: 1270
Giles, G. M: 2385-187, 2400-226

Gill, L. L: LB3620-340
Gill, T. M: 1400-262, 3315-127, LB3620-403
Gillespie, P.: 920-299
Gillespie, R.: 2425-289
Gillespie, S.: 910-275
Gilliam, M.: LB3620-426
Gilligan, M.: 2035, 2160, 2365-110, 3315-131
Gills, J.: 1402-300
Gilmartin, M. J: LB1545-335
Gilmore-Bykovskyi, A.: 3350-240
Gilpin, A.: 1390-232
Ginis, P.: 1402-304
Ginther, J.: 3275-26
Ginting, M. L: 2360-95
Giordani, B.: 500
Giordano, L.: 520
Gioscia-Ryan, R. A: 835-89
Girgus, J.: LB935-433
Girling, L.: LB3620-413
Girouard, H.: 3280-29
Gitlin, L. N: 770, 885-207, 1005, 1495, 2355-79, 3110, LB2570-353
Giuliano, R.: 825-53
Giunta, N.: 3445
Gjestsen, M.: LB3620-317
Gladyshev, V.: LB3620-407
Glaser, K.: 1350-130
Glass, A. P: 2150, 2430-298
Glazner, G.: 1075
Gleason, K.: 1401-291
Glenn, J. M: 1402-300
Glicksman, A.: 1070, 1210, 1500, 2150
Glorioso, T.: 3390
Glover, C. M: 2155, LB3620-375
Glynn, N. W: 1240, LB935-368, LB1545-428, LB2570-343, LB3620-403
Glynn, R.: 3325-169
Gmelin, T.: 1240, LB1545-428, LB2570-343
Gnanasekaran, G.: 2315-13
Gnjidic, D.: 3365-278
Godfrey, M.: 1360-165
Godin, J.: 565, 3325-165
Godina, S. L: 2170
Godoy, M.: 2310
Goel, R.: 2360-99
Goeman, D.: 2325-26
Gogol, M.: 930-344, 1490
Goktas, S. O: 815-17
Goldberg, R. W: LB1545-371
Golden, A.: LB3620-341
Golden, R.: 2385-174
Goldgof, D.: 1310-39
Goldman, T.: 695
Goldsmith, S.: 1365-179
Goler, T.: 895-239
Goltz, H. H: 1380-190
Golub, J. S: 2495
Gomez, F.: LB2570-399
Gomez-Orozco, C.: 1330-94
Gomez-Orozco, C.: 1402-299
Gómez-Verján, J.: 3590
Gonyea, J. G: 1010
Gonzales, E.: 4150
Gonzales, M.: LB3620-372
Gonzalez, A. L: 1320-64
Gonzalez, R.: 3190, LB2570-435, LB3620-331
González, H. M: 860-159, LB3620-342
Gonzalez-Marquez, A.: 890-224
Goodridge, D.: LB2570-424
Goodwin, J. S: 3355-250
Goodyear, M.: LB3620-379
Gooneratne, N.: 920-305
Gorbunova, V.: 525
Gordon, S.: 3320-145
Gore, S.: 1320-65
Gorenko, J. A: 2340-40
Gorges, R.: 2480
Gorospe, M.: 835-99
Gorospe, M.: LB935-347
Gorzelle, g.: 500
Gorzelle, G.: 2355-63
Gosdin, M. M: 3010
Goswami, S.: 3350-235
Gotelli, J.: LB935-392
Goto, J.: 925-319
Gott, M.: 2270, 3105, 3310-112
Gottesman, R.: 2495, 3075, LB2570-401
Gotwals, A.: 1230
Gould, C. E: 1390-238, 1401-286, 2535
Gould, O.: LB1545-441
Goulet, J. L: 2370-119
Govier, D. J: 3370-300
Gozalo, P. L: 520, 740, 2325-21
Graber, T. G: LB1545-354, LB2570-325
Graboski-Bauer, A.: 890-224
Grabowski, D. C: 1375-185
Grace, R.: LB3620-441
Gracner, T.: 2395-217
Graf, A. S: 1380-195
Graf, P.: LB2570-438
Grafova, I. B: 4135
Graham, C.: 1210, 2150
Graham, E. K: 585, 3340-206, 3575
Graham, G.: 915-293
Graham, K.: LB3620-375
Graham, K.: 3125
Graham, K.: 3300-97
Graham, L.: 3305-102
Gramlich, C.: 850-140
Granbom, M.: LB2570-353
Grande, G.: 850-137, 3170
Grandinetti, A.: LB1545-392
Granger, B.: LB2570-315
Grant, A. N: LB2570-327
Grasselli, C.: 820-31
Gratton, C.: 840-114
Graupner, J. P: 3305-108
Gravenstein, S.: 4130
Graves, J. L: 1240
Gray, D. A: 820-18
Gray, J. A: 1365-172
Gray, M.: 1402-300
Gray, S.: 855-147
Gray-Miceli, D. L: 2360-103
Greaney, M. L: 1170, 1400-251, 3600
Greaves, C. T: LB935-398
Green, A.: 545
Green, C.: 2365-114
Green, D.: LB2570-414
Greenberg, J. S: 2375-148
Greenberg, P. A: 815-4, 900-248
Greenberg, S.: LB1545-335
Greene, M.: LB3620-419
Greene, N. A: 1300-13
Greene, R. R: 1300-13
Greenfield, E.: 595, 720, 2150, 2440, 3065, LB1545-383
Greenfield, J. C: 845-116
Greenwood-Hickman, M.: 570
Greer, J.: 910-263
Gregg, S.: 3330-180
Gregory, M. A: 1310-40
Greig, C. A: LB935-390
Greig-Custo, M. T: 2355-66
Grenier, A. M: 905-254, 3085
Grice, J.: 2415-266
Griffin, J. M: 880-193, 3180
Griffin, S.: 2425-282
Griffin, T.: LB2570-394
Griffiths, P. C: 840-107, 850-125, 1360-155
Griffiths, P.: LB1545-350
Grimaldi, A.: 2430-297
Grimm, K.: 855-150
Grimm, K.: LB2570-402
Grinshteyn, E.: 2365-113
Griswold, M.: LB2570-401
Gross, A.: 830-75
Gross, A. L: 2265
Gross, A.: 2370-129
Gross, A. L: 2410-259, 3075
Gross, E. Z: 2365-112
Gross, M.: 1402-293
Growney, C.: 1385-216, 4095
Gruber, J.: 3195
Gruber, M.: 2325-25
Gruber-Baldini, A.: 820-21, 1402-311, 2210, 2370-122
Grudzien, A.: 1390-237
Gruman, C. A: LB935-443
Grunwald, O.: 575, 865-170
Gruss, V.: 2270
Gu, D.: 1515
Guaita, A.: 2425-281
Guan, A.: LB1545-385

Gubrium, J. F: 1185
Gudkov, A.: 4090
Gudnason, V.: 2370-133
Guernsey, M.: LB3620-402
Guerra, L.: 2075
Guest, M. A: 2500
Gugliucci, M. R: 1370-181, 3005, LB935-395, LB3620-424
Guidoboni, G.: 820-37
Gullapalli, R.: LB1545-424
Gummi, S.: 3140, LB935-416
Gunn, L.: 860-156
Gunzler, D. D: 835-86
Guo, G.: 1297
Guo, M.: 615, 2010
Guo, M.: 1400-261
Gupta, R.: 2250
Guralnik, J.: 570
Guralnik, J.: 820-21, LB3620-355
Gurari, D.: LB2570-330
Gurevitch, J.: 1390-221, 3300-97
Gurinovich, A.: 835-92, 1120
Gurvich, T.: 2120
Gurwitz, J.: 3355-256
Gur-Yash, N.: 2315-7, LB1545-375
Gusmano, M.: 1065
Gutierrez, C.: 1365-171
Gutiérrez-Robledo, L.: 3590, 4155
Guzman, J.: 3360-266

### H

Ha, J.: 2375-148
Haaga, J.: 2290
Haas, A.: 850-139, 4135
Haas, K.: LB1545-387
Haavisto, W.: 3290-47
Habtemariam, D.: LB1545-389
Hackett, P.: 2355-62
Hackney, A.: 705, 1090
Hackney, M.: 920-307
Hackney, M.: 3020
Hackney, M. E: LB3620-336
Hadad, A.: 1402-303
Haderlein, T.: 1170
Hadley, E.: 1120, 2290
Hagedorn, A.: 3400
Hagemann, L.: 1355-145
Hagg, S.: 625
Haghani, A.: 830-64
Hahn, J. S: 2100
Hahn, S. J: 735
Hahnel, T.: 1385-218
Hait, Y.: LB1545-375
Hajat, A.: 855-152
Halasz, I.: LB1545-363
Hale, A.: 1402-314
Hale, L.: 2420-274
Hale Gallardo, J.: 2405-238
Haley, W. E: 1170, 1250, 1355-136, 2355-69, 2270, 2375-141, 2435-311, 3360-267, LB3620-433
Hall, C. B: 845-124
Hall, J.: 2370-118
Hall, K. S: 530, 630, 1330-83
Hall, L.: 2365-112
Hall, L. O: 1310-39
Hall, L. C: 4170
Halli-Tierney, A. D: 870-183
Halli-Tierney, A.: 900-243
Halli-Tierney, A.: 1315-56, LB1545-332, LB2570-418
Halonen, P.: LB1545-339
Halpern, D.: 1305-24
halpern, J.: 2010
Halpin, S. N: 2385-176
Halvorsen, C. J: 1540
Ham, C. R: 2385-186
Hambarsoomian, K.: 2250
Hamdani, M.: 1320-64
Hamel, K.: LB2570-412
Hamers, J.: 2020
Hamilton, A.: LB3620-391
Hammond, N. G: 1380-198
Hampstead, B. M: 500
Hamrick, M.: 830-73, 835-100, LB3620-330
Han, A.: 1365-178, LB2570-378
Han, C.: 1400-258
Han, D.: 2400-223
Han, G.: LB3620-315
Han, H.: 3360-268
Han, H.: 3325-174
Han, J. H: LB2570-380
Han, J.: 1245
Han, L.: 2370-119, 3365-278
Han, S.: 1250
Han, S.: LB935-445
Han, S.: LB1545-358
Han, X.: LB935-348
Han, Y.: 500
Han, Y.: 1355-144
Hancock, C.: 1035, 2025
Hand, M.: LB3620-401
Handing, E.: 805
Handy, D.: 1345-107
Handy, T. C: 715
Hangar, J.: LB1545-439
Hank, K.: 3245
Hanks, R. S: 2560
Hanlon, J.: 3390
Hanna, G. P: 610
Hannon, P.: 2410-256
Hannum, S. M: 3275-14
Hanratty, B.: 1290, 1325-67, 2395-213, 4055, LB1545-418
Hansen, A.: 3295-74
Hansen, L.: 3010
Hanson, K.: 3305-109
Hanson, L.: 520
Hanson, L.: 2300
Hansson, I.: 575
Hantman, S.: 1300-13, 1375-183
Hao, N.: 1115
Hao, Z.: LB1545-376
Haran, J. P: 820-29
Harari, L.: 3340-203, 4145
Hardgraves, V. M: LB1545-343
Hardin, H. K: 1330-75
Harding, M.: 2365-106
Hardland, J.: 1335-97
Hardy, R.: 3170
Harfmann, E.: 1355-142
Harfmann, E.: 2375-149
Hargis-Fuller, L.: LB935-384
Hargittai, E.: LB1545-334, LB2570-377
Harling, G.: 4110
Harllee, H.: 3375-311
Harllee, H. D: 3375-305
Harlow-Rosentraub, K.: 1345-111
Harman, S. M: 3325-176
Harmon, A.: 1305-21
Harootyan, L. K: 2085
Harootyan, R.: 1085, 2085, 3285-38
Harralson, T.: 915-281
Harrati, A.: 1430
Harrington, A.: 510, 3555
Harrington, A.: 505
Harrington, C. C: 2505
Harrington, E.: 1401-285
Harrington Meyer, M.: 3245
Harris, A. P: 1405-321
Harris, J. A: 3345-222, 4050
Harris, M. T: 3500, LB2570-406
Harris, M. L: 850-127
Harris, R.: LB1545-363, LB2570-409
Harris, R.: LB2570-445
Harris, S. M: 2340-47
Harris, T. B: 1425
Harris-Kojetin, L.: 3060
Harrison, K.: 1305-33, 2010
Harrison, T. C: 920-314
Hartikka, K.: 3410
Hartmann, C. W: 740, 3535
Hartz, S.: 1335-97
Harvath, T.: 2295
Hasan, N.: 2400-230
Hasebe, M.: 895-226
Hasegawa, M.: 830-63
Hasnain, M.: 2270
Hass, Z.: 3435, LB935-377
Hassett, A.: 1425
Hassouneh, D.: 840-104
Hastings, S.: LB3620-325
Hastings, W. J: 2370-117
Hasworth, S.: 910-277
Hasworth, S.: 3345-214
Hasworth, S.: LB2570-404
Hata, C.: 2390-199
Hathaway, D.: 2410-254
Hatton, S. N: LB2570-348
Hatzifilalithis, S.: 905-254
Hauer, K.: 920-306
Haugen, C.: 2380-168, 4020
Hausdorff, J. M: 1402-304, 4020
Haverhals, L. M: 1345-105, 1402-314, 4135

Haviland, A.: 2250
Havyer, R.: 3180
Hawkins, B.: 505
Hawkins, N.: 2385-175
Hawkley, L.: 2275
Hay, M.: LB3620-441
Hayanga, B. A: LB935-418
Hayden, K.: 805
Hayslip, B.: 925-333
Hayslip, B.: 1460
Hayslip, B.: 2375-147
Hayward, M. D: 1200
Hazuda, H.: LB3620-372
He, M.: 3390
He, W.: 1325-74
Healey, W.: 4030
Hearld, L.: 3345-211
Heckman Coats, A. L: 2435-312
Hedeker, D.: 2400-221
Hees, E.: 550
Heffner, K. L: 670
Heflin, M. T: 1305-24
Heijkants, C.: LB1545-414
Hein, M.: LB2570-364
Heinz, M.: 645, 2390-205, 3300-92
Heisel, M. J: 3105
Heisler, C. J: 1265
Heitkamp, R.: 1335-95
Heldmann, P.: 920-306
Helgeson, V.: 1315-48
Helm, F.: 3465
Helphrey, J. H: 815-15, 2425-288
Helvik, A.: 1330-86
Hemmady, S. A: 2410-256
Hemmert, R.: 3355-249
Henage, C. B: 4075, LB935-392
Henderson, C.: 3220
Henderson, S.: 3010
Henderson, T. L: 3315-122
Hendricksen, M.: LB1545-389
Hendrix, C. C: LB1545-425
Hendry, L.: 1315-49
Henkens, K.: 660, 865-170
Henry, J.: 3330-181, LB1545-343
Henry-Smith, C. A: LB935-348
Heo, J.: 2410-246, 3370-297
Hepburn, K.: 710, 840-107, 850-125, 1510, LB1545-350
Heppner, A.: 690
Herbert, M.: 4080
Herd, A.: 2395-210
Herd, P.: 720
Herkert, N.: LB2570-322
Herman, H.: 2510
Hermesch, A.: 690
Hermida, R.: 910-274
Hernandez, A.: 2310
Hernandez, D.: 2395-215
Hernandez, D. F: 1330-93
Hernandez, M. E: 4020
Hernandez, S. C: 1330-84
Hernandez, S. F: 1310-38
Hernandez-Lujan, D.: 1345-105
Hernandez-Tejada, M.: 1055, 2365-107
Herr, K.: 3365-282
Herr, N.: 815-9
Herrera Legon, M.: LB1545-353
Herrera-Venson, A.: 3415, 4030
Herron, R.: 2105, 2225
Hershey, D.: 865-166
Hertzog, C.: 4095, LB2570-373
Hervera, B.: LB1545-426
Hess, T. M: 1385-216, 2275, 2340-34, 4095
Hetzel, S.: LB935-422
Heyman, J.: LB2570-340
Heyn, P. C: 770, 1445
Hiatt, S. O: 3010
Hibbs, C.: LB2570-346
Hicken, B.: 2055, 3165, 3485
Hicks, G.: 3365-275
Hicks, H. J: 500, 2410-250
Hicks, N.: LB2570-349
Hicks Patrick, J.: 890-221
Hicks Patrick, J.: 925-334, 1065, 1475
Higgins, M.: 850-125
Higgins, M.: 850-128
Higgins-Chen, A. T: LB1545-422
Hilgeman, M. M: 2535, LB3620-339
Hill, C.: 545
Hill, C.: 1050, 1450, 2240, 3095
Hill, C.: 3385
Hill, J. E: 865-168
Hill, J.: 865-168
Hill, M. S: 865-168, 1401-277, 3290-45
Hill, N.: 1175
Hill, N.: 3295-89
Hill, P. L: LB935-386
Hill, R.: 1355-148
Hill, T. D: 2420-274
Hill, W. D: 830-73
Hill, W. D: 835-100
Hill, W. D: LB3620-330
Hillel, I.: 1402-304
Hilsabeck, R.: 850-134
Hincapie, D.: 1315-45, 1330-82
Hines, D.: 850-144
Hinton, L.: 3010
Hirano, H.: 855-154
Hirano, H.: 895-232
Hirano, H.: LB2570-411
Hirayama, R.: 880-202, 2375-151
Hirofuji, T.: LB1545-344
Hirsch, C.: 4045
Hirsch, J. A: LB1545-364
Hirschman, K.: 840-109, 915-289, 1080, 2215, 3230
Hirukawa, S.: 3270-4
Hladek, M. D: 725, LB2570-353
Ho, G.: 3245
Ho, J. Y: 3245
Ho, S.: 3290-65
Ho, S.: 2360-95
Ho, T.: 3460
Hobbs, M.: 3060
Höbler, F.: 850-142
Hochberg, M.: 820-21
Hodes, R.: 2135, 2290
Hodgdon, B.: 3180
Hodgkinson, J.: 3345-224
Hodgson, N.: 605, 915-289, 1160, 2215, 2420-276, 2490
Hoe, D.: 3330-183, LB2570-366
Hoeft, T. J: 3010
Hoek, L. J: 785
Hoens, S.: 3035
Hofer, S.: 3340-206, LB2570-355
Hoffman, G. J: 4130
Hoffman, J. M: 2230, LB2570-371
Hoffman, R.: 820-18
Hogan, C.: 3365-280
Hogan, D. B: 3295-87
Hohn, K. L: 3430
Hojfeldt, G.: 820-30
Holahan, C. K: 1395-247, 3335-196
Holahan, C. J: 3335-196
Holland, D.: 3180
Holland, E.: 1320-64
Holley, L.: 2500, 3345-225
Hollinda, K.: 2325-30
Hollis-Sawyer, L. A: 1380-197, 3275-18
Hollstein, B.: LB3620-392
Holm, L.: 820-30
Holmes, A.: 2360-101
Holmes, H. M: 1225
Holmes, S.: 1255
Holmes, S. D: 3270-11
Holt, H.: 1505
Höltge, J.: 1330-88
Homan, K. J: 720
Homayouni, R.: LB935-366
Homeier, D.: 3025
Hommelhoff, S.: 1385-218
Honda, T.: 1370-180
Hong, J.: 2375-148
Hong, M.: 1340-100, 2140, 2405-240
Hong, M.: LB2570-337
Hong, Y.: 1360-151
Honig, L.: LB2570-396, LB3620-337
Hoogland, A. I: 1390-230
Hooker, K.: 1195, 1350-125, 2405-237, 3235, 3370-300
Hooker, S.: 890-223, 1095
Hooyman, N.: 2313
Hopfgartner, I.: 2390-206
Hopp, F. P: 1000
Hoppmann, C. A: LB1545-445, LB2570-438, LB3620-383
Horgas, A.: 3365-273
Horhota, M.: 815-5
Horowitz, A.: 2520
Hossain, M.: 2400-230
Hou, S.: 1040, 1500, 2150
Houlind, M.: LB2570-339
Hounsell, C.: 695

Houser, A.: 3450
Houston, A.: 1355-142
Houston, A. M: 2375-149
Hovey, S.: 3495
Howard, A.: 3360-270
Howard, E. P: 4080
Howard, S.: LB3620-341
Howard, S.: 1402-310
Howard, V.: 1170
Howe, J. L: 2220, 3530
Howe, M.: 3360-270
Howell, B. M: 1325-66, 2225, LB935-360
Howlett, S. E: 820-36, LB2570-323
Hoy, C.: 855-147
Hoyle, J.: 1345-117
Hoyle, J.: 2375-139
Hoyt, E. K: LB1545-405
Hsiao, H.: 3400
Hsieh, J.: 905-255
Hsieh, J.: LB935-444
Hsieh, W.: 3360-270
Hsiung, G.: 715, 920-313
Hsu, C.: 565, 715, 2215, 2360-97
Hsu, E.: 2345-53
Hsu, F.: LB1545-390
Hsu, J.: 1310-37
Hsu, J.: 2360-82
HSU, S.: 1075
Hsu, W.: 2155
Hu, M.: LB935-409
Hu, R. X: 1350-121, 2495, 3250, 3275-25
Hu, S.: 890-219, 1330-89
Hu, Y.: 3335-195, 3365-283
Hu, Y.: LB1545-436
Hua, C.: 4150
Hua, J.: LB935-384
Hua, P.: 840-115
Hua, Y.: 4105
Hua, Y.: LB3620-430
Huang, A.: 2425-291
Huang, C.: 905-250
Huang, F.: 1390-225
Huang, F.: 1400-257
Huang, F.: 3395, 4160
Huang, H.: 1300-12, 3360-271
Huang, J.: 1170, 2280, 3420
Huang, N.: 890-219
Huang, V.: 820-20, 3295-87
Huang, W.: 3085
Huang, Y. A: 2380-163
Huang, Y.: 2370-128
Huang, Z.: 1330-77, 2370-137, 4005
Hubbard, E.: 1330-91
Hubbell, Z.: LB3620-341
Huber, G.: 4030
Huber, H.: 835-94
Huber, L. L: 900-241, 1370-182, 1401-271, 3375-306
Huber, N.: 825-43
Hubner, S. B: 3350-234
Hudson, J.: 1015
Huebner, J.: LB2570-394
Hueluer, G.: 590
Huff, M.: 1345-107, 3300-90
Huffman, K.: 4130
Huffman, M. K: 2410-255, 3350-238
Hugenschmidt, C.: LB935-403
Hugenschmidt, C. E: LB2570-401
Hugentobler, L.: LB1545-334
Hughes, A. K: 3415, 4020
Hughes, M. L: 3350-246
Hughes, M. L: 700
Hughes, S.: 1401-273
Hughes, S.: 3175, 3305-107
Hughes, T. F: 1360-153
Hughes, T.: LB2570-401
Huh, T.: 1390-238
Huisingh-Scheetz, M.: 4070, 4165
Hummer, R.: 2280
Humphries, J.: LB1545-439
Hundza, S.: 2360-105, 3470
Hung, L.: 1060, 1401-292
Hung, M.: 660
Hung, W.: 3165
Hunsaker, A. E: LB1545-334, LB2570-377
Huo, M.: 725, 925-316, 1250, 1350-134, 3580
Hurtado Pomares, M.: LB935-430
Hurwitz, E.: LB1545-392
Husa, R.: 2405-238
Hussong, S. A: 825-44, 835-98
Hutchins, D.: 850-140
Hutchins-Wiese, H. L: LB2570-427
Huynh, A.: 1170
Hwang, S.: 2355-69
Hwang, U.: LB3620-325
Hwang, Y.: 915-289
Hyer, K.: 1365-177, 1405-325, 1530, 2220, 2400-220, 3345-221
Hyun, J.: 3290-61, 4085, LB2570-347

### I

Iaboni, A.: 2355-61, 3415
Iacob, E.: 500
Iapalucci, M.: 2415-269
Ibrahim, S.: 915-293
Ibrahim, T.: LB2570-324
Idler, E.: 3340-207
Igarashi, A.: 895-225
Igarashi, H.: 1350-123
Ighodaro, E.: 2270
Ihara, E. S: 3305-103
Ihara, K.: LB2570-411
Iijima, K.: 895-232, 3325-157
Ikard, C.: LB1545-332
Ikeuchi, T.: 3340-204
Ilardo, J.: 1305-29, 4005
Ilkayeva, O. R: LB2570-437
Iloputaife, I.: LB3620-328
Im, K.: 2435-309
Im, S.: 3370-297
In, L.: 2120
Inagaki, A.: 895-225
Infurna, F. J: 1215, 1350-129
Infurna, F.: LB2570-402
Ingber, M. J: 2385-190
Ingels, J. B: LB2570-370
Ingersoll-Dayton, B.: 3430
Ingram, C.: 3180
Ingram, E.: 1330-80
Ingram, F.: 3590
Ingram, R. E: LB935-399
Inker, J.: 505, 815-11, 3340-205, 4170
Inoue, M.: 1070, 1260
Inouye, S.: 2245, 4130
Intrator, O.: 4045
Intrieri, R. C: 815-13, LB3620-402
Inventor, B. R: LB2570-419
Iqbal, A.: 890-218
Iqbal, U.: 825-57
Irani, E.: 725
Irarrázabal, L.: LB1545-426
Irimia, A.: 825-61, 3080
Iris, M.: LB935-377
Irizarry-Arroyo, N. E: 1390-230
Irvin, V. L: 2405-237, 3370-300
Isaacowitz, D. M: 1385-209, 2110
Isales, C.: 830-73, 835-100, LB3620-330
Isbel, S.: 705
Ishida, A.: 3280-30
Ishikawa, Y.: 1400-256
Ishioka, Y.: 1345-109
Ishioka, Y. L: 3340-199
Ishizaki, T.: LB2570-444
Ismail, N.: 2360-96
Ismail, Z.: 3295-87
Israel, S.: 3025
Israel, S. M: 915-292
Issachar, G.: 1402-293
Iveniuk, J.: 1345-108
Ives, D.: LB2570-396
Iyer, S.: 1390-238

### J

Jablonski, R. A: LB2570-391
Jabson, J.: 895-238
Jacelon, C.: 930-342, 1395-249, LB1545-403, LB2570-372
Jachim, S.: 830-67
Jackson, E.: 1400-267
Jackson, L.: 1400-267
Jackson Stoeckle, R.: 795
Jacob, M.: LB3620-372
Jacobs, E. A: 930-343, LB935-422
Jacobs, J. M: 2315-9
Jacobs, L.: 535, 1355-145, 3125, 1390-221
Jacquelin, B.: 2430-302
Jagannathan, S.: LB3620-424

Jagger, C.: 685, 1200, LB2570-319
Jahoor, F.: 1310-37, 2190
Jain, I.: 2090
Jain, S.: LB2570-387
James, B.: 1390-230
James, B. D: 3480, 3575
James, D.: 2315-4
James, N.: LB-935-447
James, N.: 1355-140, LB1545-353
Janet, L.: 3320-134
Janet, M.: LB1545-336
Janevic, M. R: 705, 775, 3365-280
Jang, A.: 1402-305
Jang, D.: 3600
Jang, H.: 640
Jang, I.: LB2570-375
Jang, J.: 2360-83
Jang, J.: 600
Jang, J. Y: LB3620-411
Jang, K.: 1350-126
Jang, Y.: 1325-70, 2140, 2405-235, LB3620-433
Janssen, L. M: LB935-407
Jao, Y.: 850-133, 3010
Jarosek, S.: 2155
Jarrett, T.: 2385-176
Jarrín, O. F: 850-143, 910-274, 4135
Jarrott, S. E: 1205
Jarrott, S.: 2405-239
Jarrott, S. E: 3235
Jarrott, S.: 3290-62, 3315-128
Jarvis, L.: 530, 1402-308
Jason, K.: 1055, 1160
Jasper, A.: LB3620-410
Jayanama, K.: 3325-165
Jazwinski, S.: LB1545-417
Jazwinski, S.: LB1545-377
Jeanblanc, A.: 890-216
Jeanblanc, A. B: 2385-177, 3320-141
Jean-Louis, G.: 2325-29
Jeffery, B.: 1515, 2355-62, 2430-296, 3140
Jehu, D. A: 2360-90
Jen, S.: 3350-242, 4145
Jenkins, M.: LB2570-331
Jenkins Morales, M.: 910-260
Jenney, K. N: 2360-86
Jennifer, L.: 3320-137
Jennings, B. M: 840-107, 850-125
Jennings, J.: 925-324
Jennings, S. C: 630
Jensen, A. C: 1401-277
Jensen, C. J: 505, 2300
Jensen, J.: 2365-106
Jensen, P. H: 3520
Jeon, S.: 1305-33
Jeon, Y.: 505
Jeong, M.: 3350-242
Jervis, L. L: 3310-111
Jespersen, B. V: 3360-261
Jester, D. J: 1355-136, 1365-177, 2400-220, LB2570-362
Jette, N.: 555
Jia, C.: 3350-241
Jia, R.: LB3620-428
Jia, W.: LB935-356
Jiang, D.: LB3620-397
Jiang, H.: LB2570-333
Jiang, J.: 3320-142
Jiang, M.: LB935-356
Jiang, N.: 1340-103, 3250, 4115
Jicha, G. A: 2270
Jim, H. S: 1390-230
Jimenez, D.: LB935-417
Jimenez, D.: 2520
Jimenez, E.: 2425-284
Jimenez, E. E: 1170
Jiménez, M.: 3370-290
Jin, H.: 4005
Jing, B.: 2315-1
Jiwani, H.: 2315-15
Jiya, N.: 3290-63
Joergensen, T.: LB1545-356
Johansson, B.: 2215
Johansson, L.: 2380-160
Johari, K.: 3330-182
Johnson, B. P: 2315-6
Johnson, D.: 3175
Johnson, I. M: 2540
Johnson, I.: 1315-46
Johnson, J. P: 4020
Johnson, J. E: 765
Johnson, J.: 2215
Johnson, J.: 1160
Johnson, K.: 3560
Johnson, K. S: 520
Johnson, K. J: 2430-299
Johnson, K. S: LB1545-425
Johnson, K.: 2395-207
Johnson, M.: LB935-402
Johnson, M. R: 2345-55
Johnson, M. L: 895-237
Johnson, N. L: 1380-199
Johnson, R. E: 880-189
Johnson, S.: 2355-62
Johnson, T.: 815-4
Johnson, T.: 1315-52
Johnson, V.: 1380-195
Johnson-Lawrence, V.: 1450
Jolejole, K.: 730
Joling, K.: 4135
Jones, C.: 1405-322
Jones, C. A: 920-315
Jones, G.: 1180
Jonnakuti, S. T: 1310-34
Jonsdottir, M.: 2370-133
Jonsson, P. V: 2370-131
Joo, H.: 2385-175
Joo, S.: 1360-159, 3340-208, LB2570-362
Jopp, D.: 1385-213, 2340-37, 3315-123, 3370-286
Jordan, L.: 1170, 4010
Jordan, M.: 840-108
Jor'dan, A. J: LB3620-328
Jorritsma, J.: 3230
Jose, R.: LB2570-414
Joseph, L.: 545, 3455
Josephson, K.: 2420-273
Joubert, S.: 3280-29
Ju, C.: 3310-116, LB935-382
Juang, C.: 1390-238, 1401-286
Juárez Leal, I.: LB935-430
Juckett, L.: 1205
Juckett, L. A: 3315-128
Judge, S. T: 4100
Jukema, J. S: 3230
Julien, C.: LB1545-419
Jun, H.: 1360-159, 3340-208
Jun, J.: 1300-4
June, A.: 2435-307, 3370-291
June, J.: 1405-325, 3345-215
Jung, H.: 3325-174, LB2570-375
Jung, H.: LB1545-398
Jung, J.: LB2570-337
Jung, M.: 2340-51
Jung, S.: 3370-286
Jung, S.: 645, 2390-201, 2395-211, 3300-98, 3345-228
Jurkeviciute, M.: 1402-298
Jurkowski, E. T: 1335-95
Justice, J. N: 835-89, 1450, 2530, 3440, 4090
Jutkowitz, E.: 2325-21, 2490
Jylha, M.: LB1545-339
Jyothi, K.: 1402-310

### K

Kaasalainen, S.: 910-264, 1395-245
Kaczorowski, C.: 3080
Kadylak, T.: LB1545-373
Kaeberlein, M.: 825-59, 830-79, 1245, 2230, 4090, LB3620-388
Kagotho, N.: 2405-239
Kahana, B.: 890-218, 1400-258, 3370-289
Kahana, E.: 890-218, 1400-258
Kahana, E.: 1401-276
Kahana, E.: 3370-289, 4160
Kai, I.: 880-202, LB2570-444
Kail, B. L: 2280
Kaiser, B.: 3350-240
Kajander, M.: LB3620-317
Kajitani, S.: LB2570-440
Kallmyer, B.: 1160
Kalmijn, M.: 660
Kamat, A.: 835-93
Kamdar, N.: 1300-10, 2010, 2325-32, 3395
Kamesawa, A.: 925-319
Kamiya, Y.: 4065
Kammerer, C. M: 1310-42
Kampen, R.: 2270
Kamper, H.: 2385-173
Kan, C.: LB3620-415
Kane, A.: 820-36, LB1545-362, LB2570-323
Kane, C.: 3300-93
Kane, K.: 895-230
Kane, R. A: 3240
Kang, B.: 1105
Kang, C.: 820-33

Kang, G.: 1402-297, 2360-99, LB935-381
Kang, H.: 1315-53, 4080
Kang, H.: 1300-2, 1375-188
Kang, H.: LB2570-425
Kang, H.: 2360-82
Kang, J.: 2430-294
Kang, J.: LB1545-398
Kang, M.: LB1545-398
Kang, S.: LB1545-398
Kang, S.: 2395-216
Kang, S.: 3325-174
Kang, S.: 3370-299
Kang, Y.: 1315-57
Kanne, G. E: 1305-24
Kanyat, R.: 1380-201
Kao, D.: LB2570-321
Kaplan, A.: 2410-258
Kaplan, D. B: 1075
Kapogiannis, D.: 3295-71
Kapoor, A.: 3355-256
Karandikar, S.: 2405-239
Karasik, R. J: 735, 900-248, 2560
Karel, M. J: 2015, 3255
Karikari, G.: 900-241, 1370-182
Karikari-Martin, P.: 2385-184
Karim, H.: 825-45
Karim, H.: 2360-94
Karl, J.: 2420-270
Karmarkar, A.: 2385-171
Karnick, C.: 1365-174
Karpecki, P.: 1320-64
Kartin, D.: 855-152
Karus, D. G: 2430-300
Karvonen-Gutierrez, C.: 855-148, 2445
Kasahara, S.: 2390-199
Kaskie, B. P: 1100, 2470
Kasper, J. D: 1170, 3420
Katagiri, K.: 2425-278
Katagiri, K.: 3300-91
Katlic, M.: 2115
Katsumata, A.: 2320-16
Katz, A.: LB1545-392
Katz, A. D: 3300-101
Katz, B.: 3290-62
Katz, B.: 2500
Katz, E.: 3350-243, LB935-410
Katz, G. R: 2395-212
Katz, M.: 1170
Katz, M.: 845-124, 1175
Katz, R.: 4020
Katz, R.: 3140
Katz, S.: 580
Kaufman, B. G: 870-182
Kaufman, D.: 2325-30
Kaufman, J.: 4070
Kaufmann, C. N: 2125
Kaup, M.: 2515
Kauwe, G.: LB935-349
Kavanagh, K.: 835-87
Kawai, H.: 855-154, LB2570-411
Kaye, J.: 1401-282, 2060, 2405-241, 3535, 4165, LB2570-357
Kaye, L.: 910-257
Kayed, R.: 825-46
Kazis, L.: 740, 3535
Kazlauckas, N.: 1385-210
Kcomt, L.: 1000
Keefe, B.: 1390-224
Keefe, F.: 3365-275
Keenan, B. E: 2380-161
Keene, J.: 3330-191
Keepper, K.: LB3620-358
Kegler, M.: LB3620-325
Kehat, O.: 1402-293
Kehler, D. S: 4045
Kehoe, P.: 3320-140
Keibler, T.: 3240
Keiser, S.: 2215
Keleher, V.: LB3620-339
Kelemen, L.: 3305-108
Kelfve, S.: 865-178
Keller, A. O: 840-103
Keller, J.: 820-37
Keller, K.: 820-36
Kellett, J.: 705
Kellett, K. G: 1305-25
Kelley, J.: 1145
Kelley, S. J: 3245
Kelly, C. S: 1315-58
Kelly, C.: 1335-95
Kelly, C. M: 915-284, 3345-225, LB935-393
Kelly, K.: LB3620-362
Kelly, L.: 3345-212
Kelly, O. J: 1315-55
Kelly, P.: LB2570-340
Kelly, R.: 820-28
Kemeny, M. E: 505
Kemeny, M. E: 850-140
Kemnitz, J. W: LB3620-363
Kemp, B.: 720, LB2570-423
Kemp, C. L: 3270-9, LB3620-321
Kemp, N.: LB2570-328
Kempen, G.: 565, 3270-5
Kenaley, B. L: LB2570-442
Kennedy, A.: 890-222, 1075
Kennedy, B.: 4090
Kennedy, E.: 830-73
Kennedy, J.: 1402-295
Kennedy, K.: 2530
Kennedy, R. E: 1425, 2315-4, 3365-274, 3595
Kennedy, T.: 4155
Kennedy-Malone, L.: 2315-8
Kennedy-Malone, L. M: LB3620-421
Kenney, K. C: 2370-123
Kent, J.: LB935-350
Kent, M.: 2325-26
Kent de Grey, R.: 1315-48
Kenya, S.: 1402-301
Keogh, S.: 2105, 4055
Keohane, S. M: 3270-7
Kerns, R. D: 2370-119
Kerr, N.: 3345-218
Kerssens, C.: LB2570-415
Kertai, M. D: 2240
Kerwood, R.: 810-2
Keryl, M.: 3320-134
Kessler, V. L: 1305-18
Ketcham, C.: LB2570-412
Khadeer, M.: LB3620-364
Khaleghi, R.: LB1545-373
Khan, A. M: 600
Khan, C. P: LB935-416
Khan, M.: LB3620-375
Khan, N.: 2400-230
Khatib, D.: LB935-366, LB3620-326
Khatri, N.: 2400-222
Kheifets, V.: 825-43
Khiewchaum, R.: LB935-369
Khoo, Y.: 2010
Khosla, S.: 3455
Kiefe, C. I: 1400-260
Kiefer, L.: 3345-219
Kieffaber, P.: 3290-44
Kieffer, L. A: LB1545-432
Kiel, J. J: 1400-261
Kietzman, K. G: 3305-104
Kiflezghi, M.: 830-79
Kikutani, T.: 895-232
Kilaberia, T. R: 3305-106, LB2570-376
Kilgore, D.: 2120
Kilpela, L. S: LB2570-399
Kim, A.: LB3620-375
Kim, A.: 2410-246
Kim, B.: 3115
Kim, B.: 2215
Kim, B.: 1305-30, 1350-122, 3230
Kim, B.: LB3620-335
Kim, C.: 3325-168, LB2570-443
Kim, C.: 3325-174
Kim, D.: 1402-305
Kim, D. H: 3050, 3325-169
Kim, D.: LB2570-375
Kim, D.: 1350-119, 3340-198
Kim, E. S: 1425, 4100
Kim, E.: LB1545-352
Kim, E.: LB2570-442
Kim, E.: LB3620-324
Kim, E.: 1402-305
Kim, E.: 2355-80
Kim, G.: 1310-35, 1325-73, 1340-100, 1390-232, 3290-60
Kim, H.: 830-74
Kim, H.: 1375-188
Kim, H.: LB2570-411
Kim, H.: 710
Kim, H.: 3295-80, 3350-234
Kim, H.: 3360-267
Kim, H.: 3325-174
Kim, H.: 1415
Kim, J.: 1305-16
Kim, J.: 925-319
Kim, J. H: LB1545-413
Kim, J.: 1365-172
Kim, J.: 2420-276
Kim, J.: 1305-19
Kim, J.: LB3620-316
Kim, J.: 830-74
Kim, K.: 3325-168, LB2570-443
Kim, K.: 3325-174, LB1545-398
Kim, K.: 1340-100
Kim, K.: 2410-246
Kim, K.: LB935-422

Kim, K.: 2340-37, 3150, 3270-10, 3315-129
Kim, M.: 2380-156
Kim, M.: 925-327, 1390-234
Kim, M.: 2140, LB1545-419
Kim, M.: 2315-6
Kim, S.: LB1545-417
Kim, S.: LB1545-357
Kim, S. M: 1405-318, 4160
Kim, S.: LB935-359, LB2570-439
KIM, S.: 840-111
Kim, T.: LB2570-337
Kim, T.: 1365-178
Kim, W.: LB3620-415
Kim, Y. K: 2340-37
Kimata, M.: 3270-4
Kimberly, L. L: 885-212
Kimiecik, J.: LB2570-408
Kindratt, T.: 2355-64
King, B.: 2315-2
King, B.: 2410-262
King, B. M: 2550, 3480
King, G.: 1402-310
King, J. W: 2110, 2455
King, K.: 3125
King McLaughlin, J. B: 845-116
Kingston, A.: 1290, 1325-67, LB1545-418, LB2570-319
Kingston, A.: 685
Kingston, P.: 3490
Kinlaw, K.: LB2570-351
Kiosses, D.: 3220
KIP, K. E: 1330-93
Kirch, M.: 705, 2155
Kirchin, D.: 2225
Kirkland, S.: 565
Kirkland Caldwell, J. Z: LB935-404
Kishimoto, K.: 900-248
Kitamura, A.: 3340-204
Kitamura, S.: 895-225
Kitner-Triolo, M. H: 705
Kitzman, D.: 3325-175, LB2570-387
Kivnick, H. Q: 1185, 2145, 2200
Kiyoshi-Teo, H.: 1360-160
Klanchar, S.: 3320-137
Klaus, J.: 1330-81
Klein, E. M: 885-211
Kleineidam, L.: LB3620-318
Kler, S. E: 1305-33
Klima, D. W: 2360-101
Klimstra, M.: 2360-105
Klinger, C.: 905-255
Kloseck, M.: 2355-75, 3560
Klunk, W.: 825-45
Klusmann, V.: 3615
Kmetz, L.: LB3620-385
Knatterud, L.: 3240
Kneale, D.: LB935-418
Knepple Carney, A.: 1345-112
Knight, B. P: 855-149, 3375-311
Knight, B. G: 1315-49
Knight, E. L: 825-53
Knight, J. E: LB2570-347
Knoefel, F.: 3595
Knopman, D.: 3075
Knopp, K.: 2550
Knopp-Sihota, J. A: 3220
Knox, S.: 850-139, 4135
Ko, H.: 3230
Ko, P.: 3480
Kobayashi, E.: 3340-210, LB935-439
Kobayashi, L. C: 4110
Kobayashi, M.: 3315-130
Kochan, N. A: 655
Koehly, L. M: 925-331, LB3620-392
Koehn, S.: LB935-383
Koepp, R. M: 2015
Koeuth, S.: 885-207
Koffer, R. E: 3190
Kogan, L.: 1090
Kohlbacher, F.: 4150
Kolanowski, A. M: 2525
Kolodziejczak, K.: 4015
Kondash, M.: 810-2
Kondolf, K.: 2130
Kondrikov, D.: 835-100, LB3620-330
Konety, B.: 2380-155
Konetzka, R.: 910-258
Konetzka, R.: 2480, 3140
Kong, D.: 4105, LB2570-405, LB3620-428
Kong, J.: 1250, 1440
König, H.: 3320-139
Konnert, C.: 2340-40
Konola, T.: 3355-256
Kononova, A.: LB935-432
Konopka, A. R: 835-88
Konrad, T.: 780
Koon, L. M: 3500, LB1545-373
Kooner, P.: LB1545-412
Kopecek, M.: LB1545-333
Kopera-Frye, K.: 890-224, 1285
Koppel, S.: 1410-331
Korc-Grodzicki, B.: 635, 1365-174, 3275-20, 3305-105, 4110
Koren, C.: 3030
Kornadt, A. E: 2040, 3380, 3615
Koroukian, S.: 1395-243
Kostiuk, R.: 1390-224
Kotarba, J. A: LB935-372
Koumoutzis, A.: 4035
Kovac, C.: LB1545-373
Kovach, C.: 3220
Kovaleva, M. A: 840-107, 850-125, 2530
Kovalik, J.: 715
Kowal, P.: 2410-251, LB1545-342
Kozlov, E. K: 3270-6, 3310-119
Kraal, Z.: LB1545-444
Krabbe Pedersen, J.: LB2570-338
Kraemer, K.: 3295-79
Kraemer, S.: 1315-60, 1400-268, 2420-270, LB935-355
Kraft, R.: LB935-403
Kramer, J.: 3485, 3530
Kraus, V. B: 820-41
Kraus, V.: LB2570-394
Kravchenko, J.: 2240, 2370-132
Krefman, A.: 3280-34
Krekula, C.: 3000, 4125
Kremen, W.: 1425, LB2570-348
Kreps, G. L: 1401-276
Kresevic, D. M: 895-227, 915-293, 1320-63
Kressig, R. W: LB2570-367
Kridli, S.: LB1545-340
Krieger, N. I: 835-86
Kritchevsky, S.: 805, 2085, 3280-33, 3325-175, 3590, LB935-403, LB1545-387, LB1545-390, LB2570-387, LB3620-348
Krok-Schoen, J. L: 1315-55
Krook, M.: 1345-113
Krousel-Wood, M.: 1315-52
Krueger, S.: 1385-219
Kruger, T. M: 680, 735, 2440
Kruisbrink, M.: 565
Krus, G.: 915-283
Kruse, A.: 3375-310
Kuai, Z.: 825-42
Kube, E.: 2055
Kuchel, G. A: 3010, LB2570-393
Kuh, D.: 3170
Kuhn, T.: LB2570-335
Kuipers, A. L: 1310-42
Kuipers, A. L: 2370-136
Kuklova, M.: LB1545-333
Kulibert, D.: 2355-78
Kulkarni, A. S: 2530
Kullgren, J.: 705, 2155
Kulminski, A.: 820-33, 2370-125, LB1545-440
Kumar, A.: 830-68
Kumar, A.: 2545, 3395
Kumar, P.: 1310-37
Kunik, M. E: 2385-170, 3345-219, LB2570-390
Kunkel, S. R: 1305-18, 2385-183
Kunzel, C.: LB1545-374
Kuo, A.: 835-94
Kuo, C.: 1180, LB2570-393, LB3620-333
Kuo, N.: 2385-182
Kuo, P.: 2370-129, 3040, 3075, LB935-446
Kuo, T.: 2400-223
Kuo, Y.: 2155
Kuo, Y.: 850-135
Kuo, Y.: 2345-53, 2385-171, 3355-250, 4020, LB1545-427
Kupzyk, K.: LB2570-395
Kurth, M.: 815-13, 2055, 3340-209, 3370-301
Kurucz, S.: 2270
Kushalnagar, P.: 3595
Kusmaul, N.: 1480, 3065, 3345-233

Kuy, K.: 515
Kuzmik, A.: 2315-3
Kwak, J.: 3105, LB3620-385
Kwan, R.: 840-106
Kwoh, K.: 915-293
Kwok, S.: 3355-255
Kwok, W.: 2400-227
Kwon, E.: 840-111, LB1545-360
Kwon, E.: 2030
Kwon, S. C: 850-131
Kwon, S.: 3360-265

L

Labreche, T.: 850-142
Labunskyy, V.: 1115
Lach, H. W: 1020
Lachance, C.: 2380-161
Lachman, M. E: 700, 890-214, 915-285, 925-320, 2410-257
Lachs, D.: 2370-118
Lachs, M.: 2225, LB935-444
LaCroix, A. Z: 570, 1455
Laditka, J. N: 860-156, 1345-117, 2375-139
Laditka, S. B: 860-156, 1345-117, 2375-139
LaFave, S.: LB2570-353
LaFazia, D.: LB935-436
Laffer, A.: 500
Laffer, A.: 2410-250
Laflamme, E.: 1300-6
Lafontaine, C.: 580
Lafranchise, E. A: 1390-230
Lag, M. R: LB935-399
Lagacé, M.: 1300-5
Lagari, V.: 3355-255
Lagervall, J. A: LB935-399
Lagus, J.: 3535
Lai, A.: 1390-235
Lai, D.: 1515, 2030
Lai, Y.: 1401-288
Laico, A.: 920-310
Laird, R.: 1320-62
Laksari, K.: 820-26
Lam, B.: 1395-246
Lam, J.: 1385-215
Lam, J.: 2010
Lamar, M.: 3480
Lamarre-Cliche, M.: 3280-29
Lambert, D.: 1305-25
Lamming, D. W: 1520
Lampkin, C.: 2420-271
Lampraki, C.: 1385-213, 2340-49
Lan, T.: 3280-34
Lanai, B.: LB2570-418
Landes, S. D: 1445
Landry, G. J: 1510
Lane, D. W: 4140
Lane, K. R: 4005
Lane, K. R: LB1545-438, LB2570-398
Langa, K.: 2010
Langa, K.: 2325-29
Lange Maia, B. S: 1300-6, 2445
Langkilde, A.: LB2570-339
Langley, J.: 610
Lapane, K. L: LB935-435
Lapenskie, J.: 3415
Lapham, S.: 655
LaPierre, T.: LB2570-425
Lares, G.: 2495
Larkina, M.: 3340-202
Lasri, N.: 1402-293
Lassale, C.: 730
Latham, N.: 2210
Latham-Mintus, K.: 1150, 2410-261
Latimer, A.: 1340-99
Lau, D.: 910-268
Laude, T.: 730
Lauderdale, S. A: 1401-281
Lauer, S.: 855-150
Laurienti, P. J: LB935-403
Lautenschlager, N.: 915-290
Laux, J.: LB935-384
Lavacot, J.: 1330-84
Lavayen, S.: 895-231
Lavender, K.: 3340-197
Lawley, M.: 1095
Lawrence, C.: 2405-241
Lawrence, R.: LB1545-391
Laws, H.: 3315-127
Lawson, G.: 3330-190
Lazernick, B.: 3355-247
Le, O.: LB3620-352
Leach, C.: LB2570-326
Leach, C.: 2050
Leal, I.: 930-339
LeBlanc, M. R: LB2570-403
LeBlanc, R. G: 930-342, 1090
LeBlanc, R. G: LB1545-403, LB2570-372
LeBlanc, T. W: LB2570-403
LeBrasseur, N.: 830-67, 835-84, 2510, 3440
Lederman, S.: 2510
Lee, A.: 895-227
Lee, C.: 630
Lee, C.: 3180
Lee, C.: 1440, 3340-203, 4145
Lee, C.: 2410-246
Lee, D. J: 1395-246
Lee, D.: 3340-209
Lee, E.: LB2570-375
Lee, G.: 915-296, 3290-64, 3370-294, LB3620-429
Lee, G.: LB1545-419
Lee, H.: 3030
Lee, H.: LB2570-375
Lee, H. Y: 2140
Lee, H.: 3345-226
Lee, H.: 3325-169
Lee, H.: 2140
Lee, H.: 2430-294
Lee, H.: LB2570-432
Lee, H.: 925-336
Lee, H.: 3340-209, 3370-299
Lee, J.: 3420
Lee, J.: LB2570-410
Lee, J.: 3175
Lee, J.: 2235, 2280, 2365-110
Lee, J.: 3350-236
Lee, J.: LB2570-360
Lee, J.: 880-199
Lee, J.: LB3620-417
Lee, J.: 2215, 3325-168, LB2570-443
Lee, J.: LB1545-388
Lee, J.: 3010
Lee, J.: LB3620-337
Lee, J.: 3320-140
Lee, J.: 3295-69
Lee, K.: 910-278
Lee, K.: 1300-4
Lee, K.: 1365-173
Lee, K.: 3315-126, 3340-198, 3370-294
Lee, K.: LB3620-315
Lee, K.: 2215
Lee, K.: 3325-168
Lee, K.: LB2570-337
Lee, L.: 1305-32
Lee, L. O: 1130, 4100
Lee, L.: 1315-56
Lee, L.: 2410-260
Lee, M.: 3520
Lee, O. E: 4160
Lee, P.: 4005
Lee, S.: 3370-297
Lee, S.: 825-61
Lee, S.: 4045
Lee, S.: 635
Lee, S.: 3300-96
Lee, S.: 880-197
Lee, S. R: 1400-266
Lee, S.: 3275-21, 3580, LB2570-362, LB3620-327
Lee, S.: 3340-208
Lee, Y.: LB2570-432
Lee, Y.: LB2570-374
Lee, Y.: 1300-4, 2405-245
Lee, Y.: 1305-23
Lee, Y.: 1400-267
Lee, Y.: 2340-42
Lee, Y.: 1350-126, 2375-138
Lee, Y.: 1395-242
Lee, Y.: 1395-241
Leech, A. L: 1225
Leedahl, S. N: 905-253, LB2570-422
Lees-Haggerty, K.: 795
Leeth, E.: LB3620-336
Lefler, L. L: 920-311
Légaré, F.: LB1545-441
Legault, V.: 4005
Leger, K. A: 3425
Leggett, A. N: 3110
Lehning, A.: 745, LB2570-434, LB3620-353
Leinenweber, M.: 1350-123
Leiser, S.: 2085, 3385
Leist, A. K: 1400-263
Leite, R. O: 3315-130
Leiter, R. E: 870-181
Lekan, D. A: 2315-8, LB2570-331
Lekhak, N.: 895-239
Lemke, A. L: LB2570-430
Lemmen, T.: LB2570-421

Lenchik, L.: LB1545-390
Lendon, J. P: 910-268
Leng, I.: 805
Leng, S. X: 3280-32
Lenox, M. B: 1315-45, 1330-82
Lensing, S. Y: 920-311
Leon, T.: 2365-109
Leonard, N. M: LB2570-435, LB3620-378
Lepore, M. J: 740
Leritz, E.: 3290-58
Lesandrini, J.: LB3620-334
Leslie, M.: LB2570-422
Lesser, J.: LB1545-384
Lette, M.: 635
Leung, A.: 840-106, 1345-115, 2060
Leung, D.: 2400-227, 2425-287, LB3620-440
Leung, D. L: 2230, 4005
Leung, D.: 3245
Leutwyler, H. C: 1330-91
Leveille, S.: 565, 3220
Levine, M. E: 625, 1400-262, 2230, 2260, 3040, 3440, LB1545-422, LB2570-429, LB3620-333
Levins, T.: LB2570-398
Levis, S.: 3355-255
Levy, B.: 2225
Levy, C.: 1402-314, 4135
Levy, L.: 3500
Levy, S.: 3370-295
Lewinson, T.: 4060
Lewis, C.: LB3620-338
Lewis, J. P: 1405-318, 1425, 2405-243, 4160
Lewis, N. A: LB2570-347
Li, B.: 820-40
Li, C.: 1515
Li, C.: 835-94
Li, C.: 885-210
Li, C.: 1330-89
Li, C.: 1040
Li, C.: 2385-171
Li, D.: 1330-89
Li, F.: LB3620-397
Li, G.: 3325-171
Li, H.: 3350-241
Li, H.: 805
Li, H.: 3280-32
Li, J.: 1515
Li, J.: 2415-267
Li, J.: 3200
Li, J.: 885-210
Li, J.: 920-305
LI, J.: 920-305
Li, J.: 2420-276
Li, L.: 1510
Li, L.: 2030
Li, L.: 855-151, 1350-121, 2400-229, 3275-25
Li, M.: 615, 2010, 3505, LB2570-433
Li, M.: 830-65
Li, M.: 1390-225
Li, M.: 3395, 4160
Li, M.: 3325-176
Li, P.: 845-120
Li, Q.: LB1545-376
Li, Q.: LB935-380
Li, Q.: 855-149, 3375-311
Li, S. z: 4030
Li, W.: 660
Li, W.: 895-230, 1070, 1500
Li, X.: 2313
Li, X.: LB1545-388
Li, Y.: 860-155
Li, Y.: 2375-146
Li, Y.: 1390-235
Li, Y.: LB1545-380
Li, Y.: 810-1, 1297
Li, Y.: 3290-51Lim, M. A
Liang, H.: 835-91
Liang, J.: 1305-30, 1350-122, 3230, LB935-439
Liang, J.: 3320-138
Liang, S.: LB3620-437
Liao, H.: 925-323, 1385-218
Libermann, T. A: 825-48, 4130
Liberty, A.: 2315-7Lichtenberg, P.
Liddle, J.: 850-130
Liddle, J.: 2395-213
Liebzeit, D.: 2315-2
Liehr, P.: 3325-170
Liepert, C. R: LB2570-424
Ligus, K.: 2430-297
Lim, E.: 740, 915-288, 2310
Lim, S.: 850-131
Lim, S.: 3335-196
Lima, J.: 820-34
Lima, J. C: 740
Lima, K.: 860-158
Lima, N.: LB2570-341
Lin, C.: 3420
Lin, C.: 3400
Lin, F.: 2380-166, 2425-291, 2495, 4155
Lin, H.: 3335-195, 3365-283
Lin, I.: 4145
Lin, J.: 925-331
Lin, J.: 905-250
Lin, L.: 845-122
Lin, M.: 3400
Lin, S.: 910-267
Lin, S.: 870-184, LB935-401, LB1545-358
Lin, X.: 925-320, 1060
Lin, Y.: 4050
Lin, Y.: 895-234
Lin, Z.: LB1545-379
Linck, J.: 3350-239
Lind, M.: 4095
Lindauer, A.: 840-104, LB935-436, LB1545-430
Lindberg, B. W: 695, 1230, 1530, 2085, 2285, 2460, 3135, 3515
Lindeman, C.: 850-126
Lindeman, D.: 2295
Linden, W.: LB3620-383
Lindsey, J.: 1195
Liou, C.: 2000, 2390-204
Liou, S.: 3460
Lippe, M. E: 900-243
Lipsitz, L.: 1402-302, 3130
Lipson, A. R: 710
Lipton, R. B: LB3620-318
Litvinchuk, A.: LB935-364
Litzelman, K.: 1355-135
Liu, A.: 3175
Liu, C.: 2270
Liu, C.: 1310-37
Liu, D.: 1040, 1325-73, 1400-259, 3330-191
Liu, F.: LB935-446
Liu, H. J: 4130
Liu, H.: 2340-43
Liu, H.: 1355-137
Liu, J.: 1401-288
Liu, J.: 3120
Liu, J.: 2400-229, 3320-142, 3330-186
Liu, K.: 3280-34
Liu, L.: 2325-30
Liu, M.: 2475
Liu, M.: 920-305, 1105, 1510, LB2570-353
Liu, M.: LB3620-433
Liu, P.: LB935-377
Liu, S.: LB1545-377
Liu, S.: 3090, 3160
Liu, T. T: LB1545-404
Liu, T.: 915-294, 2400-227, 2425-287, LB1545-368, LB3620-440
Liu, T.: 2395-216
Liu, T.: LB2570-327
Liu, W.: 2355-74
Liu, X.: 2400-225
Liu, X.: 3290-56
Liu, X.: 805
Liu, Y.: 3340-201
Liu, Y.: 2313
Liu, Y.: 820-35, 830-70, 835-97
Liu, Y.: 890-214, 915-285
Liu, Z.: 1400-262, 2230, 3325-170, LB3620-333
Liu-Ambrose, T.: 565, 715, 920-313, 1510, 2215, 2360-90, 3280-36, 3295-72
Livingston, D.: 930-341
Ljungman, P.: 850-137
Lloyd, D.: 3320-138
Lloyd, D. A: LB3620-444
Lo, H.: 1330-85
Lo, O.: 1402-302, 4020
LoBuono, D. L: LB2570-422
Locher, J.: 1425, 2070, 3365-274
Lockhart, S.: LB2570-401
Lodi-Smith, J.: 585
Loeckenhoff, C.: LB1545-341
Loeser, R. F: 715
Loew, D.: 2365-107
Loewenstein, D.: 1055
Lofgren, I. E: LB2570-422
Loggia, G.: 825-49
Loh, K.: 855-146
Loh, L.: 3540
Lohman, M.: 3325-164, 3600, LB1545-364
Lohr, s.: 825-43
Lohrmann, D. K: 900-241, 1370-182
Loi, S.: 915-290
Loika, Y.: 820-33
Lokanathan, T.: LB2570-344
Loomer, l.: 3555
Looper, M.: 920-315

Lopez, O.: 3595, 4045
Lopez, T.: 1305-25
Lopez, V.: LB935-431
López-Ortega, M.: 750
Lor, M.: 775
Lord, J. C: 910-262, 2185, 2395-218, 3345-213, 3435
Lorenz, K.: 3535
Losinski, G.: 500, 2410-250
Loter, K.: 3200
Lothary, A. F: 2340-34, 4095
Lou, V. W: 1345-115, 1355-137, 2000, 3250
Lou, Y.: 1340-104, 3320-142, 3330-186
Loup, J.: 1401-286, 2535, LB3620-351
Louras, P.: 2390-196
Lourenço, M.: 1390-222
Lovett, J.: LB3620-364
Lowder, Y. P: 2345-56
Lowenstein, A.: LB935-440
Loza, R.: 2380-157
Lu, A. T: 625, 3405
Lu, J.: 1297
Lu, M.: 3605
Lu, N.: 1340-103, 1435, 3250, 3270-10
Lu, P.: 4145
Lu, S.: 2400-227, 2425-287, LB1545-368, LB3620-440
Lu, Y.: 3605
Lu, Z.: 845-117Lubieniecki, K.
Lucas, J. A: 3555
Lucas, L.: 855-146
Lucero, R.: 1402-294, 2370-120
Luchsinger, J.: 2495
Luci, K.: 1355-145
Ludlow, N.: 880-199
Ludwig, R.: 585
Luetke-Stahlman, H.: 2325-25
Luft, B.: 825-52
Lugo, I.: 3315-130
Lui, K.: LB2570-412
Lukens, E.: 885-212, 3070, 3180
Luk-Jones, S.: LB2570-414
Lukkahatai, N.: 920-305
Lum, H. D: 1335-97, 2385-173
Lum, T.: 845-121, 1345-115, 2400-227, 2425-287, LB1545-368, LB3620-440
Luna, F.: 3320-143
Lundy, J.: 1020
Luo, H.: 3075
Luo, J.: 3330-185
Luo, Y.: 1401-270
Luo, Y.: LB2570-361
Luo, Z.: 3305-109
Luong, G.: 3425
Luppi, F.: 1515
Luster, J.: 540
Lustig, E.: LB2570-373
Luszcz, M. A: LB1545-445
Luth, E. A: 1160, 3310-115, 4010
Luthar, S.: LB2570-402
Luther, S.: 2370-119
Lutz, J.: 3350-243, 4140
Luxenberg, J.: 2010
Luz, C.: 3305-109
Luz, C. C: 3510, LB2570-410
Ly, M.: 825-45
Ly, T. K: LB2570-335
Lydon, E.: LB2570-415
Lyles, M.: LB935-403
Lynch, S.: 805
Lynn, J.: LB3620-326
Lyon, R.: 3355-247
Lyons, D. L: LB935-391
Lyons, K.: 1350-120, LB2570-359
Lyons, M. J: LB2570-348, LB3620-367
Lytle, A.: 4080
Lyytinen, K.: 1401-276

### M

M Khan, K.: 565
Ma, C.: 1400-262
Ma, C.: 2380-165
Ma, F.: 3285-37
Ma, F.: 1401-286, 2390-196
Ma, M.: 2115
Ma, R.: 2395-219
Macaluso, S.: 875-188
MacArthur, M.: LB1545-362, LB2570-323
MacCarthy, S.: 3430
MacDonald, S.: 2360-105, 3470
Macedo, T.: 3180
Machanda, Z.: LB1545-431
Machu, V.: LB1545-333
Maciel, A.: 3365-281
Mack, D. S: LB935-435
Mack, E.: LB3620-343
MacKenzie, B.: 895-231
Mackey, D.: 2380-161
Mack-Minniefield, R.: 1305-24
Maclagan, L. C: 555
MacNamara, M.: 1090
MacPherson, A.: 1230, 3135
Macro, J.: 830-65
Madden, K.: 565, 920-300
Madden, K.: LB3620-383
Mader, M. J: LB1545-366
Madero, E.: 1402-300
Madigan, J.: 2355-80
Madjaroff, G.: 1401-280
Madole, J.: 1430
Madrigal, C.: LB1545-397, LB2570-329
Maercker, A.: 1330-88
Magaziner, J. S: 820-21, 2210, 4130
Magda, S.: 3320-137
Magid, K.: 4135
Magnottta, V.: LB935-350
Magruder, K.: 2365-111
Mahjabin, J. E: LB3620-338
Mahlo, L.: LB935-387
Mahmoud, K. O: 3320-144
Mahmoudi, E.: 1300-10, 2010, 2325-32, 2545, 3395
Mahoney, E. K: 1235
Mahoney, J.: LB935-422
Mahoney, J. R: 2360-84
Mahoney, K. J: 1235
Maiden, R. J: 2100, 2500
Mailick, M. R: 2375-148
Maillet, D.: LB1545-441
Mair, C.: 745, 2550
Maisano, J.: 3220
Mak, W.: LB3620-393
Makino, M.: LB1545-344, LB2570-320
Malani, P.: 705, 2155
Malatyali, A.: 3295-75
Maleku, A.: 2405-239
Malhotra, R.: 865-167, 2310, 3355-251
Malhotra, V.: 3305-105
Maller, M.: LB3620-422
Mallya, J. U: 2360-96
Malmstrom, T. K: 3350-235
Maloch, E.: LB1545-350
Maloof, M.: 2215
Malta, S.: 915-290
Mama, S. K: 2050
Manalel, J.: LB3620-392
Manda, P.: LB2570-331
Mandi, D.: 2495
Maneix, L.: LB935-373
Manfredi, G.: 3325-158
Mangieri, D. A: LB2570-327
Manheim, C.: 4135
Manini, T. M: 1402-296, 2410-264, LB2570-386, LB3620-403
Manly, J.: LB1545-410
Manna, R.: 635, 1365-174, 3275-20, 3305-105, 4110
Mannick, J.: 4090
Manning, B.: LB1545-362
Manning, C.: 2355-67
Manning, L. K: 1315-44, 3370-285
Manoogian, M. M: 1285, 1350-123, 2560
Manor, B.: 530, 1402-302, 4020, LB3620-328
Manrique Espinoza, B.: 3325-154
Mansfield, J.: 1390-230
Manson, J. E: LB2570-441
Mantantzis, K.: 2275
Manthorpe, J.: 850-138
Manuel-Apolinar, L.: 1310-40
Mao, W.: 615, 1435
Marcantonio, E. R: 825-48, 4130
Marceaux, J. C: 3295-76
Marcelli, C.: 825-49
Marchese, C.: 1410-329
Marcinek, D.: 835-101
Marcione, N.: 920-303

Marcoe, J.: 1401-282
Marcotte, L.: 3280-36
Marcucio, R.: 820-19
Marcum, C. S: 925-331
Marcum, Z. A: 565
Marcus, E.: 2315-9
Marcus, K.: 900-245
Marcus, M.: 3610
Mardini, M. M: 1402-296, 2410-264
Marello, M. M: 890-221, 925-334
Marengoni, A.: 3170, 3325-173
Margot-Cattin, I.: 3565
Margrett, J.: 1270, 2390-202, LB1545-385
Marin-Cachu, A.: 3390
Marini, C. M: 2420-275
Marini, C.: 3320-146
Marino, V. R: 1355-136, 2435-311
Mark, J.: 2555
Mark, J. A: LB935-423
Markey, C. H: 1315-58
Markides, K.: 750, 850-135, 1360-158, 3360-271
Markle-Reid, M.: 1395-245, LB1545-336
Marks, T.: 2385-187, 2400-226
Markt, S.: 1395-243
Markwardt, S.: 1395-248
Markway, B.: LB2570-398
Marlow, N. M: 3330-184
Marmstål Hammar, L.: 3295-86
Marolf, A. J: 835-88
Marota, R.: LB1545-354
Marquard, J.: LB2570-372
Marques, E.: 1402-307
Marques, I. G: 3360-266
Marquez, D. X: 2155, 3360-266
Marras, C.: 555
Marron, M. M: 1425, 3275-24, LB2570-437, LB3620-348
Marrs, S.: 4170
Marsack-Topolewski, C. N: 2505
Marsh, J.: 1315-52
Marshall, G.: LB935-419
Marshall, S.: 1410-331, 2550
Marsiske, M.: 2265
Marszalek, J.: 2340-36
Martie, L.: 3520
Martin, J.: 2395-215, 2420-273
Martin, J. G: 1365-171
Martin, K. J: 1401-281
Martin, P.: 915-296, 925-322, 1350-119, 2440, 3290-64, LB3620-389
Martin, R.: 2315-14
Martinez, L.: 905-255
Martinez, M.: LB1545-386
Martinez, R.: 3105
Martínez, J.: 890-213
Martino, S.: 2250
Martin-Plank, L.: 3120
Martire, L.: 2420-275, 3320-146, LB3620-381
Marty, M.: 3370-291
Marumoto, M.: 2000
Marx, K. A: 885-207, 1005
Maryanna, K.: 845-117
Masinda, S.: LB2570-342
Masliah, E.: 2290
Maslow, K.: 1295, LB3620-362
Mason, K. R: 2270, 3105, 3310-112
Mason, T.: 1330-93
Massimo, L. M: 605
Massimo, L.: 850-136
Mast, B. T: 845-119
Mastergeorge, A.: LB2570-445
Masternak, M.: 835-83, 4175
Masters, J.: 3270-1
Masterson Creber, R.: 4010
Matchar, D.: 715, 3090
Mateus, C.: 2310
Mathews, M.: 2250
Mathews, S. D: 3005
Matook, D.: 1402-295
Matsuda, Y.: 1305-23
Matsunobu, K.: 1390-229
Matsuo, H.: 2010, 2405-238, 3350-235
Matsuura, S.: 1390-220
Mattek, N.: 1401-282, 3535, LB935-436, LB1545-430
Matters, L.: LB3620-349
Matthews, F.: 1200
Matthews, F. E: 1325-67, 2370-134, LB2570-319
Mattos, M.: 2355-67
Matuszek, G.: 3280-33
Mauldin, R. L: 910-278, 1305-15, LB2570-321
Maust, D.: 2155
Mavandadi, S.: 1330-81
Mavroeidi, A.: 730
Mawhorter, S.: 1345-106
Maxey, M.: 3340-201
Maxfield, M.: 2355-68
Maxwell, C. J: 555, 3295-87
Maxwell, D.: LB2570-321
Mayas, j.: 3370-290
Maydan, G.: 710
Mayfield, K. E: 3245
Mayo, D.: LB1545-421
Mays, A. M: 4030
Mazor, K.: 3355-256
Mazula, D.: 835-84
Mazumder, H.: 2400-230
Mazzola, E.: 870-181
Mazzola, P.: 820-31
Mazzone, J. A: 2390-196
Mc Gee, S.: 1330-88
McAdams-DeMarco, M.: 2380-168, 4020
McAiney, C.: 1395-245, LB1545-336
McAlindon, T. E: 715
McAtee, R. E: LB935-420
McCall, G. S: 3290-56
McCarron, H. R: 1385-210, 2355-71
McCarthy, M. J: 1350-120
McCarthy, S.: 3025
McCaskill, G.: 2410-260
McCaughey, D.: 880-199
McCauley, B.: LB2570-352
McCauley, B.: LB3620-384
McCollister, K.: 1395-246
McConeghy, K.: 3255, 4130
McConeghy, R.: LB2570-329
McConnell, E. S: 520, 780
McConnell, E.: LB2570-385, LB3620-349
McConnell, W. R: 2550
McCormick, M.: 3195
McCoy, T.: 2315-8, LB2570-331
McCrae, C.: 670
McCreedy, E.: 705, 3240, 3555
McCrillis, E.: LB3620-373
McCulloch, T.: 920-315
McDannold, S.: 740, 3535
McDarby, M.: 3270-6, 3310-119
McDavid, J.: LB3620-352
Mcdermid, R.: 820-28
McDermott, M.: LB2570-386
McDevitt, K.: 905-252
McDonald, A.: 3330-189
McDonald, L.: 905-255
McDonough, I. M: 3295-79
McDougall, G. J: 3295-79
McDowd, J.: 1402-310, 2340-36
McDuffie, D. L: 2405-231
McEvoy, L.: 1455, LB2570-348
McFadden, S.: 2355-78
McFall, G.: 1360-162
McGarry, B. E: 1375-185
McGee, J. S: 2425-289, 3560
mcGee-Lawrence, M.: 830-73, 835-100, LB3620-330
McGhan, G.: 840-112, 880-199
McGilton, K.: 850-142, 3345-216, 3415
McGinley, J. M: 880-195, 2385-181
McGlinchey, R.: 3290-58
McGory Russell, M.: 2115
McGough, E.: 855-152
McGovern, E.: 3415
McGrew, K.: 3305-110
McGuinness, C.: 2340-40
McGuire, L. C: 1030, 1400-264, 1505, 4110
McGuire, L. C: LB935-362
McIlveen, H. A: 2355-75
McIlwain, A. S: 900-245
McIntosh, T.: 1515, 2355-62, 2430-296, 3140
McIntyre, M.: 4170
McKay, H.: 855-147
McKay, I. T: 3315-120, LB3620-324
McKay, M. A: LB1545-369

McKee, M.: 2010
McKenzie, C.: LB2570-440
McKenzie, G.: LB935-436
McKibbin, C.: 870-186, 1365-168, 2075
McKinney, R. E: 900-243
McKneely, K.: 1345-116
McKoy Davis, J. G: 3045
McKune, A. J: 705
McLane, P.: 4130
McLaughlin, S. J: LB935-402
McLin, C. L: 2340-50
McLinden, D.: 2225
Mclive-Reed, K.: LB2570-442
McMahan, E. A: 2310
McMahon, C.: 4155
McMahon, E. E: 2315-6
McMahon, M.: 2310
McManus, D. D: 1400-260
McMullen, T.: 2385-190
McNair, B.: LB1545-402, LB2570-346
McNally, J.: 3340-197
McNamara, S.: 1190
McNeil, J.: 870-180, 3260
McNeill, L.: LB935-436
McNiel, J.: 3325-164
McPhee, G. M: 3295-81
McPherson, B.: 4155
McPherson, M.: LB3620-355
McPhillips, M. V: 2420-276
McSweeney-Feld, M. H: 2065
Mdaki, K. S: 830-70
Meador, L.: 3220
Meekanon, P.: 3350-235
Meekes, W.: 730
Meeks, S.: 845-119, 1190, 2390-195, 3310-117, 3360-258
Meer, M.: LB3620-407
Meghani, S. H: 840-109
Mehegan, L.: 2340-39
Mehlsen, M.: 1385-205
Mehr, D.: 4135
Mehri, N.: 510
Mehta, H.: LB935-359, LB2570-439
Mehta, H. B: 3355-250
Mehta, N.: 915-286
Meinertz, N. R: 725, 2365-110
Meinhardt, A. J: 1240
Meinow, B.: 3420
Meister, L.: 3340-202
Mejia, A.: LB2570-365
Mejia, S. T: 550, 1350-125, 3190, LB3620-331
Melekin, A.: 2395-214
Melekis, K. A: 2320-20
Meling, M.: LB3620-317
Melipillán, R.: LB935-409
Melzer, D.: 1180, LB2570-393
Mendenhall, A.: 830-78, 3195
Mendes de Leon, C.: 925-337
Mendes de Leon, C. F: 3245
Mendez, J.: 1365-166
Mendez-Luck, C.: 2405-237, 3370-300
Mendialdua Canales, D.: LB935-430
Mendiratta, P.: LB3620-376
Mendoza, N.: 3235, 3320-150
Mendoza Ruvalcaba, N.: 3290-67
Menezes, J. M: LB1545-437
Meng, H.: LB3620-327
Mengel-From, J.: 1310-42
Menne, H. L: LB3620-323
Menon, V.: LB935-345
Meraz, R.: 3560
Mercado, C.: LB935-420
Merdjanoff, A.: 2540
Merrilees, J.: LB2570-336
Mertz, K.: 820-30
Meshoulam, I.: 1055
Messecar, D.: LB1545-430
Messi, M.: 820-25
Messier, S.: 3325-175
Metti, A.: 3075
Metzelthin, S.: 2380-164
Metzger, A.: 3350-243
Meucci, M. R: 1170
Meuser, T. M: LB1545-421
Meyer, K.: 1355-150, 2375-144, 2465, 3375-313
Meyers, K.: 3330-183, LB2570-366
Meyers, M.: 885-204
Meystre, C.: 2340-49, 3315-123
Mezuk, B.: 2410-252
Mi, H.: LB3620-319
Miao, H.: 1402-313
Michalek, J.: 3325-160
Michalowski, E.: 2435-307
Miclau, T.: 820-19
Micoli, T.: 2155
Midão, L.: 1402-307, 3325-158
Middlemass, K.: LB2570-399
Middleton, A.: 850-139, 4135
Mielcarski, J.: LB2570-438
Mielenz, T. J: 3325-171
Mielke, M.: 3075
Miescier, L.: 2385-184
Migliaccio, J. N: 3610
Mikkelsen, M. B: 1385-205
Milanez, M.: 2495
Milani, S. A: 3220
Milberg, W.: 3290-58
Miles, T.: 1000, LB2570-370
Miljkovic, I.: 2370-125
Miljkovic, I.: 2370-136, LB3620-348
Millar, R. J: 1325-71
Millar, R. J: 1345-118
Millar, R. J: 1400-259, 1401-290, 3300-99
Miller, B.: LB2570-439
Miller, E. A: 910-261, 1065, 1095, 2385-180, 3280-35, 3555, 3570, 4050
Miller, J.: 3555
Miller, J.: LB3620-341
Miller, J.: 835-84
Miller, J.: 660, 1375-184, 3180
Miller, K.: 1150
Miller, L.: LB3620-389
Miller, L.: 3595, 4045
Miller, L.: 2380-169
Miller, L. M: 3535, LB2570-357
Miller, M.: LB1545-364
Miller, M. G: 2380-167
Miller, N.: 3330-188
Miller, S.: 3345-224
Miller, S. C: 740
Miller, T.: 2435-312
Miller, V. J: 1480
Millett, G.: 820-20
Milligan, C.: 2310, 2430-301
Mills, W. L: 910-275, 3345-219
Milosevic, M.: 1402-306
Min, J.: LB3620-315
Min, L.: 4130
Min, S. N: 1400-252
Minahan, J.: 2080
Minahan, J.: 655, 3290-49
Minami, S.: 825-43
Minami, S.: 3270-4
Mingo, C. A: 770, 1160
Minkman, M.: 1365-176
Minster, R.: 1310-42
Minton, D. M: 835-88
Mion, L.: 845-117
Mireles-Cabodevila, E.: 2315-13
Mirelman, A.: 1402-304
Mirza, R. M: 905-255
Mishra, S.: LB2570-387
Mitchel, J.: 3275-22
Mitchell, G.: 850-130
Mitchell, J.: LB1545-362, LB2570-323
Mitchell, L. L: 1390-236, 1401-278, 2325-21
Mitchell, M.: 2395-215, 2420-273
Mitchell, S.: LB1545-362, LB2570-323
Mitchell, S.: 3555, LB1545-389
Mitchell, U.: 2280
Mitnitski, A.: 820-23, 825-55, 3325-167
Mitoraj, M.: 3275-19
Mittelman, M.: 2155
Mittlieder, C.: 895-236
Mitzner, T. L: 3500
Miyawaki, C. E: 1025, 2000, 3115
Mize, D.: 895-229
Mizuno, A.: 825-45
Moaddel, R.: LB3620-364
Mobley, T.: 835-100
Mochel, A.: 3345-219
Modrek, S.: 4065
Mody, L.: 4130

Moeke-Maxwell, T. H: 2270, 3105, 3310-112
Moellering, D. R: LB2570-371
Moen, P.: 4065
Moffat, S.: 1270
Moffatt, E.: 1325-67
Moffitt, T.: LB1545-347
Mogle, J.: 1175, 3290-52
Mogle, J.: LB1545-397
Mohaimin, S.: 850-131
Mohanty, S.: LB2570-331
Mohler, J.: 820-26
Mohr, D.: 3255
Mois, G.: 1401-287
Mois, G.: LB3620-329
Mojadam, A.: 2315-6
Mokhtari, B. K: 815-16, 2425-288
Molenaar, P.: 3295-69
Molhman, J.: 1401-281
Molnar, L. J: 1410-331, 3325-171
Molony, J.: 3305-108
Molony, S. L: 2315-5
Molton, I.: 855-148
Monaco, W.: LB3620-327
Mondadori, R.: 835-83
Monin, J. K: 815-17, 3315-127, LB2570-392
Moninger, S.: 1402-308
Monroe, T.: 845-117
Monserud, M.: 1150
Montano, A. L: 1365-169
Monteiro, A.: 930-339
Montemuro-Rode, L.: 1340-99
Montepare, J. M: 1420, 2220, 2560
Montero-Odasso, M.: 2095
Monti, S.: 835-92, 3215
Montoro-Rodriguez, J.: 1460
Montoya, Y.: 2155, 3360-269
Moo, L.: 1295, 2055
Moody, M.: 1315-47
Moon, H.: 2355-69, 3360-267
Mooney, A.: 865-166
Moore, A.: 1330-76
Moore, A.: 4050
Moore, C. S: 2395-212
Moore, J. M: 2340-44, 2425-288
Moore, K. J: 915-290
Moore, S. E: 1330-75, 1395-243
Moore, S. C: 1425
Moore, T.: 2395-212
Moore, Z. D: 1380-194
Moored, K.: 4095
Moorman, S. M: 720, 2235
Moorthi, R.: 820-18, 2410-261
Mor, V.: 705, 3140, 3555, 4130
Morales, X.: 2495
Moran, D.: 3545
Morani, A.: LB1545-361
Morano, C.: 880-192
Morehouse, S.: 1380-192
Morello, R.: 825-49
Moreno, G.: 2380-157, LB3620-408
Morey, M. C: 1330-83, 2345-56, LB1545-347
Morgan, A.: 3330-190
Morgan, B.: 870-179
Morgan, J. C: 2305, LB3620-321
Morgan, L.: 2395-219
Morgan, T. E: 825-50, 830-64
Morhardt, D.: 710, 2155
Mori, C.: 2340-40
Morita, H.: LB1545-344
Moro, T.: LB3620-345
Morris, D. L: 875-187
Morris, D. L: 3510
Morris, E. P: LB1545-434
Morris, J.: 3175
Morrow-Howell, N.: 845-118, 1420, 2030, 3235, 3395
Morthland, M.: 2310
Morton, P. M: LB2570-423
Moryl, N.: 3305-105
Moscou-Jackson, G.: 3140, LB935-416
Mosier, A.: 3375-306
Mosley, T.: 2495, LB2570-401
Moss, K. O: 3100
Moss, M. S: 1185
Mota, M.: 895-235
Mota, T. M: 2395-212
Motel-Klingebiel, A.: 865-178
Mouchawar, A.: 840-105
Moulder, R.: 2005
Mouton, P. R: 1310-39
Moxley, J.: LB935-417
Moye, J.: LB2570-409
Moyle, W.: 505, 1405-322
Moyo, P.: 4130
Mroczek, D. K: 2110, 3340-206
Mroz, T.: 855-148
Mrug, S.: 1360-154
Mtsunaga, H.: 895-226
Mudar, R. A: LB2570-415
Mudrazija, S.: 880-191
Mudzonga, C.: 860-160
Muehlbauer, M. J: LB2570-437
Mueller, C.: 1470, 4050
Mueller, C.: 2410-263, 3535, 4120
Muggy, T.: 2370-135
Mui, A.: 1405-316, 3320-142
Mukamal, K.: 3595
Mukamal, K. J: LB2570-389
Mulasso, A.: 3325-159
Mulcahy, J.: 1170, 3420
Mulhorn, K. A: 3375-314
Mullen, N.: 2550
Mullen, S.: 3350-240
Muller, M.: LB1545-431
Mullins, R.: 3295-71
Muma, A.: 3330-179
Mun, J.: LB935-374
Mundy, S.: LB3620-370
Muniz, P.: 1390-222
Muniz Terrera, G.: 3210, LB2570-347, LB3620-318
Munkhtogoo, D.: 2385-182
Munly, K.: 1445, 3465
Munoz, b.: 3295-73
Munoz, E.: 925-338
Munoz, L.: 895-231
Munshi, M.: 3050
Muppidi, N.: 825-45
Murabito, J.: 1525, 3405, LB2570-396
Murakami, K.: LB1545-396
Muralidhar, K.: 1320-61, 1330-94, 1402-299, 2350-59
Muralidharan, A.: LB1545-371
Muramatsu, N.: 1095
Murata, W.: 830-63
Murawski, P.: 855-150
Murayama, H.: 885-205
Murayama, H.: 3270-4
Murfin, K.: LB1545-337
Murphy, E. R: LB3620-405
Murphy, I.: 1305-28
Murphy, J.: 2345-58
Murphy, R.: LB3620-383
Murphy, T. E: 3315-127
Murphy, T. K: 3275-15
Murray, A. M: 870-180, 3260
Murray, K.: 3345-212, 3435
Murray, L. A: 2390-205
Murray, L. M: LB3620-436
Muruthi, J.: 3480, LB1545-342
Musi, N.: 820-39, 830-76, 835-91, 1310-41, 3325-160, LB2570-399
Musich, S.: 1315-60, 1400-268, 2420-270, LB935-355
Musil, C. M: 890-216, 2385-177, 3320-141, 1350-129, 1460
Mutchie, H. L: 2370-122
Mutchler, J.: 860-155, LB3620-359
Muzaale, A.: 2380-168
Muzzy, W.: 1055, 2225, 2365-107
Myers, D. R: 2425-289
Myers, D.: LB2570-415
Myers, L.: LB1545-417

### N

Naar, J.: 2435-314, 3315-128, 3340-200
Nabholz, L.: 2365-106
Nabors, E.: 3270-3
Nace, D.: 3390
Nadash, P.: 910-266, 1405-323, 2385-180, 3280-35, 3570, 4050

Nadorff, D. K: 2200, 3315-120, 3320-148, LB3620-324
Nadorff, M. R: 915-291
Nagamatsu, L.: 3295-68
Nagel, C.: 1395-248, LB1545-356
Nägga, K.: 2215
Naglie, G.: 1410-329, 2550
Nagpal, R.: LB2570-387
Nagy, T. R: LB2570-371
Nah, S.: 3320-146
Naidoo, N.: LB1545-342
Naik, A.: 850-145, 3345-219, 3560
Naim, S.: LB935-440
Naiomi, R.: 3320-134
Naito, T.: LB2570-320
Najafi, B.: 1402-297, 2360-99, 3325-166, LB935-381
Nakagawa, T.: 865-171, 1325-69, 3340-210, 3370-293
Nakajima, M.: LB1545-344
Nakajo, K.: 895-232
Nakamura, J.: 1540
Nakamura, M.: 820-19
Nakayama, R.: 925-319
Nakazato, Y.: 4005
Nambisan, P.: 1401-276
Namihira, Y.: 3280-30
Namkung, E.: 640, 1300-3
Narasimhan, B.: 1270, LB1545-385
Narita, M.: 3340-204
Naruse, T.: 895-225
Naseh, M.: 1390-237
Nash, P.: 1355-150, 1420, 2375-150
Nath, R.: 3370-301
Nathanielsz, P.: 835-94
Nation, D. A: LB3620-411
Natonson, A.: LB1545-430
Naumovski, N.: 705
Navar, E.: LB935-351
Navarette Muñoz, E.: LB935-430
Navarro, E.: 1402-296
Naylor, M.: 2215, 3230
Neal, M. B: 3185
Nearing, K. A: 1335-97, 2385-173
Nedjat-Haiem, F. R: 1000
Nedjat-Haiem, F.: 3275-22
Needham, B.: 925-337
Neelamegam, M.: 3070, 3075
Neese-Todd, S.: 910-274
Neff, L.: 1350-132
Nehlin, J.: LB2570-339
Nehrkorn-Bailey, A. M: 890-221, 925-334
Neiberg, R.: 3325-175
Neiberg, R.: 3325-155
Neiva, C. M: 895-235
Nelson, C. L: 1380-196
Nelson, C.: 3275-20
Nelson, E. L: 2340-47
Nelson, H.: 2390-198
Nelson, H.: 710, 2345-57
Nelson, M.: 870-180
Nelson, M.: 3555
Nelson, M.: LB3620-391
Nelson, N. A: 3560
Nemoto, Y.: 895-226
Neppl, R.: 820-40
Nerella, S.: 2410-264
Nett, B.: LB2570-421
Nettlefold, L.: 855-147
Neubauer, N.: 2325-31
Neugebauer, V.: LB935-431
Neumann, A.: 900-246
Neupert, S. D: 700, 2040
Neupert, S. D: 3350-246
Newell, J.: 920-299
Newman, A. B: 565, 1425, 3275-24, 3440, 3595, LB1545-432, LB2570-396, LB3620-348, LB935-368
Newman, D.: LB935-379
Newman, M.: 635
Newsom, J. T: 1395-248
Newton, N. J: 1140
Neyland, B. R: LB935-403
Ng, B.: 2360-88
Ng, H.: 2400-223
Ng, I. H: 2360-95
Ng, J.: 2250
Ng, R.: 2360-96
Ng, T.: 715
Ng, W.: 910-276, 1405-324, 2390-200
Ng, Y.: 925-316, 1350-134
Ngo, L. H: 825-48, 4130
Ngo, S.: 2315-1
Nguyen, A. W: 2405-232, 3475
Nguyen, C. M: 3350-239
Nguyen, H. M: 3365-272
Nguyen, H.: 1402-297, 2360-99, 3325-156
Nguyen, H. Q: 3175
Nguyen, L. T: LB2570-415
Nguyen, M.: LB1545-334, LB2570-377
Nguyen, Q.: 1345-116
Nguyen, T. T: LB2570-369
Ni, L.: LB3620-336
Ni, P.: 3535
Ni Leime, A.: 560, 865-169, 4125
Nichols, J.: 2430-298
Nichols, K.: 1320-64
Nichols, L.: 3165
Nichols, M. J: LB935-370
Nicholson, H.: 2425-284
Nicklas, B. M: 3325-155, 3590, LB935-403, LB1545-387
Nickols-Richardson, S.: 2360-100
Nicolay, S.: 3300-93
Nicosia, F.: 3485
Niculescu, I.: 2355-61
Nie, J.: 1310-41
Nie, Q.: 1445, LB2570-415
Nielsen, K.: LB2570-435, LB3620-378
Nieman, C. L: 4155
Niemeyer, C.: 3315-122
Nightingale, G.: 855-146
Nijpels, G.: 635
Niles-Yokum, K.: 735, 1265
Nishide, K.: 1305-23
Nishimoto, M.: 895-232
Nishino, A.: 1305-23
Nishita, C. M: 2325-22
Niznik, J. D: 3390
Nkimbeng, M.: 3360-268
Noah, A.: 3375-305
Noblitt, J. A: 1380-193
Nobrega, P.: 3365-281
Noel, O. R: 1390-226
Noguchi-Watanabe, M.: 895-225
Noh, H.: 1260, 1315-56, 2390-201, 2395-211, 3300-98, 3345-228
Noh, J.: 820-32
Nonaka, K.: 895-226
Noppert, G. A: 3095
Nordahl, T.: LB3620-399
Noriega de la Colina, A.: 3280-29
Norman, S.: 2380-168
Norris, K.: 3360-260
Northrip, K.: 505, 3375-303
Northrup-Snyder, K.: 1360-160
Norton, B.: LB2570-445
Norton, E.: LB1545-377
Notthoff, N.: 3615
Novak, J.: LB2570-326
Novik, N.: 1515, 2355-62, 2430-296, 3140
Nowacek, N.: 4080
Nsobundu, C. K: 1360-151
Nuccio, A.: 1380-194, 1405-321
Nuccio, A. G: 3370-287
Nunes, T.: 2325-28
Nwakasi, C. C: 805
Nwankwo, I.: 2565
Nyandege, A.: 850-143, 4135
Nygard, L.: 3565

### O

O Malley, K. A: 880-193, 2325-25, 3300-97, 3320-149
O' Neill, S.: 4055
Oakes, C. E: 2435-307
Öberg, P.: 3200, 3315-124
O'Brien, C.: 2340-33, LB935-367
O'Brien, T.: 2410-254
Obuchi, s.: 855-154, LB2570-411
Ochsner, K.: 1385-219
OConnor, M. L: LB3620-441
O'Connor, D.: 2340-33, LB935-367
O'Conor, R.: 3595
Odd, K.: 865-177
Odden, M.: 3595, 4045
O'Dea, E.: 3285-39
Odejimi, A.: LB3620-345

O'Donnell, D.: 2385-178, 3025
Ofstedal, M.: 1305-30, 1350-122, 3230
Ogawa, E. F: 2255
Ogawa, E. F: LB1545-363
Ogino, R.: 925-319
Ogita, A.: 830-63
Ogletree, A. M: 1315-51
Oh, G.: 2340-35
Oh, G.: LB2570-375
OH, H.: 1395-242
Oh, P. A: 1010, 1305-32
O'Hair, M.: LB1545-419
OHanlon, C. E: 3530
O'Hanlon, A. M: 2340-50
Ohya, Y.: 3280-30
Ojeranti, D.: 3590
Okamoto, S.: LB935-439
Okata, J.: 925-330
Okoye, S. M: LB2570-382
Okubo, S.: LB2570-444
Oladimeji, A.: 890-218
Olde Rikkert, M.: 1402-304
Oleskiewicz, D.: 925-325
Olinger, S.: 2435-310
Oliva, C.: 2325-31
Olivari, B.: LB935-362
Oliveira, M.: 2360-87
Oliveira, R. F: 895-235
Ologhobo, T.: 1365-179
Olsen, B.: 2385-188
Olson, A.: 825-46
Oluwaleimu, O.: 890-220
Olzmann, A.: 1355-142
Olzmann, A.: 2375-149
Omar, A.: 3415
Onder, G.: 3170, 3325-173
O'Neal, A.: LB2570-425
O'Neil, A.: 3275-18
O'Neill, D.: 1490
O'Neill, H.: 3600
Ong, A.: 3340-206
Ong, A.: 4100
Ono, S.: 3270-4
Op het Veld, L.: 3270-5
Opalinski, A.: LB935-379
OPTIC Research Team, C.: 4130
O'Rand, A.: 2205
Orange, J.: 2355-75, 3560
Orav, E. J: LB2570-367
Ornelas, E. X: 925-335
Ornstein, K.: 840-115, 1260, 2280
Orozco, T.: 840-108, 3320-137
Orr, M. E: 820-39, 825-56
Orsak, B.: 835-91
Orsulic-Jeras, S.: 1160, 3300-93
Ortega, F.: 1402-312
Ortega, J.: 2360-91
Ortenberg, E. Kwee: 1125
Ortiz, L.: LB935-363
Orwig, D.: 820-21, 2370-122, 3330-188
Orwig, D.: 4130
Orwoll, E. S: 820-22
Orwoll, E.: 1120
Ory, M. G: 635, 3300-96, 3320-136
Orzeck, P.: 2045
Osawa, Y.: 820-24
Oseau, T.: LB3620-425
Oslin, D.: 1330-81
Ossenfort, K. L: 1385-209
Ostbye, T.: 865-167
Osteen, K.: 3560
O'Sullivan, R.: 2500
Osuna, M.: 4000
Oswald, A.: 3445
Otali, E.: LB1545-431
Otieno, D.: 2130
Otmanowski, J. A: LB1545-338
O'Toole, E.: 875-187, 3510
Otsubo, M.: LB2570-345
Otsuki, T.: 1305-23
Ottenbacher, K.: 850-135
Ottenbacher, K.: 850-139, 2385-171, 4020, 4135
Otters, R.: LB3620-434
Ottmar, H.: 1355-142, 2375-149
Otu, H.: 825-48, 4130
Ou, A.: 1515
Ou, T.: 3335-195
Ouchida, K.: LB3620-396
Oude Mulders, J.: 865-172, 3000
Oude Veldhuis, S.: 3230
Oura, M.: 820-27
Ouyang, M.: 3280-32
Overcash, J. A: 3275-26
Overholser, J. C: 1330-84
Owczarzak, J.: 3275-14
Owen, D. C: 2395-207
Ownby, R.: LB3620-379
Owusu, C.: 1395-243
Oyetunji-Alemede, C. O: 890-220
Ozer, E.: 1402-312

### P

Pace, G. T: 890-222, 1075, 1345-111
Pachana, N. A: 850-130
Packard, K.: LB935-420
Paes, A. T: LB1545-437
Pagnini, F.: 2340-38
Pahlen, S.: 1425
Pahor, M.: LB3620-403
Pais, I.: 1515
Pajewski, N. M: 3590, 4045
Palazzo, L.: LB2570-368
Palekar, N.: 825-52
Palimaru, A.: 3305-104
pallaki, m.: 1320-63
Palmarini, N.: 1515, 3520
Palmer, J.: 3555
Palmer, L. A: 2385-190
Palmer, R. F: 1360-158, 3275-16
Palmer, T.: 2310
Palmisano, K.: LB935-443
Palta, P.: 3075
Pan, A.: 1300-12, 3360-271
Pan, H.: 3250
Pan, J.: 3360-265
Pan, X.: LB2570-361
Pan, Y.: 895-233
Pan, Z.: LB2570-393
Panayi, A. C: 820-40
Panesar, M.: 855-150
Pangle, A.: LB3620-369
Papenkov, M.: 2155
Papp, K. K: 875-187
Parajuli, J.: 3010
Parayil, M.: 930-341
Parekh, R.: 1500, 2405-239
Park, C.: 2385-175
Park, D.: 880-192
Park, E.: LB3620-436
Park, E.: 2340-51
Park, G.: LB2570-398
Park, G.: 3325-172, 3330-189
Park, H.: 640
Park, H. K: LB1545-425
Park, H.: 890-215
Park, J.: 3460
Park, J.: 4160
Park, J.: 3295-85, 3325-170
Park, N.: 1325-70, 2405-235
Park, S.: LB2570-337
Park, S.: 515, 845-118, 2030, 2430-294, LB3620-335
Park, S.: 1310-35, 3290-60
Park, S.: 2405-245
Park, Y.: 910-258
Parker, B. M: 925-328
Parker, D. C: 820-41, 4130
Parker, D.: 505
Parker, L.: 1450
Parker, S. D: 3355-247
Parks, C.: LB2570-322
Parks, J.: 835-87
Parmelee, P. A: 915-281, 1095, 1385-217, 1400-264, 2375-142, 2415-265, 3290-55, 3365-279
Parniak, S.: LB3620-368
Parra-Rodriguez, L.: 3590
Parsons, P.: 2425-280, 3300-94
Paryani, F.: LB935-345
Pasinetti, G. M: 825-57
Pasquini, G.: 3290-52
Pasta, P. M: 1380-200
Pastells-Peiró, R.: 3345-216
Pastva, A. M: 2345-56
Patel, K.: 1305-33
Patel, K.: 570
Patel, M.: 3535
Patel, P.: 830-65
Patel, P.: 1360-164
Patorno, E.: 3050, 3325-169
Patrick, J.: 1345-112
Patskanick, T.: 3180
Pattanaik, S.: LB1545-391
Patterson, A. D: LB3620-347
Patterson, M. E: 3390
Patterson, W.: LB3620-327

Patton, S. K: 3330-181, LB1545-343
Paudel, A.: 2390-194
Paul, S.: 710
Paúl, C.: 880-201
Paúl, C.: 1402-307
Paúl, C.: 2325-28
Paúl, C.: 3325-158
Paules, S.: 2325-28
Paulson, D.: 1320-62, 1355-140, 2550, LB1545-353
Paulson, H.: 500
Pauly, T.: 2275, LB3620-383
Paun, O.: LB2570-419
Pavão, R.: 1390-222
Pavon, J. M: 1402-308
Pawar, A.: 3050, 3325-169
Payer, C.: 2390-206
Payne, M.: 2380-167
Peacock, E.: 1315-52
Pearman, A.: 3350-246, LB2570-373
Pearsall, V.: 835-84
Pearson-Littlethunder, J.: 1385-208
Pebole, M.: 1330-83
Pedersen, N.: 1215
Peek, G.: 3320-145
Pei, Y.: 1105, 2340-43, 4115
Peitz, L.: 2365-110
Pel-Littel, R. E: 1365-176
Penate, C.: 2385-188
Pence, B.: 835-85
Pendergrast, C.: 1405-320
Peng, C.: 3270-10
Peral Gómez, P.: LB935-430
Perales Puchalt, J.: LB935-437
Perego, R.: 820-31
Peremen, Z.: 1402-293
Perez, A. G: 605
Perez, E.: 1390-239, 2420-272
Perez, K.: 2375-140
Perez, V. I: 1520
Pérez-Zepeda, M.: 565, 3590
Perion, J.: 1355-146, 2355-73
Perissinotto, C.: LB3620-425
Perkhounkova, Y.: LB2570-364
Perkins, M. M: 885-211, 2485, 3270-9, LB2570-351, LB3620-321
Perkins, R.: 2325-24, LB935-358
Perkinson, M. A: 2220
Perkovic, S.: 2400-222
Perls, T. T: 835-92, 1120, 2345-55, LB2570-338
Perone, A.: 3430
Perrig-Chiello, P.: 3200
Perrin, N.: LB2570-382
Perry, M.: 3175
Perry, T.: 4060
Perskinson, M.: LB3620-374
Perweiler, E.: 885-204, 4075
Perzynski, A. T: 835-86
Pessoa-Filho, D.: 895-235
Pestine-Stevens, A.: LB2570-388
Peters, O.: LB3620-318
Petersen, J.: LB2570-339
Peterson, C. M: 1385-210, 2375-140
Peterson, J. R: 1325-66, LB935-360
Peterson, L. J: 1405-325, 3345-215
Peterson, M.: 1395-240
Peterson, M. J: 3170
Peterson, N. L: 2280
Peterson, R. L: 3120
Peterson, T. L: 1350-133
Petkus, A. J: LB2570-441
Petropoulos, A.: 2045
Petroski, G.: 4135, LB2570-334
Petrovsky, D. V: 670, 1160, 1435, 2215, 2420-276, 2530
Petti, E.: 3220, 3365-282
Pettis, J.: 2395-212, LB1545-335
Petty, R.: 1315-52
Pfund, G. N: 585
Pham, A.: 850-126
Pham, T.: 885-210
Pham, T. N: 850-136
Phan, R. V: LB935-346
Phelan, A. A: 2385-178, 3025
Philips, K. L: 1330-84
Phillips, C.: 1150, 3290-61
Phillips, J. R: 2065, 2425-284, 2450
Phillips, K.: 3365-272
Phillips, L. R: 3120
Phillips, L. J: LB2570-334
Phillipson, C.: 510, 3085
Phinney, A.: LB3620-344
Phipps, K.: 2385-185
Phoenix, A.: LB935-418
Phongtankuel, V.: 3310-118
Piao, Y.: 835-99
Picchiello, M.: 3310-116
Piccinin, A.: 3340-206, LB2570-347
Pichiello, M.: 3270-6
Pichora-Fuller, M.: 850-142
Pickering, A. M: 825-62
Pickering, C. E: 2375-144, 2465, 3375-313
Pidgeon, D.: 3545
Piedra, L. M: 3360-270
Pieper, C.: 3365-275, 4130, 4145
Pierce, G.: LB935-350
Pierce, L.: 1355-147
Pierce, L.: 1355-146
Pierpaoli Parker, C.: 2420-277
Pietrobon, R.: 2240
Pietzak, L.: 2385-185
Pike, J.: 1160
Pillai, V.: 2250
Pillemer, K.: 1250, 2085, 3235, 3315-131, 3365-282
Pilling, L. C: 1180, LB2570-393, LB3620-333
Pilonieta, G.: LB2570-391
Pinheiro, A.: LB2570-325
Pinto, J.: 2425-291
Pinto Pereira, S.: 685
Pirkle, C. M: LB935-426, LB1545-392
Pirkle, C. M: LB2570-421
Pisegna, J.: 1315-55
Pitt, J.: 830-81
Pizzi, L. T: 1005
Pizzini, J.: 835-93
Plassman, B. L: LB2570-322
Platt, J.: 880-197
Platt, J.: 2170
Platts-Mills, T.: 795
Pleasants, D.: 850-132
Pless Kaiser, A.: 3300-97
Plocher, T.: 1385-214
Ploeg, J.: 1395-245
Plotkin, E.: 855-146
Plotzke, M.: 520
Plude, D.: 1125
Plys, E.: 3270-13
Podolskiy, D.: 625
Poey, J.: 2515
Poggio, J.: LB2570-425
Poggiogalle, E.: 1402-298, 3325-159
Pohl, J. S: 2295, 4150
pohnert, A.: 895-240
Polekhina, G.: 870-180
Polenick, C. A: 725, 3425
Polivka, L.: 1220, 3085
Polk, B. K: 1385-207
Pollak, K. I: LB2570-403
Polson, C.: 2425-289
Polter, E.: 2380-155
Pomilio, C.: 825-54
Poon, L.: 925-322, LB3620-389
Pope, C. N: 1360-156
Pope, N.: 1305-14
Popejoy, L. L: 4135
Popescu, M.: 820-37
Popham, L. E: 3415
Popoola, B. I: 3300-100
Popovich, A.: 2425-280
Portacolone, E.: 2010, 2215, LB2570-336
Porter, H.: 505
Porter Starr, K. N: 2070, 2380-167
Porterfield, S. L: 2030
Portz, J. D: 1080, 1401-291
Post, K.: 1355-148
Potapragaa, N. R: 2385-176
Pothen, J.: 3340-207
Potter, K.: 1090
Poulin, L.: 2105
Powell, D.: 3075
Powell, M. L: LB2570-371
Power Cyr, J.: 840-112
Powers, B.: 1335-96, 3375-304

Powers, D. A: 3335-196
Powers, J. S: 2565
Powers, S.: LB3620-352
Prabhu, A.: 1402-309
Pradeep Kumar, D.: 820-26
Pradiee, J.: 835-83
Prakobchai, S.: 3350-235
Prasad, A.: 3100
Prater, L.: LB2570-345
Prescott, C. A: 655
Presnell, A.: 2310
Pretter, S.: 2430-300
Pretzer-Aboff, I. A: LB2570-356
Price, N.: 1120
Prieto, A.: 3370-290
Prieto, L. R: LB935-432
Prina, M.: 4040
Prins, M.: 840-110, LB1545-414
Prior, S. J: 3020
Pristavec, T.: 1160, 3310-115
Prizment, A.: 710, 2345-57
Proff, K.: 2325-23
PROMISE Study Group: 3355-251
Promislow, D.: 1245
Propson, N. E: LB935-364
Proulx, C. M: LB3620-332
Pruchno, R.: 1315-51, 2520
Pruett, M.: 3535
Pruitt, A.: 2405-241
Prusak, A.: 2415-269
Prvu-Bettger, J.: 630
Pu, C. T: 870-181
Pud, D.: 2315-7
Puga, F.: 2375-144, 3375-313
Pugh, M.: 3295-76
Pulok, M.: 3590
Pupo, D.: 3295-71
Puro, N.: 2185
Pusopa, C.: 3350-235
Putnam, M.: 1235
Puurveen, G.: LB3620-344
Pynoos, J.: 3270-3

### Q

Qato, D.: 3355-253
Qian, X.: 2125
Qiao, Y.: LB2570-343
Qin, W.: 4150
Qin, X.: 2155
Qiu, P.: LB3620-403
Qiu, X.: 4150, LB935-402
Quach, E.: 740, 3535
Quach, L.: 1360-163, 2255
Qualls, S. H: 1100, 2375-143, 3320-149
Quan, N. S: 885-211
Quan, N.: 3600
Quest, T.: 3270-9
Quinn, C. C: 530, 1402-311
Quinn, K.: 1401-283, 3115
Quiñones, A. R: 1395-248, 1450, 3325-163, LB1545-356
Quintana, C.: LB935-384
Qvist, J. Y: LB2570-318

### R

Rabinovitch, P.: 835-101
Radhakrishnan, K.: 1395-247, LB1545-419
Radusky, P.: LB935-417
Raftery, J. P: 2380-161
Rager, J. R: LB1545-432
Raghavachari, N.: 3215
Rahman, A. N: 1340-98
Rahman, A.: 3330-183
Rahman, A.: 3330-182
Rahman, A. N: LB1545-443
Rahman, M.: 3140
Rahman-Filipiak, A. M: 500
Raichur, V.: LB2570-435, LB3620-378
Rainoldi, A.: 1402-298, 3325-159
Rainville, G.: 1401-279, 2340-39, 2420-271, 3410, LB3620-366
Raitanen, J.: LB1545-339
Raj, M.: 880-197
Raji, M.: 850-135, 3355-250, LB1545-427
Rajpara, A.: 915-289
Raldiris, T.: 3370-288
Ram, N.: 2005
Ramachandran, A.: LB3620-329
Ramadhani, W. A: LB1545-373
Ramel, A.: 920-309, 2370-126
Ramesh, S.: LB935-366
Ramirez, A.: LB3620-318
Ramírez-Aldana, R.: 3590, 4155
Ramos, A. M: 1315-45
Ramsay, J.: 3365-280
Ranahan, M.: 3330-187
Randall, G. K: 1475
Randolph, A.: LB3620-345
Ranger, V.: 3595
Ranjit, N.: 2360-86, LB935-441
Ranka, S.: 2410-264
Rantz, M. J: 4005
Ranville, M.: LB2570-424
Rapoport, M.: 1410-329, 2550
Rapp, S. R: 850-132
Rasgon, N.: LB935-442
Rashidi, P.: 2410-264
Rasmussen, B.: LB1545-354
Rasmussen, B.: LB3620-345
Rasmussen, L.: LB1545-347
Rasmussen, L.: LB2570-339
Rast, P.: LB2570-355
Rasticova, M.: 4125
Ratcliffe, S. J: 2360-103
Rath, L.: 1355-150
Rath, L.: 2375-150, 3010
Ratner, E.: 3305-106
Ravdin, L.: 3220
Raveis, V. H: 2430-300
Ravi, K. E: 1305-28
Ravyts, S.: 1390-239
Ravyts, S.: 2420-272
Ravyts, S.: 2425-282
Rawlings, A.: 2495, 3595
Rawson, K.: LB3620-399
Ray, M. A: 3290-51
Raymo, J.: 1305-30, 1350-122, 3230, 4065
Rayner, J.: 505
Raz, N.: LB935-366, LB3620-326
Razaq, W.: LB3620-358
Reales, j.: 3370-290
Rebok, G. W: 590, 1125, 2265, 2410-259, 2425-291, 3350-245, 4025
Reckrey, J. M: 840-115, 1155
Recksiedler, C.: 3030, 3200
Rector, J.: 2005
Rector, K.: LB2570-395
Reed, C.: 815-16, 2425-290
Reed, M.: 850-142
Reed, N.: 925-318
Reed, N.: LB935-362
Reed, N.: 665, 2380-166, 2495, 4155
Reed, P. S: 2385-191
Reel, C.: LB1545-406
Reese-Melancon, C.: 1401-285
Reeves, M.: 3415, 4020
Refroe, C.: LB935-425
Regier, N. G: 1005
Reid, C.: 1315-57, 3220, 3310-118, 3365-282, LB1545-341
Reid, C.: 2315-15, 2395-219, 4130
Reilly, P.: LB1545-382
Reilly, P.: LB2570-365
Reilly-Sanchez, L. M: LB1545-408
Reinhard, S.: 3450
Reinhardt, J. P: 2015, LB3620-393
Reinhardt, R.: 1315-45, 1380-194
Reitelseder, S.: 820-30
Reiter, D.: 3295-71
Reive, C.: 3295-75
Rejeski, J.: 3325-175
Religa, D.: 2215
Rely, K.: 4155
Remillard, E. T: 3500
Ren, Y.: 2345-56
Renfro, M.: 3270-3
Renn, B. N: LB935-396
Renner, S. W: 1240, LB1545-428
Reno, J.: 1305-16
Renteria, M.: LB1545-410
Rentsch, J. H: LB3620-362
Renzi-Hammond, L. M: LB3620-329
Resciniti, N. V: 3115, 3325-164

Resnick, B.: 705, 920-301, 1255, 2315-3, 2390-194, 2525, 3270-11
Resnick, S. M: 2370-129
Resnick, S.: 3075, LB2570-441
Restorick Roberts, A.: 1480, 3025
Restrepo, M. I: LB1545-408
Restrepo, V.: LB1545-408
Retalic, T.: 4080
Rey, S.: 1150
Reyes-Ortiz, C.: 2365-115
Reynolds, A. S: LB1545-383
Reynolds, C. A: 925-338, 1150, 1425
Reynolds, C.: LB2570-324
Reynolds, C. L: LB2570-357
Reynolds, C.: LB2570-383
Reynolds, K.: LB2570-373
Reynolds, L.: 680
Rhee, M.: LB3620-433
Rhee, M.: 1325-70
Rhee, M.: 2405-235
Rhee, T.: 3010
Rhodes, J.: 1170, 2280
Rhoten, N.: 2360-101
Ribeiro, O.: LB2570-341, 645, 880-201, 3180
Ricamato, A.: 900-243
Richards, C.: 1385-204
Richards, l.: 660
Richardson, S.: 865-168
Richbart, M.: 3330-187
Richey, S.: LB2570-420
Richman, K.: 2380-154
Richmond-Cullen, c.: 705
Richmond-Rakerd, L.: LB1545-347
Rickard, T.: 3435
Ricke, W. A: LB1545-404
Rickenbach, E.: 4035
Ridings, J.: 3360-269
Rieder, N.: LB3620-351
Rieker, J.: 3370-290
Riffin, C.: 1110, 3180, 3365-282
Riley, A.: 4070
Rincon, J.: 835-83
Ring, L.: 1070, 1210
Rippon, I.: 650
Risal, P. G: LB3620-327
Risbud, R.: 3535
Ritchie, C.: 1305-33
Ritenour, D.: 520
Rivera, E.: 3230
Rivera, J.: 3330-189
Rivera Almaraz, A.: 3325-154
Rivera-Burciaga, A.: 1365-171
Rivera-Hernandez, M.: 2545, 3330-178, 3585
Rivers, E.: 3155
Rizzotti, R.: 3330-189
Rizzuto, D.: 850-137, 3170, 3325-173
Roach, K.: 775
Roat-Shumway, S.: LB935-442
Robayo, C.: 2355-66
Robbins, J. C: 2405-236
Robbins, L.: 1365-179
Robbins, R.: 2325-29
Robbins, R.: 2360-86, LB935-441
Roberge, B.: 755
Robert, M. N: 885-211
Robert, S.: 910-260
Roberto, K. A: 1025
Roberto, K. A: 3345-223
Roberto, K. A: 3515
Roberto, K. A: LB1545-405
Roberts, A.: 1325-74
Roberts, E.: LB935-392
Roberts, J.: 3070
Roberts, L. C: 515
Roberts, L.: 1365-179
Roberts, L.: LB935-371
Roberts, M.: 1402-293
Roberts, T.: 850-131
Roberts, T.: 1365-179
Robertson, E.: LB3620-424
Robinson, E. L: 2485, LB2570-398
Robinson, J.: LB935-425
Robinson, K. N: LB3620-323
Robinson, L.: LB2570-319
Robinson, S. A: 2255
Robinson, T.: 2115
Robison, J.: 1065, 1305-25, 2430-297
Robnett, R. H: LB1545-421, LB3620-350
Rockwood, K.: 565, 820-23
Rockwood, K.: 3325-172
Rockwood, K.: 3325-167, 3590, 4045, LB2570-354
Rodney, O.: LB935-347
Rodriguez, A.: 2345-53
Rodriguez, D.: 815-16, 2425-288
Rodriguez, K.: 830-69, 3385
Rodriguez, K.: LB935-447
Rodriguez, M.: 3220
Rodriguez, M.: 2355-66
Rodriguez Díaz, M.: 3290-67
Rodriguez-Suarez, M.: LB1545-361
Roes, M.: 785
Rogan, J.: 4080
Roger, K.: LB2570-424
Rogers, N.: 685, 1200
Rogers, R.: 830-65
Rogers, S.: LB935-443
Rogers, W.: 550, 620, 1130, 3500, LB1545-373, LB2570-406
Roggio, A.: 1402-311
Rogina, B.: 830-65
Rogowski, J.: 2360-103
Roh, H.: LB1545-398
Roh, S.: 1300-4, 2405-245
Rollins, J. A: 830-66
Roman, C.: 925-317
Romano, J.: 1402-301
Rome, V.: 2395-214
Romero, K.: 2345-56
Rondeau, K.: 3435
Rook, K.: 925-325, 1400-255, 4015
Root, E. Z: 1380-199
Roque, N. A: 4085
Rosa, B.: 895-235
Rosales, K. B: 4030
Rosano, C.: 805, 820-38, 2360-94, 3075
Rosati, R. J: 850-143
Rose, J.: 1395-243
Rose, K.: 3100
Rose, M.: 1385-207, LB2570-383, LB3620-354
rose, t.: 895-229
Rose III, D. W: 2425-290
Rosemond, C.: 520
Rosen, B.: 3420
Rosen, S. L: 3420, 4030
Rosen, T.: 795, 2225, 2370-118
Rosenberg, A.: 3360-265
Rosenberg, D. M: 875-187, 3510
Rosenberg, D. E: 570
Rosenberg, D.: 920-304, 2410-256
Rosenthal, M.: 910-274
Rosenthal, R.: 2115
Rosenwohl-Mack, A.: LB2570-336, LB3620-419
Rosich, R. M: LB1545-348
Roso-Llorach, A.: 3170
Ross, C.: 525
Ross, I. S: LB2570-365
Ross, J.: 925-328, 1335-96, LB1545-366, LB2570-365
Ross, L.: 1410-328
Ross, L.: 1150, 1410-327, 3290-63
Ross, S.: 900-247
Rosselli, M.: 2355-66
Rosser, B.: 2380-155
Rossi, D.: 2385-185
Rossman, M.: 835-89
Rossner, R. J: LB3620-388
Rosso, A.: 820-38, 2170, 2360-94
Rostant, O.: LB935-419
Rostgaard, T.: 1445
Rostowsky, K.: 825-61
Rote, S.: 750, 3360-267
Roth, D. L: 1170, 1250, 2270, 2280, 3420, LB2570-353
Rothberg, Z.: 2405-241
Rothermund, K.: 2040
Round, T.: 3550
Rourk, R.: 2315-8
Rouse, H. J: 2355-72
Roush, R.: 1490
Rousseau, J.: 850-134
Rowan, N. L: 3430
Rowe, B. H: 4130
Rowe, M.: 3495
Rowland, S. A: LB1545-338
Rowles, G. D: 1185

Rowley, S.: 810-2
Roy, E.: 825-47
Roy, E. R: LB935-364
Royall, D. R: 1360-158, 3275-16
Rozario, N. L: LB2570-327
Rozario, P. A: 3065
Rubtsova, A. A: 3525
Rudolph, J.: 705
Rudolph, J.: LB2570-329
Ruffini, G.: 4020
Ruggeri, M.: 1515
Ruggiano, N.: 880-193, 2325-25, LB1545-376, LB2570-384
Ruiz, D.: 1330-94, 1402-299
Ruiz, J. G: LB1545-361, LB3620-338
Ruiz, M.: 895-230
Ruiz, S.: 3500
Rupper, R.: LB2570-416
Rurka, M.: 3315-131, LB935-416
Rush, J.: LB2570-347
Russell, C.: 2410-254
Russell, D.: 1015
Russell, D.: 4010
Russell, J.: 825-59, 2230
Russell, M.: 915-290
Russell, S.: 2395-213
Rustin, P.: 2045
Rutenberg, A.: 825-55
Rutenberg, A.: 3325-167
Rutherford, B.: 2495
Rutledge, M.: 1315-47
Ryan, A.: 820-21, LB1545-424
Ryan, G.: LB3620-408
Rybarczyk, B.: 2425-282
Ryff, C.: 1425
Ryskina, K.: 3390
Ryu, B.: LB3620-335
Ryu, H.: 1365-173
Ryu, J.: 2410-246

### S

Sá, C.: 1402-307
Saad-Lessler, J.: 2380-154
Sabatini, S.: 2040
Sacco, P.: 3335-192, LB3620-355
Saccon, T.: 835-83
Sachdev, P.: 655
Saczynski, J. S: 1400-260
Sadak, T.: 3110
Sadamitsu, K.: 2385-188
Sadarangani, T.: 1205
Sadeq, N. A: 3580
Saenz, J.: 4000
Saenz Monsivais, V.: LB1545-427
Safi, N.: 830-64
Sagiraju, H.: 3295-76
Sagna, A.: 1330-79
Sagong, H.: 1402-305
Sahoo, S.: 1325-71, 1400-259, 1401-290, 3300-99, 3335-192
Sakai, A.: 830-63
Sakai, E.: 925-319
Sakai, E.: 1401-286
Sakaniwa, R.: 1045
Sakata, K.: LB2570-440
Sakka, M.: 895-225
Sakurai, R.: 895-226, LB2570-411
Sala, L.: 820-37
Salari, S. L: 2365-116
Salas, R.: 2420-277
Salazar, M.: 3355-255
Saldana, S.: 1402-296
Salem, G.: 920-303
Salerno, E.: 2050
Salgado, G.: LB935-383
Saliba, D.: 520, 3430, 4135
Salimi, S.: 820-21, 3405
Salinas, I.: LB2570-419
Salinas Rodriguez, A.: 3325-154
Salisbury, D. L: 3175
Salmon, A. B: 820-35, 830-70, 835-88
Salmon, C.: 885-210
Salomons, T.: 905-255
Salthouse, T.: 655, 3290-49
Šaltytė -Benth, J.: 1330-86
Saltzman, B. S: 2370-123
Samadani, A.: 1402-306
Samanez-Larkin, G. R: 1485
Sammy, M. J: LB2570-371
Sampathkumar, N.: LB935-351
Samper-Ternent, R.: 3220, LB2570-332
Sampson, L.: 1345-116
Samsi, K.: 850-138
Samuel, L.: 515
Samuel, M.: LB3620-397
Samuel, M.: LB3620-398
Samus, Q. M: 2490, 3420, 4025
Sanchez Arenas, R.: 1310-40
Sanchez-Niubo, A.: 4040
Sánchez Pérez, A.: LB935-430
Sanchez-Reilly, S.: LB1545-366, LB2570-365
Sanders, A.: 3570
Sanders, J. L: 1425, 3440
Sandlin, A. M: 815-16, 2340-44
Sands, B.: 830-78
Sands, L. P: 1095, 1190, 1315-51
Sanford, J.: 3270-3
Sanghi, S.: 3320-136
Santana, C.: 895-235
Santanasto, A. J: 2370-136, 3170, 3275-24, LB935-368
Santangelo, K. S: 835-88
Santiago, E. D: 825-52
Santiago, H. J: 2415-268
Santini, T.: LB2570-324
Santos, E. J: 1265
Santos, P. M: 2325-28
Santos, T.: 3535
Santos, Z.: 895-235
Sappington VandeWeerd, E.: 1402-293
Sapra, M.: 1355-145
Sarhadi, K.: 1402-301
Sartor, O.: LB1545-377
Sarveswaran, S.: LB2570-354
Sathavarodom, N.: 835-91
Satiroglu, R.: 920-310
Satizabal Barrera, C.: LB3620-372
Sato, T.: 895-232
Saunders, K. C: 2395-212
Saunders, M.: 1435
Saunders, P.: 610
Sautter, J. M: 2355-70
Savage, A.: 880-192
Savla, J.: 1025, 1095, 1315-51, 1355-145, 3480, 4035, LB1545-405
Savova, Z. A: 3285-43
Savundranayagam, M. Y: 2355-75, 3200, 3560
Sawchuk, K.: 580
Sawyer, B.: 3360-258
Sawyer, J.: 815-15, 2340-44, 2425-288
Saxon, A.: LB1545-412
Sayer, J.: LB935-367
Sayer, L. C: 4065
Scala-Foley, M.: 2385-183
Scales, A.: 1401-286, 3530
Scales, K.: 2305
Scanlon, D.: LB1545-397
Scerpella, D.: 1005
Schaeffer, J.: 1015
Schaepe, K.: 3180
Schafer, M.: 830-67, 835-84
Schafer, M. H: 885-209, 925-329, 1345-108, 3030, 3480
Schaffer, R.: LB3620-352
Schaie, W.: 3290-47
Scharre, D.: 845-117
Scheidt, R.: 2145
Scheirer, J. L: 830-69, LB935-447
Schell, B. R: 820-29
Schell, K.: LB935-391
Schepens Niemiec, S. L: 890-213
Schepisi, I.: 3290-62
Scher, C.: LB935-444
Scherer, A. M: 540
Scheurich, J.: LB1545-353
Schiaffino, M. K: 2345-58
Schiffman, R.: 2410-258
Schiller, M.: 1365-166
Schilling, O. K: 3290-57, 3600
Schiltz, N.: 1395-243
Schinka, J.: 2355-72
Schino, R.: 1515
Schiwal, A. T: LB1545-394
Schlag, K. E: LB3620-385
Schlatter, J.: LB1545-402
Schlundt, D.: 2565
Schmall, V.: 3560
Schmidt, B. R: 920-314
Schmitt, E.: LB1545-402, LB2570-346
Schneeweiss, S.: 3325-169
Schneider, A.: 835-83
Schneider, C. E: 870-184, LB935-401

Schneider, E. C: 810-3, LB935-392
Schneider, J.: 3375-310
Schneider, J. A: 3480
Schoemann, A.: 2410-249
Schoenmakers, E.: 1285, 4150
Scholl, M. M: LB935-378
Schols, J.: 3270-5
Scholte op Reimer, W.: 1365-176
Schoonhoven, L.: 2380-158
Schork, N. J: 1120
Schrack, J. A: 820-24, 2360-92, 2370-129, 2485, 3040, 4165, LB935-446
Schroeder, E.: 1320-63
Schuler, E.: 1350-131
Schulman, D. J: 1402-306
Schultz, M. B: LB2570-323
Schulz, R.: 1055, LB2570-324
Schumacher, J. G: 2315-11
Schumann-Bard, P.: 825-49
Schupf, N.: 2370-125, 3170
Schuurmans, M. J: 2380-158
Schwartz, A.: 2505, 3410
Schwartz, L.: 2305, 2470, 3060, 3435
Schwartz, M. L: 740, 3140
Schwei, R.: LB935-422
Schwertner, E.: 2215
Scicolone, M.: 2375-142, 3290-55
Sciegaj, M.: 1235
Scogin, F.: 2310
Scott, C.: 1355-141
Scott, R.: 1385-203
Scott, R.: LB3620-324
Scott, R.: 2550
Scott, S. B: 825-52, 3290-52, 3370-295, 3575, 4085
Scott, T. L: 850-130
Scrivano, R.: 3290-44
Seals, D.: 835-89
Seaman, A. T: 2055, 3165, 3485
Seaman, K. L: 585
Sears, J.: 2355-69
Sebastiani, P.: 835-92, 2345-55, 3215
Seblova, D.: LB1545-333
Seckin, G.: 1401-273
Secnik, J.: 2215
Seetharaman, K.: 1010
Sefcik, J. S: 605
Sefcik, J.: 915-289
Sefcik, J. S: 2215
Sefcik, J.: LB3620-340
Segal, D. L: 840-105, 915-283, 1390-226
Segal-Gidan, F.: 1100
Segel-Karpas, D.: 700
Segev, D.: 2380-168, 4020
Seguin, C.: 2550
Sehgal, S.: 2120
Seibel, K.: 2405-236
Seino, S.: 3340-204
Sekhar, R. V: 1310-37, 2190, 2485
Sell, C.: 2440
Sendroiu, I.: 515
Sengupta, M.: 2390-203
Senteio, C. R: 3200
Sentell, T. L: LB2570-421
Sepehri, K.: 3325-172
Seplaki, C. L: 1330-92
Serra, M. C: 3020
Serrano, I.: 905-253
Seshadri, S.: 3215, LB3620-372
Sesso, J.: 820-34
Severance, J. J: 900-247, 1530, LB2570-414
Shaaban, C.: 3075
Shacham, E.: 1380-190
Shade, M.: LB2570-395
Shadmi, E.: 3420, LB1545-375
Shah, A.: LB1545-361
Shah, A.: 2375-140
Shah, A. A: 2310
Shah, A.: 2430-300
Shah, K.: 2495
Shah, R. C: 710, 1300-6, 3260, 3480, LB3620-375
Shah, S.: 1360-161
Shahid, F.: 2155
Shake, M.: 1402-315
Shakya, S.: 690
Shalev, I.: 2370-117
Shalini, S.: LB2570-398
Shang, j.: LB1545-380
Shank, S.: 825-53
Shapkin, K.: 840-112
Shardell, M.: 820-24, 3040
Sharifian, N.: 745
Sharma, J.: 3330-190
Sharma, K.: LB1545-374
Sharma, N.: 1401-282, 3535
Sharpe, C.: 3375-312
Sharrett, A.: 830-75, 2495
Shaughnessy, M.: 3530
Shavit, Y. Z: 865-165, 925-323
Shaw, A. R: LB935-437
Shaw, C.: LB2570-364
Shea, K. D: 3120
Shea, K.: LB2570-426
Shea, M.: 715, 3280-33
Shea, S.: 3280-34
Sheehan, O. C: 1170, 2280
Sheets, D. J: 2060, 3470, 3610
Sheldon, M.: 1305-28, 4055
Shelley, M.: 2390-202, 4145
Shellito, N.: 3570, LB935-353
Shen, D.: LB1545-412
Shen, H.: 880-196
Shen, S.: 2360-100
Sheng, W.: 1401-285
Shepherd, G.: 1320-62
Shepherd-Banigan, M.: 880-194
Shepley, M.: 1010
Sherlala, R. A: 1310-42
Sherman, A. M: 1140
Sherman, L. D: 2405-234
Sherrill, C.: 835-87
Shi, J.: 1355-141
Shi, L.: 3220
Shi, X.: 830-73, LB3620-360
Shi, Z.: 4020
Shia, B.: 3330-185
Shih, R. A: 4050
Shim, H.: 1060, 1401-289
Shim, J. K: 1520
Shimabukuro, M.: 830-72
Shin, J.: 3295-77, LB1545-401
Shin, O.: 2430-294
Shin, O.: LB3620-335
Shin, S.: LB935-374
Shin, S.: 1310-35, 3290-60
Shinkai, S.: 3340-204
Shippee, T. P: 910-258, 1405-324, 2155, 2390-200, 3345-217, 3585
Shirai, K.: 1045
Shireman, L.: LB1545-412
Shirmohammadi, F.: 830-64
Shiroma, K.: 2165
Shishegar, N.: 3295-84
Shockey, D.: LB2570-379
Shoemaker, V.: 850-142
Shofner, S. J: LB935-436
Shojaei, A.: 1320-64
Shon, E.: 3350-236, 3360-262
Shook, N.: 3370-298, LB2570-360
Short, M.: LB3620-372
Shovali, T. E: 3320-135
Shrestha, M. K: LB935-402
Shrestha, S.: LB3620-439
Shrestha, S.: LB2570-390
Shrira, A.: 700
Shropshire, K. L: 3455
Shryock, K.: 3310-117
Shuey, K.: 2205
Shulyaev, K.: 2315-7, LB1545-375
Shumaker, E. M: 535
Shumaker, S. A: 850-132
Shuman, S. K: 1435
Shusterman, G.: 2385-193
Siden, R.: 895-230
Sidrak, H.: 925-338
Sieber, S.: 3030
Siebert, J.: 2040
Siedlecki, K. L: 655, 3290-49
Siegel, E.: 2295
Siegel, M.: 900-249
Sieng, M.: LB2570-316
Sierra, F.: 2290
Sikoki, B.: 915-287
Sillito, C.: 2365-116
Sillner, A. Y: LB2570-329
Sills, J.: 2390-196
Silva, D.: 505
Silva-Smith, A. L: 3325-162
Silver, J.: 1315-52
Silver, M.: 1150, 2030, LB3620-391
Silverman, M. A: 2495

Silverstein, M.: 2160, 3335-194
Sim, H.: LB1545-401
Sima, C.: 2410-258
Simmons, E.: 2315-4
Simning, A.: 1330-92
Simning, A.: LB1545-380
Simões Neto, J.: 850-141
Simon, A.: LB2570-342
Simon, C.: 3365-275
Simon, K.: 3555
Simon, M.: 3505, 4105
Simons, K.: 910-275, 1480, 3345-218
Simonsick, E. M: 515, 705, 820-24, 1240, 1455, 2370-129, 3075, 3295-73, LB935-446, LB1545-410
Simpson, D.: LB1545-400
Simpson, E.: 915-288, 1065, 3570
Simpson, F.: 3590
Simpson, T.: LB1545-412
Sims Gould, J.: 855-147, 3330-180
Sims-Gould, J.: 3330-177
Sin, N. L: LB935-411
Sinclair, D.: LB2570-323
Sing, J.: 2400-227, 2425-287, LB3620-440
Singer, D.: 705, 2155
Singh, E.: LB3620-336
Singh, K. K: 2090
Singh, P.: 1402-306
Singh, R.: 3330-187
Singh, T.: 1360-157
Singhatat, W.: LB2570-397
Singleton, M.: 910-270, LB1545-443
Singsank, K.: 855-148
Sinha, I.: 820-40
Sinwoo, H.: 840-111, LB2570-317
Sion, K.: 785
Sioutas, C.: 825-51, 830-64
Sirey, J. 2430-302, LB935-444
Sisofo, B.: LB935-376
Sison, S.: 2380-168
Sivanesathurai, R.: 850-131
Sivers-Teixeira, T.: 2385-188
Sjöberg, L.: 515
Skanderson, M.: 2370-119
Skelton, D. A: 730
Skinner, M.: 560, 2105, LB3620-373
Skipper, A. D: LB2570-417
Sköldunger, A.: 635
Sköldunger, A. B: 3345-220
Skrajner, M.: 500, 2355-63
Skubic, M.: 820-37, 4005, LB2570-398
Skufca, L.: 1401-279
Skultety, K.: LB3620-419
Slattum, P.: 3300-94
Slaug, B.: 3600
Slayday, R. E: LB2570-348
Slindee, L.: 1315-60, 1400-268
Sliwinski, M.: 3290-52, LB3620-347
Sloan, C. J: 2375-148
Sloan, J.: 870-179
Sloan, S.: 3410
Sloane, R.: 2380-167, 4130
Slosser, A.: 870-186
Smaga, S.: 1335-95
Smagula, S.: LB2570-324
Smaling, H.: 4135
Small, B. J: 2355-72, 3290-52
Smalls, B.: 1315-47
Smetcoren, A.: 1305-22, 3035
Smilovich-Fine, E.: 3510, LB1545-355
Smilowitz, J.: 2395-215
Smilowitz, S. T: 3510
Smit, L. C: 675
Smith, A.: 2270, 3470
Smith, A. B: 1350-127
Smith, C.: 825-58
Smith, D. L: LB2570-371
Smith, D.: LB2570-414
Smith, D. M: 1095, 1385-217, 2415-265, 3365-279
Smith, E. E: 3295-87
Smith, G.: 865-162
Smith, G. C: 1350-129
Smith, J.: 720, 925-332, 3340-202, LB935-409
Smith, J. L: 2340-33, LB935-367
Smith, K.: 3275-14
Smith, K.: 1535
Smith, L. N: 815-15, 2340-44, 2425-288
Smith, L.: 2060
Smith, M. L: 635, 770, 1380-190, 2360-103, 3300-96, 3335-193
Smith, R.: 3300-97
Smith, S. K: LB2570-403
Smith, S. A: 1402-300
Smith, S. D: 3430
Smith, T. J: 3300-99
Smith, T.: 895-227
Smith, V.: 880-194
Smith-Ray, R. L: 1360-157
Snodgrass, J.: LB1545-342
Snow, A.: LB3620-351
Snyder-Mackler, N.: 765, 1430
Soares, J.: 895-235
Sober, J.: LB3620-389
Soble, J. R: 3295-76
Sodhi, J. k: LB1545-349
Soh, Y.: 1400-261
Sohn, Y.: 850-131
Solimeo, S.: 3165, 3485
Solinge, V.: 575
Sollid-Gagner, C.: 2395-219
Solow, A.: 915-298, 1330-82
Solway, E.: 540, 705, 2155, 3135
Somerville, C.: 910-265, 2385-179
Son, B.: 820-27
Sonawane, K.: 3330-184
Song, H.: 2395-207
Song, J.: LB3620-412
Song, M.: 710, 840-107, 850-125
Song, M.: LB2570-359
Song, M.: 3555
Song, Q.: 3145
Song, S.: 2165
Song, W.: 4045
Song, X.: 820-28, 3325-172, 3330-189
Song, X.: LB2570-354
Song, Y.: 1340-102
Song, Y.: 2420-273
Song, Z.: 2345-55
Sonnega, A.: 2325-29
Sonnier-Netto, L.: 2435-314
Sorbero, M.: 2395-217
Sörensen, S.: 2130, 2235
Sorkpor, S.: 1402-313
Sorrentino, V. A: 2410-257
Sotelo, V.: 870-184
Soto, M.: 1515
Sou, C.: LB3620-425
Sousa, L.: 3180
Souza, A.: 3115
Spahr, M.: 515
Spalding, R.: 885-203, 3350-243, LB935-405
Spears, E.: 2405-234
Speirs, C.: 2340-40
Spence, M.: 1325-74
Spence, S.: LB3620-420
Spencer, D.: 2225
Spencer, R.: LB2570-372
Spencer-Brown, L.: 3305-107
Spensley, C.: 3135
Sperber, N. R: 1110
Spetz, J.: 755, 3555
Spicher, J. M: 3325-162
Spiers, G. F: 1325-67
Spilsbury, K.: 1290, 2020
Spini, D.: 1385-213, 3315-123
Spira, A. P: LB2570-382
Spiro III, A.: 3340-206, 3370-301, 3575
Spoden, N.: LB935-436
Spoelstra, S. L: LB1545-433
Spradley, L.: LB935-420
Sprague, B. N: 1150, 3290-63
Sreenivas, R. S: LB1545-373
Srikumaran, U.: 2360-83
St. George, S. M: 3315-130
St. John, P.: 870-185
St. Louis, R. M: 1410-331
Staben, O. E: LB2570-402
Stadler, D.: 3545
Stagg, B. C: 2495
Stahl, S.: 3150, LB2570-324
Stallard, E.: 820-33, 825-60
Stanback, B. M: 2435-308
Stanely, M.: LB2570-420
Stange, K.: 1401-276

Stanley, A.: 900-243
Stanley, J.: LB935-366, LB3620-326
Stanley, J. T: 815-8, 1385-204
Stanley, J. L: 3315-122
Stanmore, E. K: 730
Stapleton, H.: LB2570-322
Stark, S.: 3270-3
Starr, L. T: 840-109
Starr, M. E: LB3620-387
Statler, A.: 1395-243
Statz, T. L: 1385-210, 2375-140
Stavrinos, D.: 1410-327
Stawski, R. S: 1350-125, 3030, 4035
Stearns, M.: 3320-148
Steedley, M.: 1055
Steele, C.: LB1545-377
Steele, J. C: 1385-203
Steeves-Reece, A.: 1305-27
Steffens, D. C: 3010, 3360-263
Stein, G. L: 3225
Steiner, V.: 1345-110, 1355-147, 2355-73
Steinheiser, M.: 3345-230
Steinman, B. A: 895-236
Steinman, L.: 810-3
Stempel, C.: LB935-363
Stengel, J.: 2415-268
Stensland, M.: 2010
Stephenson, E.: 2530
Stepniak, C.: 3315-131
Steptoe, A.: 730, 2370-128
Sterling, M.: 1155
Stern, Y.: 3290-65
Sterns, H. L: 2100
Stessman, J.: 2315-9
Stevens, A. B: 2300, 3320-136
Stevens, G.: 2385-188
Stevens, J. D: 1445
Stevenson, B.: LB1545-353
Steward, A.: 905-252
Stewart, J.: 2360-102
Still, C. H: 770
Stinchcombe, A.: 1380-198, 1410-329, 2550, 3595
Stine-Morrow, E.: 3290-56
Stirland, L.: 3210
Stirling, A.: LB935-427
Stocker, R.: 2395-213, 4055
Stockmeier, C. A: 1330-84
Stohrer, H.: 800
Stokes, J. E: 725, 1315-54, 1380-201, 3150, LB2570-413
Stolee, P.: 3330-180
Stone, K. L: 2125
Stone, L. E: 915-283, 1390-226
Stone, P. W: 2395-217
Stone, R.: 1155, 2015, 2515, 3140
Stoop, A.: 635
Stough, C.: 3295-81
Stow, D.: 1200
Stoyles, S.: 1360-160
Straatmann, V.: 3420
Straker, J.: 2385-183, 3555
Strasser, I.: 2390-206
Strath, S.: 2410-258
Stratton, L.: 2390-202, LB1545-385, LB3620-356
Stratton, S.: LB935-377
Strecher, V.: 1425
Street, D. A: 3000, 4125
Streeter, J. L: 3410
Streiff, J.: 1401-271
Stripling, A. M: 915-298, 1380-192, 1405-321, 3370-287
Strogatz, D.: 3325-171
Strommen, J.: LB3620-441
Strong, J.: 535, 3125
Strong, J. V: 3290-58
Strotmeyer, E.: 565, 2445
Strough, J.: 3370-298
Stroustrup, N.: 1115
Struble, L. M: 850-127
Struckmeyer, K. M: 3320-145
Stubbings, G. M: 825-55
Stucki, B.: 3290-62
Studenski, S.: 705, 820-38
Studenski, S.: 2360-94
Studenski, S.: 3075
Studenski, S.: 3295-71
Stum, M.: 2035, 2235, LB2570-376
Stuppard, R.: 835-101
Stupplebeen, D.: LB2570-421
Sturdevant, D. L: 3550
Su, M.: LB935-408
Suarez, J.: 850-134
Subedi, J.: LB935-402
Sublett, J. F: LB3620-406
Suchting, R.: 2365-114
Suchy, Y.: 1315-48
Sugawara, I.: 1345-109, 3340-199
Sugiyama, T.: 4005
Suitor, J.: 2160, 3315-131
Sullivan, D.: LB2570-425
Sullivan, J. L: 3255
Sullivan, K. J: LB2570-401
Sullivan, M.: 1535
Sullivan-Marx, E.: LB1545-335
Sultana, A.: 2400-230
Sultana, A.: 2360-98
Summerson, E.: 3365-277
Sun, A. Y: LB3620-396
Sun, F.: 500, 2313, 3250, LB935-432, LB2570-410
Sun, H.: 3480
Sun, J.: 1297
Sun, L.: LB2570-352
Sun, L.: LB3620-384
Sun, N.: 4150, LB935-402
Sun, Q.: 3250
Sun, W.: 3355-252
Sun, X.: LB935-408, LB1545-446
Sun, Y.: LB935-380
Sun, Y.: 820-32Sung, A. D
Sunderaraman, P.: 3290-65
Super, N.: 2325-23
Supiano, K.: 2325-24
Supiano, K.: 500, LB2570-416
Supiano, K.: LB935-358
Supiano, M.: 4045
Suppiah, S.: 3355-251
Surachman, A.: 1440
Susana-Castillo, S.: LB935-416
Sussman, J. C: 2385-173
Sussman, T.: 910-264, LB1545-439
Sutherland, L.: 890-222
Sutherland, L.: 1075
Sutphin, G. L: 3195
Suwalski, M. W: 1360-157
Suzuki, H.: 855-154
Suzuki, N.: LB1545-344
Suzuki, R.: 1360-161
Svynarenko, R.: 3315-132
Swall, A.: 3295-86
Swan, A. A: 3295-76
Swann, L.: 2370-134
Swanson-Aprill, L. A: 3305-109
Sward, K.: LB935-400
Swartzendruber, L.: LB2570-425
Sweeder, L. A: 3295-89
Sweeney, C.: LB3620-380
Sweigart, B.: 2345-55, 3215
Sweitzer, S.: 2360-86, LB935-441
Swenor, B.: 665, 3295-73
Swierenga, S. J: 3305-109, 3415, 4020
Swift, A.: 870-185
Swift, H.: 3000
Switsers, L.: 3035
Syed, S. U: LB2570-392
Syme, M.: 1485, 2515, LB1545-407
Szanton, S. L: 515
Szanton, S.: 920-305
Szanton, S. L: 1510, 3270-3
Szanton, S. L: 3360-268, LB2570-353
Szanton, S.: LB2570-382
Sze, L.: 2400-227, 2425-287, LB3620-440
Szkody, E.: 915-292
Szychowski, J.: 3345-211
Szymkowski, A.: LB1545-400

### T

Taani, M. H: 2410-258
Taeckens, A.: 845-116
Taffet, G. E: 2190
Taguchi, A.: 885-205
Taguchi, J.: 520
Taitel, M.: 1360-157
Takahashi, K.: 3325-157
Takahashi, P.: LB3620-365
Takaoka, M.: 895-225
Takase, M.: 925-330, 3270-4
Takata, Y.: 2405-237
Takayama, M.: 1345-109
Takayama, M.: 3340-199
Talab, A.: LB1545-340
Talebzadeh, A.: 2360-98
Talley, K. M: 2380-155
Talwar, S.: 2420-276
Tamashunas, N. L: LB1545-355

Tamayo, L.: 2250
Tamimie, E.: LB935-395
Tamiya, N.: LB2570-444
Tan, A.: 3275-26
Tan, C.: 3250
Tan, P.: 2360-96
Tan, S.: 3475
Tan, Y.: 3355-251
Tanaka, T.: 895-232, 3270-4
Tanaka, T.: 3325-157
Tanaka, T.: 830-63
Tang, F.: 2345-54
Tang, F.: 640, 4105
Tang, J.: 915-294, 1345-115, 2425-287, 3075
Tang, J.: LB3620-440
Tang, V. L: 2315-1
Tang, W.: LB935-421
Taniguchi, Y.: 3340-204
Tanjong-Ghogomu, E.: 3415
Tanksley, B.: 795
Tanzi, R.: 825-51
Tarabochia, D. S: 2430-304
Tarbert, R. J: LB2570-397
Tarbi, E.: 870-179
Tardif, S.: 525
Tarleton, E.: 2360-89
Tarraf, W.: 860-159, 1360-164, LB3620-342
Tarter, R.: 840-104
Tasnim, S.: 2400-230
Tate, A. M: LB3620-381
Tate, K. C: 2315-15, 2395-219, 4130
Tate, R. B: 870-185
Tavares, C.: 3535
Tavares, D.: 3365-281
Tavares, J. L: 865-163, 1095
Tavener, M.: 2410-251
Tavenier, J.: 3280-32, LB2570-339
Tay, P. K: 2360-96
Tayade, A. S: 630
Taylor, C.: 2410-249
Taylor, C.: 3465
Taylor, C. A: 1030
Taylor, C. A: 1315-55
Taylor, C. A: 4110, LB935-362
Taylor, E.: 3180
Taylor, H. O: 1145, 3065, 4150
Taylor, H.: LB2570-421
Taylor, J. L: 775
Taylor, L.: 2365-106
Taylor, L.: 3490
Taylor, M. G: 1160, 1400-252, 2550
Taylor, P.: 510, 1540
Taylor, S.: 3390
Taylor, T. N: 2485, 3525
Taylor, Y. J: LB2570-327
Teas, E.: LB2570-408
Teaster, P. B: 1265, 2035, 2430-293, 3025, 3345-223, 3515
Teichmann, B.: 3375-310
Teixeira, D.: 2360-87
Teixeira, L.: 880-201
Tejada, L.: 1380-197
Telonidis, J.: LB2570-416
Temkin-Greener, H.: LB1545-381
Templeman, M.: 2435-311
ten Brinke, L. F: 715, 3295-72
Ten Cate, D.: 675, 2380-158
Teng, A.: LB935-445, LB1545-358
Teno, J.: 520
Tepper, L.: LB1545-374
Terada, T. M: LB3620-374
Teres-Vidal, L.: 3345-216
Terhune, K. N: 680
Teri, L.: 1415, LB935-436
Terman, G.: LB1545-412
Terracciano, A.: 3575
Terrana, K.: 3200
Terrin, M.: LB1545-424
Terry, E.: 775
Testad, I.: LB3620-317
Teuber, I.: 2340-47
Teufel-Shone, N.: LB2570-445
Thao, M.: 3345-217
Thapa, S. S: LB935-402
Theng, Y.: 885-210
Theou, O.: 565, 3325-165, 3590, 4045
Thiamwong, L.: 2360-88, LB935-413
Thierry, A.: 1485, 3095, 4120
Thill, L.: 920-314
Thoma, M. V: 1330-88
Thomas, A.: 3310-117
Thomas, F.: 3305-102
Thomas, G.: 3350-240
Thomas, K. S: 705, 1305-33, 1375-187, 2470, 3140, 3345-215, 3435, 4135
Thomas, K.: 2265
Thomas, M.: 1535
Thomas, P. A: 725, 875-187, 3510
Thomas Tobin, C. S: 1050, 3475
Thomasson, W.: LB935-420
Thomeer, M.: 2160
Thompson, E. H: 870-185, 2415-268, 3275-15
Thompson, H. J: 2310
Thompson, J. L: LB935-390
Thompson, J.: LB1545-354
Thompson, K.: 3305-108
Thompson, L. V: LB2570-325
Thompson, L.: LB935-352
Thompson, M. D: LB935-426
Thompson, M.: LB1545-420
Thompson, N. A: LB1545-431
Thompson, R. A: LB3620-349
Thompson, S.: 3360-258
Thorp, J.: 3345-224
Thorpe, C.: 3390
Thorpe, J.: 3390
Thorpe, R. J: 1050, 1145, 1450, 3360-260, 3360-268, 3475
Thorpe, S.: 1401-281
Thorsdottir, I.: 2370-131
Throckmorton, C.: 1402-308
Thrush, K.: LB2570-429
Thunell, J.: 4155
Thurlow, C. B: 4170
Thurman, W.: 3600
Thyagarajan, B.: 710, 2260, 2345-57, 2370-125, 3170
Thyne, K.: 820-35, 830-77, 835-97
Tiainen, K.: LB1545-339
Tian, J.: 1410-328
Tian, Q.: 3075, 3295-71
Tian, Y.: LB3620-347
Tice, J. L: LB1545-395, LB3620-322
Tilton, N.: LB2570-389
Timmerman, K.: LB2570-408
Tinetti, M.: 3560
Tipirneni, R.: 540
Titler, M.: 850-127
Tiwari, A.: 3295-89
Tkatch, R.: 1015
Todd, A.: LB1545-418
Todd, C.: 730
Todd, O. M: 1360-165
Todd-Magel, C.: LB1545-369
Tofthagen, C.: 1330-93
Togonu-Bickersteth, F.: 890-220
Togonu-Bickersteth, F.: 1355-149
Togonu-Bickersteth, F.: 3300-100
Tokashiki, T.: 3280-30
Tolea, M. I: 3295-85
Tom, S.: LB1545-410
Tomita, M. R: 925-326, 3175
Tompkins, C. J: 3305-103, 4055
Tong, C.: 3330-180
Tong, H.: 2030
Tonkikh, O.: 3420
Toosizadeh, N.: 820-26
Torain, J. M: 3330-188
Toral-Garcia, J. J: LB3620-379
Toril, P.: 3370-290
Torossian, M.: 1395-249, LB2570-372
Torrence, M.: LB1545-362
Torres, G. R: 730
Torres, S.: 1070, 1210
Torres, S.: 3015
Torres-Gil, F.: 2400-224
Torsvik Henriksen, A.: LB3620-317
Toseef, M.: LB3620-342
Tovar, A.: LB2570-422
Towne Jr., S. D: 3300-96
Towns, J.: 2405-241
Townsend, A.: 1390-227
Toyama, M.: 3205
Toye, C.: 505

Toyokawa, N.: 1350-128, 2000
Toyokawa, T.: 1350-128
Tracy, E.: LB935-400
Tracy, T.: LB935-349
Trageser, K.: 825-58
Tramm, G.: 1385-205
Tramujas Vasconcellos Neumann, L.: 3415
Tran, V.: 1330-82
Trapuzzano, A.: 920-308
Travison, T.: 1402-302
Trejo, L.: LB3620-444
Trinh-Shevrin, C.: 850-131
Triolo, F.: 515
Tripp, B.: 825-48, 4130
Tripp, P.: 3320-145
Trivedi, A.: 2325-21
Trivedi, R.: 1170, 3535
Troutman, S.: 3290-63, 3295-69
Troy, L. M: 715
Troya, Y.: 900-246
Trubenstein, B. P: 1150
Trudeau, S. A: 1295
Tsai, H.: 2385-182
Tsai, Y.: 805
Tsay, T.: 2360-92
Tse, M.: 1345-115
Tseng, C.: 2380-157
Tseng, C.: LB935-406
Tseng, G. C: 3275-24
Tseng, T.: 1040
Tseng, W.: LB2570-381
Tsotsoros, C.: 865-166
Tsukada, N.: 2320-16
Tu, Y.: 1300-7
Tu, Y.: 1401-288
Tuchaai, E.: 820-32
Tulner, L.: 1365-175
Tumosa, N.: 1020, 2075, 4075
Tunalilar, O.: 910-277, 3060, 3345-214, LB2570-404
Tung, K.: 2375-152
Turiano, N. A: 1440, 3350-243
Turner, A. N: LB2570-371
Turner, J. R: 815-8
Turner, K. W: 1315-50
Turner, S.: 1350-125, 3235, 3340-200, 3370-300
Turner II, R. W: 2325-29, 3205
Turney, I. C: 2455
Turturro, C. L: LB3620-434
Tuzzio, L.: LB2570-368
Tworek, G.: 1330-82
Tyler, D.: 2385-180, 3280-35
Tzeng, H.: 2385-172

U

Uchida, H.: 1390-220
Udeh, K.: 820-40, 3385
Ukraintseva, S.: 825-60, 1310-42, 2370-132, LB1545-440, LB2570-338
Ulack, C.: 850-134
Ulatowska, H. K: 3535
Ullah, M.: 1310-34
Ullmann, G.: 3290-51
Um, T.: LB2570-378
Umberson, D.: 2160, 2280
Umezaki, Y.: LB1545-344, LB2570-320
Ungar, N. L: 4015
Ungar, R.: 1015
Unson, C. G: 865-164
Unutzer, J.: 3010
Upchurch, G.: 1305-24
Upenieks, L.: 515, 725
Upenieks, L.: 925-329
Upenieks, L.: 1345-108
Uphold, C. R: 840-108, 3320-137
Urban, K.: 2385-193
Urbanek, J.: 820-24, 2360-92
Ureña, S.: 1400-252
Utz, R.: 500

V

Vadlamani, A.: 2370-130
Vagle, V.: LB3620-317
Vailati Riboni, F.: 2340-38
Valdez, H.: 890-223, 1095
Vale, M. T: 1380-200
Valencia, A. P: 835-101
Valencia, G.: 835-101
Valencia, W.: 1320-61, 1330-94, 1402-299, 2350-59
Valenzano, D.: 525
Valera Gran, D.: LB935-430
Valiani, V.: LB3620-403
Valigosky, M.: 1345-110
van Aalst, R.: 4130
Van Alphen, J.: 3230
van Asch, I.: LB1545-415
van belzen, E.: LB1545-415
van de Pol, M.: 1365-176
van der Horst, M.: 3000
Van Der Most, R.: 1135
van der schot, A.: LB1545-414
van der Steen, J. T: 4135
van der Valk, A.: 3590
van der Velden, C.: 840-110, LB1545-415
van der Vorst, A.: 3270-5
Van Dussen, D. J: 1360-153
van Erp, J.: LB1545-414
Van Haitsma, K.: 785, 845-119, 2525, 3255
van Hout, H.: 635
Van Houtven, C. H: 880-194, 1110
Van Orden, K.: 910-275
Van Skike, C. E: 825-44, 1310-38
Van Vleet, S. C: 2340-44
van Weert, J.: 1365-176
Vanajan, A.: 660
VanCleave, J. H: LB3620-443
Vandenberg, A. E: LB2570-351, LB3620-325
Vandenbosch, J.: 2145
Vanderboom, C.: 3180
Vandiver, R.: LB3620-367
Vanegas, D.: 3315-130
Vang, C.: LB2570-316
VanHaitsma, K.: LB1545-397, LB2570-358
Vardarli, E.: 920-310
Varey, S.: 2310
Vargas, I.: LB3620-371
Vargas-Chanes, D.: 4155
Vasquez, E.: 2345-54, 2370-124, 3325-163
Vasquez, P.: 3360-266
Vasunilashorn, S.: 825-48, 4130
Vattigunta, S.: 2360-83
Vaughan, C.: LB3620-325
Vaughan, M. W: 2385-190
Vaughn, I. A: 3330-184
Vayndorf, E.: 830-81
Vazquez Arias, M.: 3375-302
Vecchione, K.: 3345-229
Veerbeek, M.: 840-110
Vega-Lopez, S.: 890-223, 1095
Vega-Soto, S.: 890-223, 1095
Veghte, B.: 3015
Velasco Roldan, N.: 2385-179, LB935-353
Velasco Roldan, N.: LB3620-359
Velez Ortiz, D.: 2400-228
Vellani, S.: 3415
Vellas, B.: LB2570-367
Velsey, K.: 2360-90
Venditelli, R.: LB2570-355
Venditti, E. M: LB1545-432
Venema, D.: 3295-80
Verbeek, H.: 785, LB1545-414
Vereen, S.: LB3620-426
Verloo, H.: 3495
Verma, A.: LB935-380
Vernon, A.: 3320-136
Versey, S.: 595
Vest, L.: 2215
Vetrano, D.: 515, 3170, 3325-173
Vetrano, D.: 3170
Viana, J.: 1402-307
Vickers, J. K: 2315-4
Vickerstaff, S. A: 510, 3000
Vicki, W.: LB2570-391
Victor, C.: 650, 1535
Videon, T. J: 850-143
Vidoni, E.: 3175, LB935-437
Vieira, E. R: 1402-312, 2360-85, 3325-170, 3365-281
Vieira Zamora, F.: LB3620-391
Vigen, C.: 890-213
Viger, M.: 2355-62
Villa, V.: 3015
Villeda, S.: 3080
Vinales, K.: 3325-176
Vincenti, V. B: 1055, 2035
Vincenzo, J. L: LB3620-416
Vinkers, C.: LB1545-422
Violán, C.: 3170

Virnig, B.: 2390-200
Visaria, A.: 865-167
Vitaliano, P.: 1345-113
Vitiello, M. V: 1510
Vivek, S.: 710, 2345-57
Viviano, N. A: 920-301
Vivoda, J.: 1305-21
Vo, T.: 925-338
Voaklander, D.: 850-126
Voelkner, A. R: 2340-41
Vogel, C. E: 915-295
Vogelsmeier, A. A: 4135
Voisine, M.: 2345-55
Volandes, A.: 3555
Volpi, E.: LB935-378, LB2570-328, LB3620-345
von Humboldt, S.: 930-339
Vondracek, H.: LB2570-419
Voss, M.: 660
Voss, M.: LB935-350
Vrahas, M.: 3420
Vutukuri, S.: LB2570-365

### W

Wachowiak, P. L: LB2570-345
Wack, K.: LB2570-351
Wadsworth, S.: 1150
Wagen, B.: 930-343
Wagg, A.: 850-126
Wagner, M.: LB3620-318
Wagner, R. G: 4110
Wagner, S.: 825-51
Wagstaff, R. A: 1315-49
Wahler, R.: 3330-187
Waite, L. J: 2425-291, 4070
Wakefield, B.: 4135
Wakefield, D.: 2045, 3360-263
Wakui, T.: 880-202, 2375-151, 3145, LB2570-444
Waldron, D.: 2185, 2530
Waldrop, D. P: 855-150, 880-195, 2385-181
Waldrup-Valverde, D.: LB3620-379
Wales, K.: 2410-251
Walker, C.: 2270
Walker, D.: 2355-63
Walker, L.: 1325-74
Walker, M.: 925-328
Wall, R.: 1401-282
Wallace, D.: 2315-8
Wallace, F.: 635
Wallace, L. M: 2095
Wallace, M.: 890-216, 2385-177, 3320-141
Wallace, S.: 850-134
Walsh, C.: LB1545-439, LB2570-424
Walsh, C.: 1350-132
Walsh, D.: 3535
Walsh, D.: 910-257
Walsh, E.: 2415-269
Walsh, K.: 560, 2105, 4055
Walsh, S. E: 895-237, LB2570-427
Walston, J.: 820-34, LB3620-364
Walton, R.: 1400-267
Wan, J.: LB2570-439
Wan, Y.: LB935-373
Wang, B.: 825-47
Wang, B.: 615, 3505
Wang, C.: 2360-99, LB935-381
Wang, C.: 3325-160, LB3620-372
Wang, F.: 1355-144, 2405-232
Wang, G.: 2385-175
Wang, H.: 835-93
Wang, H.: 2410-262, LB935-400
Wang, H.: 915-297, 1315-54, 3315-129
Wang, J.: 3305-105
Wang, J.: 4115
Wang, J.: 4105, LB1545-380
Wang, J.: 740
Wang, K.: 1320-61, 1330-94, 1402-299, 2350-59
Wang, K.: 500, 3250
Wang, K.: 3275-25
Wang, K.: 3295-83
Wang, L.: 3030
Wang, L.: 910-271
Wang, M.: 3520
Wang, Q.: 2415-267, 3200
Wang, S.: LB2570-387
Wang, S.: 1315-60, 1400-268, 2420-270, LB935-355
Wang, S. Y: 1390-232
Wang, X.: 910-269
Wang, X.: LB2570-441
Wang, Y.: 4135
Wang, Y.: 4150
Wang, Y.: 810-1
Wang, Y.: 3250, 4150
Wang, Y.: 2340-43
Wang, Y.: 910-267
Wang, Y.: 910-272
Wang, Y.: 890-219
Wang, Y.: 1340-101, LB2570-366
Wang, Z.: 1355-137
Wang, Z.: 2435-312
Wang, Z.: 820-25
Wanigatunga, A. A: 1402-296, 2125, 2360-92, LB935-446
Wank, M.: LB2570-435, LB3620-378
Wanka, A.: 575
Warburton, J.: 1385-206
Ward, C.: LB3620-364
Ward, D. V: 820-29
Ward, M.: LB3620-359
Ward, R.: LB1545-363
Ward, R.: LB2570-408
Ward, S.: 710
Ward, S.: 2425-280
Warden, K.: 1360-161
Ware, O.: LB3620-355
Waris, A.: LB2570-373
Warner, D. F: 3070
Warraich, H.: 870-182
Warren-Clem, K.: 2385-174
Washburn, A. M: 2505
Washington, K. T: 1080
Washington, T.: LB3620-375
Washington, T. R: 770, 1160, 1401-287, 1485
Wasiak, M. F: 3520
Waszynski, C.: 2315-5
Watanabe, T.: LB2570-444
Watanabe, Y.: LB1545-346
Watanabe, Y.: 895-232
Waters, L.: 1530, 3300-94, 4075, 4170
Watkins, J.: 1055
Watkins-Dukhie, K.: 3430
Watson, K.: LB935-442
Watts, A.: 500, 2410-250, 3175
Waycott, J.: 1385-206
Weaver, A. A: LB1545-390
Weaver, B.: 1410-329, 2550
Weaver, R. H: 1305-26, 2385-189, 2435-314, 3345-223
Webb, C.: 3290-61
Webb, C.: LB3620-334
Weber, K.: 2375-143
Webster, B. A: 1350-129, 1460
Webster, N. J: 745, 3205, LB3620-400
Weech-Maldonado, R.: 910-262, 2185, 2250, 2395-218, 3345-213, 3435
Wehlandl, E.: 2360-101
Wehr, H.: 810-2
Wehry, S.: LB3620-350
Wei, J.: LB3620-369
Wei, L.: 2370-128
Wei, M. Y: LB2570-389
Wei, S.: 780
Wei, W.: 3320-142
Weil, J.: 2505, 3140
Weimert, J.: 2360-101
Weiner, B.: 520
Weiner, D.: 3280-33
Weinreich, D.: 2100, 3005
Weintraub, S.: 830-76
Weir, M.: 2380-157
Weiss, D.: 3380
Weisse, C.: 3375-312
Welch, S. A: LB2570-380
Welch, V.: 3415
Wellar, H.: 3290-45
Wells, K.: LB935-392
Wells, M.: 3295-76
Wells, Y.: 2395-210
Welsh, D.: LB1545-417
Wen, J.: LB935-411
Wendel-Hummell, C. L: LB2570-425
Wendell, S. G: 1425, 3275-24
Weng, T.: LB935-350
Werner, C.: 920-306
Wernher, I. A: 2325-27
Wernli, K.: LB2570-368
West, J. S: 805, 2410-263
West, W. G: 2380-155
Westbrook, R.: LB3620-364
Westra, J.: 3355-250
Wetherell, J. L: 4080
Wetle, T.: 880-194

Wetstone, R.: 3315-130
Weuve, J.: LB1545-410
Wharton, T. C: 1320-62
Wharton, T.: 2313
Wharton, W.: 895-238
Wheaton, F. V: 1400-254, LB3620-423
Wheeler, V.: 2370-136
Whitaker, E.: 3295-89
White, A.: 740, 3330-179
White, D.: 910-277, 3345-214, LB2570-404
White, H.: 1305-24
White, K.: 1305-28, 4055
White, R.: 1400-257
White, T.: 830-67, 835-84
Whitehead, B.: 2410-247
Whitehead, I.: LB1545-393
Whitehouse, C. R: 1170
White-Ryan, L.: LB2570-340
Whitfield, K. E: 3130, 3290-53, 3360-260, 3475
Whiting, C.: 1030, 1230
Whitlatch, C.: 1160, 2245
Whitley, D.: 3245
Whitlow, R.: 2405-231
Whitmire, M.: 760
Whitsel, E.: LB2570-441
Whitson, H. E: 2240, 4130
Whitson, J.: 835-101
Whittaker, A. C: LB935-398
Whittier Eliason, S.: 2385-193
Wicklander, K.: LB1545-412
Widen, S.: LB1545-354
Wiener, C.: 2550
Wierenga, K.: 1330-75
Wiese, L. K: 2500
Wilber, K.: 2375-150, 3025
Wilckens, M. R: 3520
Wild, E.: 3180
Wild, K.: 1401-282, 3535, LB1545-430
Wiley, J.: 3330-190
Wilkens, L. R: LB935-428, LB1545-392
Wilkerson, T.: 2425-280
Wilkinson, L. R: 885-209
Wilkinson, S.: 3010
Wilks, S. E: 2385-192
Willcox, B. J: 830-72
Willcox, C.: 830-72
Willemse, B.: 840-110
Willett, W.: LB2570-367
Williams, A.: 910-278
Williams, A. R: LB3620-366
Williams, B.: 2425-282
Williams, C.: 740, 3330-179
Williams, C.: 3295-86
Williams, K. A: 3430
Williams, K. N: 850-133, 1495, 2355-74, 3550, 3560, LB2570-364
Williams, N.: 3275-26
Williams, R.: 1490
Williams, S.: 915-290
Williams, W.: 930-343
Williams-Blangero, S.: LB935-402
Williams-Farrelly, M.: 730
Williamson, E. A: 3315-120
Williamson, J.: 4045
Willie-Tyndale, D.: 3045
Willink, A.: 665, 4155
Willis, S.: 2265, 2410-259, 3290-47
Willoughby, L.: 2405-238
Willoughby, L.: 3350-235
Wilmoth, J. M: 1145, 2055, 2375-145
Wilson, C.: LB3620-369
Wilson, D. A: LB935-438
Wilson, D. M: 3285-40
Wilson, J.: 885-203, 3370-298, LB2570-360
Wilson, K.: 1380-198, 2250
Wilson, L. M: 2380-156
Wilson, R. S: 3480
Wilson, S. J: 2275, 2420-275
Wilson, T. L: 2385-183
Wilson-Frederick, S.: 2250
Wilton, S.: 3275-15
Winblad, B.: 2215
Winchester, B. S: 1505
Windham, G.: LB2570-401
Windsor, T. D: LB935-387, LB1545-445
Winfree, J.: 910-277, 3345-214, LB2570-404, LB3620-390
Wingood, M.: 2360-89
Winningham, R.: 2310
Winterton, R.: 560, 3285-41
Wion, R.: LB2570-358
Wisniewski, K. M: LB2570-350
Wisniewski, M.: LB2570-435, LB3620-378
Wister, A.: 3285-39
Witkowski, S.: 715
Witsaman, R.: LB935-416
Wittich, W.: 850-142
Wladkowski, S.: 4170
Wohlgemuth, W.: LB3620-338
Wöhrmann, A. M: 3520
Wojczynski, M. K: 1310-42, 3170, LB2570-338, LB3620-337
Wolf, M.: 3595
Wolf, M.: LB1545-430
Wolf-Ostermann, K.: 785, 2020
Wolfe, A.: 920-310
Wolfe, J.: 2360-101
Wolfe, R.: 870-180
Wolff, J. L: 1110, 1170, 1495, 2045, 2325-25, 3420
Wolff, J. K: 3615
Wolfram, A.: 2415-269
Wong, A.: 1390-229
Wong, C.: 2360-96
Wong, G.: 2400-227
Wong, G. H. Y: 845-121, 915-294, 2425-287, 3075, LB1545-368, LB3620-440
Wong, J.: 850-131, 3360-265
Wong, J. D: 3180, 4120
Wong, R.: 3220, 4155, LB1545-345
Wong, R.: 2355-81
Wong, S.: 3335-195
Wong, T.: 3605
Woo, B.: 3250
Woo, J.: 2425-286
Wood, K. A: 1350-134
Woodard, J. L: LB3620-389
Woodham, S.: 1170
Woodhouse, M.: 2155
Woods, N. F: 1455
Woods, R. L: 870-180, 3260
Woods, T.: 1365-168
Woodspring, N.: 1385-211, 3155
Woodward, A.: 3415, 4020
Woodward, J. M: LB2570-327
Woog, S.: 780
Wool, J.: LB935-396
Wooten, K.: LB935-362
Worthalter, D.: 3310-116
Wortman, E.: 2405-243, 4160
Woulfe, K.: LB1545-402
Wrangham, R.: LB1545-431
Wray, L. O: 2015, 2535
Wray, L. A: 4120
Wright, E.: 1330-81
Wright, K. D: 845-117
Wright, R.: 515
Wright, R. S: 655
Wright, T.: 3305-102
Wright-Freeman, K.: 780
Wroolie, T. E: LB935-442
Wu, B.: 1105, 1297, 1340-103, 1435, 2340-43, 3090, 3160, 3315-129, 3320-142, 3495, 1300-1
Wu, B.: LB3620-342
Wu, C.: 805, 4045
Wu, C.: 820-32
Wu, D.: 825-60, LB1545-440
Wu, H.: 4145
Wu, J.: 2475
Wu, M.: LB935-429
Wu, S.: LB935-429
Wu, S.: 910-267
Wu, S.: 2475, 4005
Wu, S.: 910-267
Wu, Y.: 3160, 3290-54, LB935-426, LB1545-392
Wu, Y.: 4040
Wu, Z.: 4030
Wyatt-Brown, A.: 1185
Wylie, M. J: 3110
Wyman, M. F: 2015

### X

Xiang, A.: 3175
Xiang, X.: 2495, 4150
Xiao, Y.: 3335-193
Xie, B.: 2165, LB2570-330
Xie, B.: 3590
Xu, B.: 1330-77, 2370-137, 4005
Xu, B.: LB935-381
Xu, F.: 1170, LB2570-422
Xu, H.: 1105
Xu, H.: LB2570-315
Xu, H.: 2400-229
Xu, L.: 910-263, 2340-46, 2365-111, LB3620-405
Xu, M.: 4110
Xu, S.: 915-297
Xu, X.: 1305-30, 1350-122, 3230
Xu, X.: 840-106
Xu, Y.: LB2570-385
Xu, Y.: 895-233
Xue, L.: 565
Xue, Q.: 830-75, 2095, 4165
Xue, T.: LB2570-385

### Y

Yadav, H.: LB2570-387
Yaffe, K.: 3075
Yamaguchi, M.: LB1545-344
Yamaguchi, T.: 885-205
Yamaguchi, Y.: 830-63
Yamamoto-Mitani, N.: 895-225
Yamasaki, J.: LB1545-337
Yamashita, T.: 510, 1325-73, 1385-215, 1400-259, 1401-290, 2010, 3300-99, 3330-191
Yamauchi, K.: 830-63
Yamin, S.: 840-114, 3595
Yan, E.: 1355-143, 2320-17, 2375-152
Yan, M.: 1305-17
Yan, S.: 1315-58
Yanai, S.: LB2570-411
Yancura, L.: 1460
Yang, B.: 2370-121, 2390-198
Yang, E.: 1365-173
Yang, F.: 825-42
Yang, F.: 1515
Yang, H.: 830-74
Yang, H.: 2410-246
Yang, J.: 3325-156
Yang, J.: LB935-381
Yang, J.: 865-176, 1380-191
Yang, K.: LB935-433
Yang, M. S: 2400-221
Yang, M.: 2385-182
Yang, R.: LB935-400
Yang, W.: 1375-186, 3395
Yang, X.: 2005
Yang, X.: 705
Yang, Y.: 925-319
Yankeelov, P.: 1345-107, 1365-167, 2355-76, 3300-90
Yao, L.: LB1545-340
Yao, N.: LB935-408
Yao, S.: 1330-77, 2370-137, 4005
Yap, T.: 3495
Yarbro, J.: 835-85
Yarbrough, M.: 2270
Yashin, A.: 825-60, 2370-132, LB1545-440
Yashkin, A.: 825-60
Yashkin, A.: 2370-132
Yasunaga, M.: 855-154
Yasuzato, C.: 3300-91
Yates, T.: 1355-145
Yazdani, N.: 3290-49
Ye, L.: 3330-185
Ye, M.: 1500, 3345-231, 4160, LB2570-383, LB3620-354
Ye, Z.: 1300-1, 3160
Yeager, K.: 1510
Yee, B.: 2140
Yefimova, M.: 2465
Yeh, C.: 665, 1015, 1315-60, 1400-268, 1425, 2380-166, 2420-270, LB935-355
Yeh, C.: 835-93
Yeh, J. C: 595, 2540, LB935-377
Yeh, S.: 1401-288
Yen, K.: LB2570-439
Yentes, J. M: 3295-80
Yeom, H.: 2340-51
Yeung, D.: 865-171, 1325-69, 3370-293
Yeung, K.: 895-233
Yi, E. G: 880-200
Yin, C.: 1315-50
Yin, L.: 1095
Yip, A.: LB1545-341
Ylitalo, K.: 2410-248, 2445
Yonashiro-Cho, J.: 2370-118
Yonashiro-Cho, J.: 3025
Yoneda, M.: LB1545-344
Yoneda, T.: LB2570-347
Yong, M.: 4155
Yoo, J.: 3315-121
Yoon, E.: LB3620-420
Yoon, H.: 615, 1325-70
Yoon, J.: 1325-72
Yoon, J.: 1402-305
Yorgason, J. B: 865-168, 1401-277, 3185, 3290-45
York, A. M: 2305
Yoshikawa, A.: 3300-96
Yoshinaga, F.: 3375-314
Yoshino Benavente, J.: 3595
You, L.: LB3620-403
You, T.: 3220
You, Y.: 1340-102
Youkhana, F.: LB935-426, LB1545-392
Younan, D.: LB2570-441
Young, H.: 2295, 2440, 3450
Young, M. A: 1305-18
Young, T.: LB3620-414
Young, Y.: 2155, 2385-182, 4165
Yous, M.: 1395-245
Yousefi Nooraie, R.: 2130
Yow, W.: LB1545-388, LB2570-344
Yu, C.: 1330-85
Yu, D.: 845-120, 4080
Yu, F.: 3175, LB1545-380
Yu, G.: 825-45
Yu, H.: 1355-146
Yu, H.: 910-267, 1300-7
Yu, K.: 1060, 2475
Yu, Q.: LB935-366
Yu, R.: LB1545-365
Yu, S.: 1345-116
Yu, T.: 2385-182
Yu, W.: 1402-302
Yu, Y.: 825-42
Yuan, L.: LB1545-446
Yue, L.: LB3620-319
Yuen, B.: 2360-95
Yun, J.: 890-215
Yun, J.: LB2570-432
Yun, S.: 830-78
Yun, S. W: 2355-68
Yuri, A.: 925-319
Yusifova, M.: LB2570-346

### Z

Zaccaria, D.: 2425-281
Zahiri, M.: 1402-297, 2360-99, 3325-156
Zahodne, L. B: LB1545-410
Zaia Rodrigues, A.: 820-25
Zaidel, C.: 2420-270
Zakrajsek, A. G: LB2570-342
Zakrajsek, J. S: 1410-326
Zambrano Garza, E.: LB3620-383
Zammit, A. R: LB3620-318
Zang, E.: 3145
Zaninotto, P.: 730, 2370-128
Zanjani, F. A: 2425-280, 3300-94
Zanjani, F.: 3515
Zanotti, P.: LB935-444
Zaragoza, G.: LB1545-382
Zare, H.: 3360-260
Zaslavsky, O.: LB935-445, LB1545-358
Zauszniewski, J.: 890-216
Zavala, C. M: 655
Zazove, P.: 2010
Zebrak, K.: 2045
Zecevic, A.: LB935-394
Zechner, M.: 2435-306
Zeisel, J.: 500
Zeisel, J.: 2355-63
Zeki Al Hazzouri, A.: 3595
Zelalem, A.: 905-251
Zelinski, E. M: 925-317, 1401-284, LB2570-350
Zell, A. M: 1305-29, 4005
Zelner, J.: 925-337
Zeng, Y.: 910-271
Zettel-Watson, L.: 3365-272
Zgibor, J. C: 565

Zhai, F.: 1515
Zhan, L.: LB2570-324
Zhang, A.: 3250, 3275-25
Zhang, B.: LB1545-377
Zhang, C.: LB3620-425
Zhang, C.: 825-42
Zhang, F.: 845-120
Zhang, F.: LB3620-432
Zhang, G.: 3520
Zhang, J.: 845-123
Zhang, K.: 3090, 3290-54
Zhang, L.: LB935-388
Zhang, L.: 2115
Zhang, N.: 820-39, 830-76
Zhang, Q.: LB935-373
Zhang, Q.: LB1545-377
Zhang, Q.: 3320-133
Zhang, Q.: 1355-137
Zhang, S.: LB2570-322
Zhang, S.: 700
Zhang, T.: LB1545-387
Zhang, T.: 820-28
Zhang, W.: 3090, 3160, 3290-54
Zhang, W.: 1400-253, 2370-124
Zhang, W.: 1395-247
Zhang, W.: LB935-432
Zhang, X.: LB3620-377
Zhang, X.: 2375-146, 3380, 3605, LB935-415, LB3620-365
Zhang, X.: LB935-409
Zhang, X.: 830-67
Zhang, Y.: 1405-317, 1515
Zhang, Y.: 845-121
Zhang, Y.: 2165
Zhang, Y.: 3145
Zhang, Y.: 1425, 2315-14
Zhang, Z.: 2400-229
Zhao, E.: LB3620-361
Zhao, F.: LB3620-429
Zhao, L.: 2340-46
Zhao, L.: 2385-184
Zhao, S.: 740, 3535
Zhao, X.: 3390
Zhao, X.: 1355-137
Zhaoyang, R.: 4015
Zheng, D.: 1395-246
Zheng, H.: 825-47, LB935-364
Zheng, J.: 3505
Zheng, M.: 2400-225
Zheng, Q.: 740
Zheng, Q.: 1350-122
Zheng, Q.: 3330-179
Zheng, S.: 3505
Zheng, Y.: 1360-162
Zheng, Z.: 1395-242
Zhong, C.: 1402-313
Zhou, H.: 2360-99, 3325-166, LB935-381
Zhou, J.: 2410-256
Zhou, J.: 1402-302, 4020
Zhou, Y.: 3355-256
Zhou, Y.: 500
Zhou, Y.: 3110, 3350-242
Zhou, Y.: 3250
Zhu, H.: 1402-302
Zhu, Q.: 3345-231
Zhu, Y.: 1315-59, LB1545-443
Zia, G.: 1402-298, 3325-159
Zidar, D. A: 835-86
Ziegler, U.: 1402-315
Zigler, M.: 835-89
Zijlstra, G.: 565, 3035, 3270-5
Zimmer, R.: LB3620-421
Zimmerman, S.: 520, 3345-224
Zipunnikov, V.: LB935-446
Zisberg, A.: 2315-7, 3420, LB1545-375
Zissimopoulos, J.: 4155
Zmora, R.: 1385-210, 1401-278, 2355-71
Zmuda, J. M: 1425, 2370-125, 3170, 3275-24, LB1545-440, LB3620-337
Zou, M.: 820-28, 3325-172
Zubatsky, M.: 1020
Zucchelli, A.: 3170, 3325-173
Zulka, L.: 590
Zullo, A.: 4130
Zumo, J.: LB1545-402
Zuniga, J.: 3600
Zuniga, K. E: 3295-82
Zúñiga, F.: 2020
Zuo, D.: 3335-194
Zurlo, K.: 2030
Zwakhalen, S. M: 2380-164
Zweig, R.: 915-280
